# Metformin Inhibits NLR Family Pyrin Domain Containing 3 (NLRP)-Relevant Neuroinflammation *via* an Adenosine-5′-Monophosphate-Activated Protein Kinase (AMPK)-Dependent Pathway to Alleviate Early Brain Injury After Subarachnoid Hemorrhage in Mice

**DOI:** 10.3389/fphar.2022.796616

**Published:** 2022-03-17

**Authors:** Lei Jin, Fa Jin, Shenquan Guo, Wenchao Liu, Boyang Wei, Haiyan Fan, Guangxu Li, Xin Zhang, Shixing Su, Ran Li, Dazhao Fang, Chuanzhi Duan, Xifeng Li

**Affiliations:** Neurosurgery Center, Department of Cerebrovascular Surgery, The National Key Clinical Specialty, The Engineering Technology Research Center of Education Ministry of China on Diagnosis and Treatment of Cerebrovascular Disease, Guangdong Provincial Key Laboratory on Brain Function Repair and Regeneration, The Neurosurgery Institute of Guangdong Province, Zhujiang Hospital, Southern Medical University, Guangzhou, China

**Keywords:** metformin, neuroinflammation, NLRP3 inflammasome, neuron injury, subarachnoid hemorrhage

## Abstract

Neuroinflammation plays a key role in the pathogenesis of early brain injury (EBI) after subarachnoid hemorrhage (SAH). Previous studies have shown that metformin exerts anti-inflammatory effects and promotes functional recovery in various central nervous system diseases. We designed this study to investigate the effects of metformin on EBI after SAH. Our results indicate that the use of metformin alleviates the brain edema, behavioral disorders, cell apoptosis, and neuronal injury caused by SAH. The SAH-induced NLRP3-associated inflammatory response and the activation of microglia are also suppressed by metformin. However, we found that the blockade of AMPK with compound C weakened the neuroprotective effects of metformin on EBI. Collectively, our findings indicate that metformin exerts its neuroprotective effects by inhibiting neuroinflammation in an AMPK-dependent manner, by modulating the production of NLRP3-associated proinflammatory factors and the activation of microglia.

## Introduction

Subarachnoid hemorrhage (SAH) is a life-threatening subtype of stroke, characterized by high rates of mortality and poor prognoses (Laiwalla et al., 2016; Etminan et al., 2019). A previous study attributed the unfavorable prognoses of SAH patients to angiographic vasospasm (Crowley et al., 2008). However, clinical trials have shown that drugs directed against vasospasm failed to improve the poor prognoses of these patients (Macdonald et al., 2011; Macdonald et al., 2012). Recent studies have identified early brain injury (EBI), which occurs within the first 3 days of SAH, as one of the major causes of the unfavorable prognoses of SAH patients (Chen et al., 2014; Li et al., 2018). The EBI phase after SAH includes multiple pathophysiological processes. The overactivation of the inflammatory responses not only directly damages brain tissue but also contributes to the disruption of the blood–brain barrier and cell apoptosis, which further aggravate the brain injury (Fujii et al., 2013; Liu et al., 2019). A prospective observational study demonstrated that the inflammatory response is an independent predictor of unfavorable outcomes in SAH patients (Badjatia et al., 2015). Therefore, the suppression of neuroinflammation may be an effective strategy to mitigate brain injury after SAH.

The NLR family pyrin domain containing 3 (NLRP3) inflammasome is a multiprotein complex that regulates the innate immune inflammatory response (Zhou et al., 2011). The activation of the NLRP3 inflammasome causes the autocatalytic cleavage of pro-caspase 1, resulting in the processing and secretion of proinflammatory cytokines IL-1*β* and IL-18, which participate in the inflammatory response in multiple diseases (Mangan et al., 2018; Yang Y. et al., 2019). Recent studies have shown that the NLRP3 inflammasome contributes to the pathogenesis of EBI after SAH, and its suppression protects against brain injury (Dodd et al., 2021; Xu et al., 2021).

Adenosine-monophosphate-activated protein kinase (AMPK) is a sensor of cellular energy and modulates the status of many cellular processes (Meijer and Codogno, 2007). Recent advances in SAH research have demonstrated that the inhibition of NLRP3-inflammasome-associated neuroinflammation by activating AMPK reduces brain injury after SAH (Xu et al., 2019; Peng et al., 2020).

Metformin is widely used to treat patients with type 2 diabetes and metabolic syndrome because it safely and strongly enhances insulin sensitivity (Sanchez-Rangel and Inzucchi, 2017). A randomized controlled trial also reported that the concentrations of blood biomarkers predicting poor outcomes were significantly lower in patients with severe traumatic brain injury (TBI) treated with metformin than in untreated TBI patients (Taheri et al., 2019). Recent studies have shown that metformin exerts anti-inflammatory effects and alleviates ischemia–reperfusion injury by inhibiting the activation of the NLRP3 inflammasome (Jia et al., 2020; Zhang et al., 2020; Xian et al., 2021). In a recent study, we reported that HSP22 relieved SAH-induced EBI by salvaging the mitochondrial function in an AMPK–PGC1α-dependent manner (Fan et al., 2021). Moreover, metformin is be known to be one kind of activator of AMPK (Xie et al., 2011; Grégoire et al., 2018). To our knowledge, there have been no reports of the role of metformin in EBI after SAH. Therefore, in this study, we evaluated the effects of metformin on EBI after SAH and investigated the underlying mechanism.

## Materials and Methods

### Animals

Healthy adult male C57BL/6J mice (weighing 20–25 g) were obtained from the Animal Experiment Center of Southern Medical University (Guangzhou, China). The mice were housed under a 12-h light/dark cycle at a controlled temperature (22 ± 1°C) and humidity (60 ± 5%). The mice had free access to water and food. All experimental procedures involving animals were approved by the Southern Medical University Ethics Committee (Guangzhou, China) and conformed to the Guidelines of the National Institutes of Health on the Care and Use of Animals (China). The Animal Ethics Project Number is LAEC-2020-142.

### Experimental Design

**FIGURE 1:**
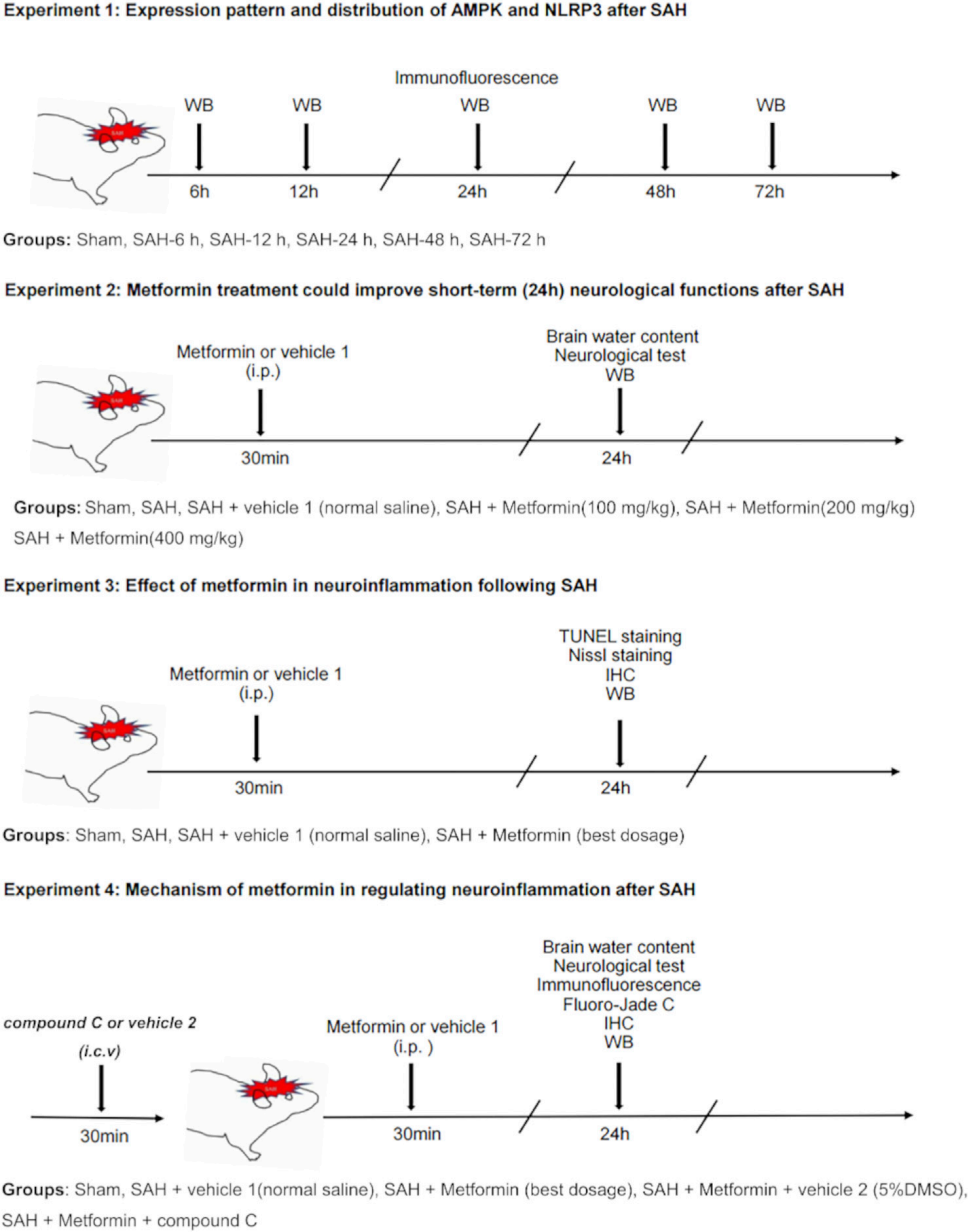
Experimental design and animal groups. Abbreviation: AMPK, adenosine 5′monophosphate-activated protein kinase; NLRP3, nucleotide-binding domain, leucine-rich containing family, pyrin domain-containing-3; SAH, subarachnoid hemorrhage; WB, western blot; IHC, immunohistochemistry; TUNEL, terminal deoxynucleotidyl transferase dUTP nick end labeling; i. p., intraperitoneal injection, i. c. v, intracerebroventricular injection.

This study included four separate experiments, as shown in Figure 1.

#### Experiment 1

Western blotting was used to determine the expression patterns of phosphorylated AMPK (p-AMPK), AMPK, and NLRP3 after SAH. The mice were randomly allocated into the six groups including the sham group and five SAH subgroups (at 6, 12, 24, 48, 72 h after SAH. *n* = 6). The left cerebral cortices from each group were collected for western blotting analysis. Double-labeled immunofluorescence was also used for the cellular localization of p-AMPK and NLRP3 in the SAH group (at 24 h after SAH. *n* = 3).

#### Experiment 2

To determine the optimal dose of metformin for subsequent experiments, a concentration gradient of metformin was evaluated based on a previous study (Ashabi et al., 2014). Mice were randomly divided into six groups: sham, SAH, SAH + vehicle 1 (normal saline), SAH + metformin (100 mg/kg), SAH + metformin (200 mg/kg), and SAH + metformin (400 mg/kg). Metformin was administered intraperitoneally 30 min after SAH. A neurological test (modified Garcia score) (*n* = 8) was performed and the brain water content of the mice (*n* = 8) was analyzed at 24 h after SAH. After the neurological score was determined, the mice were killed and brain samples collected to assay the brain water content. The effects of different doses of metformin on NLRP3 expression in the SAH (24 h) group were evaluated with western blotting (*n* = 6). The SAH + vehicle 1 group was given the same volume of normal saline intraperitoneally at the same time points as the metformin-treated mice after the induction of SAH. The SAH group was only treated surgically, with no other treatment. The sham group underwent the same surgical procedure without perforation of the blood vessel, with no other treatment.

#### Experiment 3

The optimal dose of metformin (200 mg/kg) determined in the experiment described above was used in subsequent experiments. Nissl staining (*n* = 5), terminal deoxynucleotidyl transferase dUTP nick end labeling (TUNEL) staining (*n* = 5), immunohistochemical staining (*n* = 5), and western blotting (*n* = 6) were used to evaluate the effects of metformin on brain injury and the expression of proinflammatory factors at 24 h after SAH. The mice were randomly divided into four groups: sham, SAH, SAH + vehicle 1, and SAH + metformin (200 mg/kg).

#### Experiment 4

To further investigate the mechanism by which metformin inhibits neuroinflammation after SAH, compound C (6-[4-(2-piperidin-1-ylethoxy) phenyl]-3-pyridin-4-ylpyrazolo [1,5-a] pyrimidine]), a selective inhibitor of AMPK signaling, was administered *via* the intracerebroventricular route 30 min before SAH, according to a previous study (Peng et al., 2020). The mice were divided into the sham, SAH + vehicle 1, SAH + metformin, SAH + metformin + vehicle 2 (5% dimethyl sulfoxide [DMSO]), and SAH + metformin + compound C groups. The brain water content (*n* = 8) and modified Garcia score (*n* = 8) were determined and western blotting (*n* = 6), immunofluorescent staining (*n* = 5), immunohistochemical staining (*n* = 5), and Fluoro-Jade C staining (*n* = 5) were performed at 24 h after SAH. The SAH + metformin + vehicle 2 and SAH + metformin + compound C groups were given the same volume of vehicle 2 and compound C intracerebroventricularly, respectively, at the same time points before SAH was induced.

### Subarachnoid Hemorrhage Mouse Model

A mouse model of SAH was generated with endovascular perforation, as described previously (Peng et al., 2020). Briefly, C57BL/6 J mice were anesthetized with 2% isoflurane in oxygen for 3 min and anesthesia was maintained with 1.0–1.5% isoflurane in 70% N_2_O and 30% O_2_ in a small-animal anesthesia system (Vet Equip, Pleasanton, CA, United States). A sharpened nylon suture was then inserted through the left external carotid artery to the common carotid artery and the internal carotid artery (ICA), ultimately perforating the intracranial bifurcation of the ICA. The sham-operated mice underwent the same procedures but the suture was withdrawn without puncture. During the operation, the body temperatures of the experimental animals were maintained at 37 ± 0.5°C with a heating pad.

### Drug Administration

Metformin (Sigma, PHR1084) was dissolved in normal saline and given by intraperitoneal injection 30 min after SAH ictus. The time points and method of drug delivery were based on previous reports (Ashabi et al., 2014; Peng et al., 2020). In accordance with a previous report (Peng et al., 2020), the selective AMPK inhibitor compound C (Apexbio, B3252) was dissolved in 5% DMSO, and a working solution (5 μg in 5 μL) was injected into the intracerebroventricular cavity 30 min before the induction of SAH.

### Intracerebroventricular Injection

Intracerebroventricular injections were conducted as previously described (Peng et al., 2020). Briefly, a 10 μL microsyringe (Shanghai High Pigeon Industry & Trade Co., Ltd., Shanghai, China) was inserted into the left lateral ventricle through a small cranial burr hole, which was drilled 0.3 mm posterior, 1.0 mm lateral, and 2.5 mm deep relative to the bregma. Compound C or vehicle 2 were administered slowly into the left lateral ventricle 30 min before surgery. The microsyringe was left *in situ* for an additional 10 min after the administration of the compound C or vehicle 2, and then withdrawn slowly. The burr hole was sealed with bone wax.

### Determination of Neurological Scores and Subarachnoid Hemorrhage Grades

The neurological scores (neurobiological deficits) were determined by an independent investigator blinded to the procedural information, using the modified Garcia test (Garcia et al., 1995). The Garcia score consists of six sensorimotor tests: spontaneous activity, spontaneous movement of all limbs, forelimb outstretching, climbing, touching the trunk, and vibrissal touch. Each test was scored from 0 to 3, and the total scores ranged from 0 to 18. A higher score indicated a milder neurological deficit.

The severity of SAH was evaluated blindly at 24 h after SAH, with a previously reported SAH grading system (Sugawara et al., 2008). Briefly, the brain basal cistern was divided into six parts, and each part was scored from 0 to 3 based on the amount of blood clotting present. The total score for the six parts represented the SAH grade. SAH mice with SAH grade ≤7 were excluded from this study.

### Brain Water Content

At 24 h after SAH, the mice were killed and their brains quickly removed, and divided into the left hemisphere, right hemisphere, cerebellum, and brain stem. Each segment was weighed immediately to determine their wet weight (WW), and the samples were then dried at 105°C for 72 h to determine their dry weight (DW). The brain water content was calculated as [(WW − DW)/WW] × 100%.

### Nissl Staining

Nissl staining was used to evaluate neuronal damage in the ipsilateral cortex, as described previously (Deng et al., 2021). Briefly, at 24 h after SAH, the mice were deeply anesthetized and transcardially perfused with 50 ml of phosphate-buffered saline (PBS; 0.1 M) followed by 50 ml of 4% paraformaldehyde (PFA; pH 7.4). The brain samples were removed quickly and postfixed in 4% PFA for 24 h at 4°C. After the brains were embedded in paraffin, they were cut into serial coronal sections (10 μm thick). The brain slices were deparaffinized and rehydrated. The slices were immersed in methyl violet solution (Nissl Stain Solution, G1432, Solarbio, Beijing). The slices were then dehydrated in 100% alcohol and washed with xylene. Images were obtained and analyzed under a light microscope (Leica-DM2500, Wetzlar, Germany) by a blinded investigator.

### Immunofluorescent Staining

Brain coronal slices (4 μm thick) were prepared as described previously (Liu et al., 2019), and deparaffinized in xylene, rehydrated in a graded series of alcohol, and washed with PBS (0.01 M, pH 7.4). After antigen retrieval, the brain slices were blocked with 5% donkey serum for 1 h at room temperature. The sections were incubated overnight at 4°C with the following primary antibodies: rabbit anti-p-AMPK (diluted 1:200; AF3423, Affinity); rabbit anti-NLRP3 (diluted 1:200; NBP1-77080SS, Novus); mouse anti-NEUN (diluted 1:500; ab104224, Abcam); mouse anti-IBA1 (diluted 1:200; GB12105, Servicebio); mouse anti-GFAP (diluted 1:200; GB12096, Servicebio), and mouse anti-GFAP (diluted 1:200; GA5, Cell Signaling Technology). The next day, the sections were washed with PBS and incubated for 1 h at room temperature with secondary antibodies: Alexa-Fluor-555-conjugated donkey anti-rabbit IgG (diluted 1:500; A31572, Invitrogen) and Alexa-Fluor-488-conjugated donkey anti-mouse IgG (diluted 1:500; A21202, Invitrogen). The sections were washed three times with PBS and stained with 4′,6-diamidino-2-phenylindole (DAPI) for 10 min at room temperature. The sections were observed and images captured under a fluorescence microscope (Nikon, TI2-E, Japan). To evaluate the numbers of IBA1-positive cells, three random fields in the ipsilateral cortex from three sections per brain were examined. Data are expressed as the average numbers of IBA1-positive cells per field in cells/mm^2^.

### Immunohistochemical Staining (IHC)

IHC was used to verify NLRP3 and IBA1 (a microglial marker in the brain) expression in the ipsilateral cortex. The deparaffinized and rehydrated coronal brain slices (4 μm thick) were prepared as described above. The slices were incubated with 3% H_2_O_2_ for 10 min at room temperature to quench any endogenous peroxidase activity, and then blocked with 5% goat serum for 20 min at room temperature. The slices were then incubated overnight at 4°C with the following primary antibodies: rabbit anti-NLRP3 (diluted 1:50; NBP1-77080SS, Novus) and mouse anti-IBA1 (diluted 1:100; GB12105, Servicebio). The next day, the brain slices were washed with PBS and incubated for 20 min at room temperature with biotinylated goat anti-rabbit IgG (diluted 1:100; ZSGB-Bio, Beijing, China) or goat anti-mouse IgG (diluted 1:100; ZSGB-Bio). The brain slices were then incubated with horseradish peroxidase (HRP)–streptavidin reagent for 10 min and stained with 3,3′-diaminobenzidine peroxidase substrate. Images were obtained with a light microscope (Leica-DM2500, Wetzlar).

### Fluoro-Jade C Staining

Fluoro-Jade C (FJC) staining was used to identify degenerate neurons, as previously described (Xu et al., 2021), but with modification. Briefly, the brain slices were immersed in an alcohol solution (1% sodium hydroxide in 80% ethanol) and then in 70% ethanol, and then immersed in 0.06% potassium permanganate solution for 10 min. The slices were then incubated in a 0.0001% working solution of FJC (Millipore, Darmstadt, Germany) for 30 min. Images were obtained under a fluorescence microscope and analyzed by a blinded observer. FJC-positive cells were counted in three different fields of the ipsilateral cortex in three sections per mouse. Data are expressed as the ratio of FJC-positive cells to DAPI-positive cells.

### TUNEL Staining

Cell apoptosis was detected with a TUNEL kit (Beyotime, China) at 24 h after SAH in strict accordance with the manufacturer’s instructions. Briefly, the deparaffinized and rehydrated coronal brain slices were prepared as described above. After the brain slices were washed with PBS, they were incubated with TUNEL mixture for 1 h at room temperature, and then stained with DAPI. The results of TUNEL staining were observed and analyzed in the same way as FJC staining. The data are expressed as the ratio of TUNEL-positive cells to DAPI-positive cells.

### Western Blotting

Western blotting was performed as previously described (Luo et al., 2020). Samples of the left cerebral cortices of the mice were collected after SAH. The tissues were lysed with RIPA lysis buffer (Cwbio, Guangzhou, China) to extract the proteins from the brain samples. Equal amounts of protein from different samples were loaded and separated on a 10% SDS-PAGE gel (Cwbio), and then transferred to a polyvinylidene difluoride filter membrane (Millipore, United States). The membranes were blocked with 5% nonfat milk for 3 h at room temperature and incubated overnight at 4°C with the following primary antibodies: anti-p-AMPK (diluted 1:1000; 40H9, Cell Signaling Technology), anti-AMPK antibody (diluted 1:1000; D5A2, Cell Signaling Technology), anti-NLRP3 (diluted 1:1000; 19771-1-AP, Proteintech), anti-cleaved caspase 1 (diluted 1:1000; E2G2I, Cell Signaling Technology), anti-IL-18 (diluted 1:1000; BS6823, Bioworld Technology), anti-IL-1*β* (diluted 1:1000; BS3506, Bioworld Technology), and anti-*β*-actin (diluted 1:1000; AP0060, Bioworld Technology). The membranes were washed three times for 10 min each in Tris-buffered saline containing 0.1% Tween 20, and then with HRP-conjugated goat anti-rabbit IgG (1:10000; Cwbio) at room temperature for 1 h. After the blots were washed, the proteins were visualized with an ECL Western blotting detection system (WBKLS0100, Millipore, United States) and analyzed with the ImageJ software (ImageJ 1.5, National Institutes of Health, Bethesda, MD, United States). *β*-Actin was used as the internal control.

### Statistical Analysis

All data are presented as means ± standard deviations (SD) and all statistical analyses were performed with the SPSS 19.0 software (SPSS, IBM, Armonk, NY, United States). Student’s *t* test was used to determine the statistical significance of differences between two groups and one-way analysis of variance (ANOVA) followed by Tukey’s post hoc test was used for comparisons of multiple groups. Statistical significance was accepted at *p* < 0.05. Investigators were blinded to the identity of the groups during the whole experiment.

## Results

### Subarachnoid Hemorrhage Model and Mortality

**FIGURE 2:**
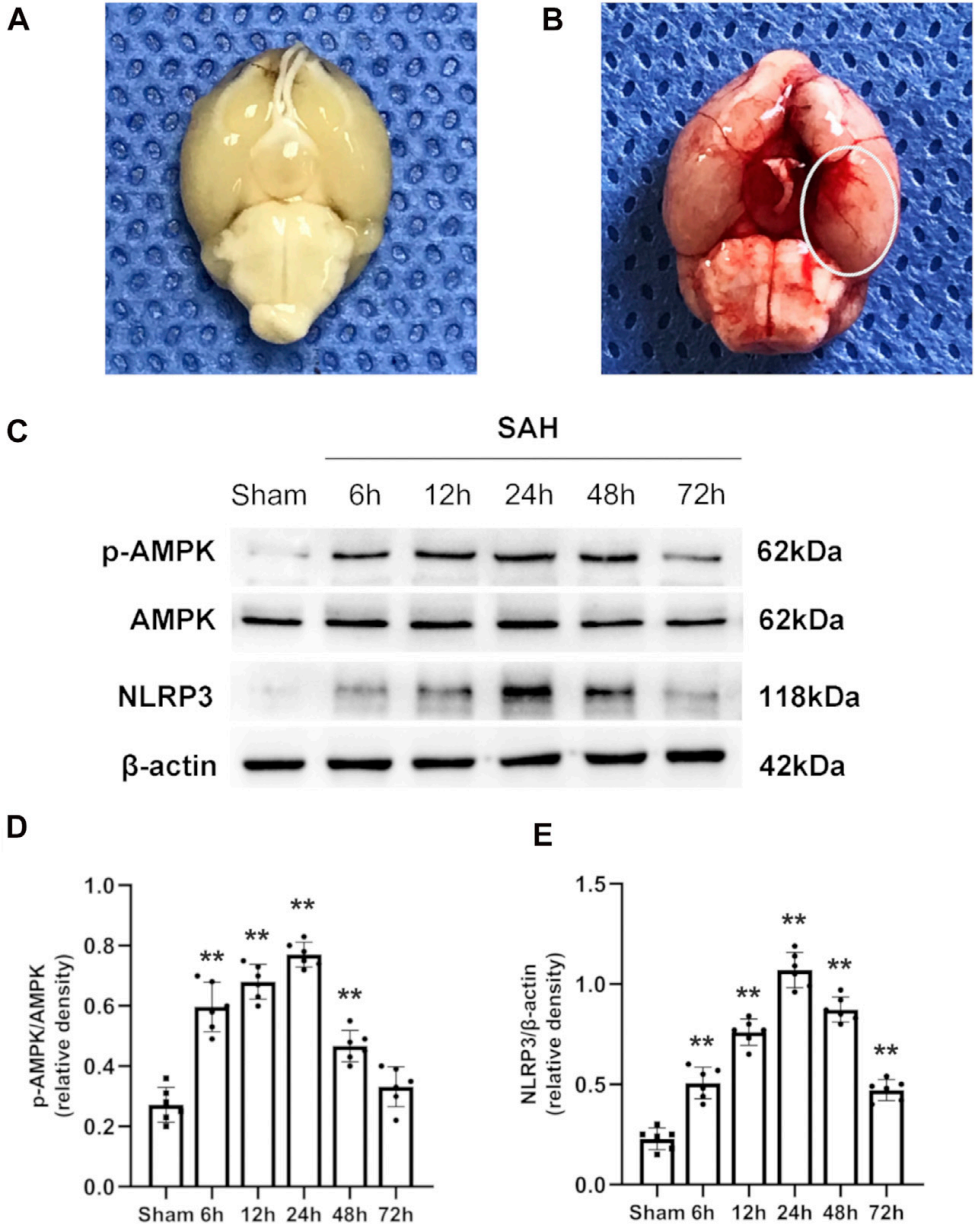
Typical images of SAH model and expression alterations of p-AMPK/AMPK and NLRP3 in the ipsilateral hemisphere after SAH. **(A,B)** Representative photographs of the bottom of mice brains from sham and 24 h after SAH. The area within the white oval was specifically used to observe for immunostaining. **(C)** Representative western blot images. **(D,E)** Quantitative analysis of western blot. Data are expressed as the mean ± SD using one-way ANOVA followed by Tukey’s post hoc test (**p < 0.01 vs Sham group, *n* = 6 per group).

The total number of mice used in this study was 304, including those that died or were excluded for each group. The overall mortality rates in the sham-operated and SAH mice were 0 and 19.7%, respectively (Supplementary Figure S1B). Typical images of the brains from mice in the sham and SAH groups are shown in Figures 2A,B. Previous studies indicated that the inferior basal temporal lobe was always stained with blood and shows the most significant molecular biological changes relative to the control animal. Therefore, we mainly evaluated the basal temporal lobe of the left hemisphere adjacent to the clotted blood (Park et al., 2004; Zhang et al., 2021) (Figure 2B). It is noteworthy that the mice treated with metformin (400 mg/kg) after SAH showed an obvious increase in death risk relative to that in the mice treated with other doses of metformin (Supplementary Figures S2A,B). Therefore, 400 mg/kg metformin was not included in subsequent experiments.

### Temporal Pattern of NLRP3 and p-AMPK After Subarachnoid Hemorrhage Induction

**FIGURE 3:**
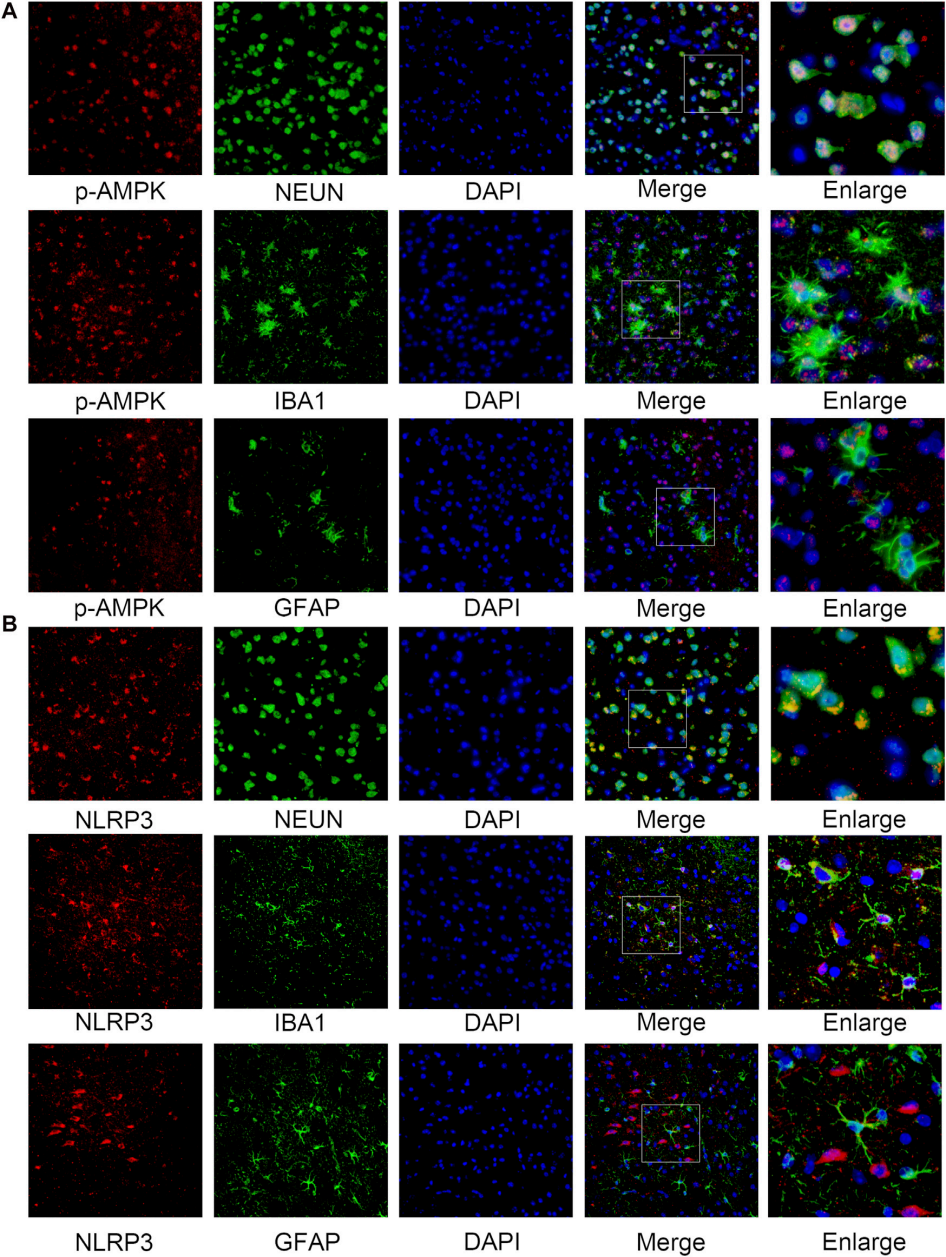
Cellular localization of p-AMPK and NLRP3 in the ipsilateral hemisphere 24 h after SAH. **(A)** Representative images of co-immunofluorescence staining of p-AMPK (red) with neurons (NEUN, green), astrocytes (GFAP, green), and microglia (IBA1, green) in the ipsilateral cortex at 24 h after SAH. Nuclei are stained with DAPI (blue) (Magnification = ×400, scale bar = 50 um, *n* = 3 per group). **(B)** Representative images of co-immunofluorescence staining of NLRP3 (red) with the same aforesaid conditions. Enlarged images come from the areas of a white rectangle within merge images.

The western blotting results indicated that the protein levels of p-AMPK and AMPK increased after SAH, peaked at 24 h, and then gradually decreased until 72 h relative to those in the sham group (Figures 2C,D). Simultaneously, the expression of NLRP3 was similar to that of p-AMPK and AMPK (Figures 2C,E). Double immunostaining for p-AMPK and NEUN, IBA1, or GFAP demonstrated that p-AMPK was expressed in neurons and microglia cells at 24 h after SAH. However, astrocytes did not immunostain for p-AMPK (Figure 3A). NLRP3 was also observed in neurons and microglial cells at 24 h after SAH. Astrocytes did not immunostain for NLRP3 (Figure 3B).

### Metformin Administration Improved Short-Term Neurological Functions and Attenuated Brain Edema at 24 h After Subarachnoid Hemorrhage

**FIGURE 4:**
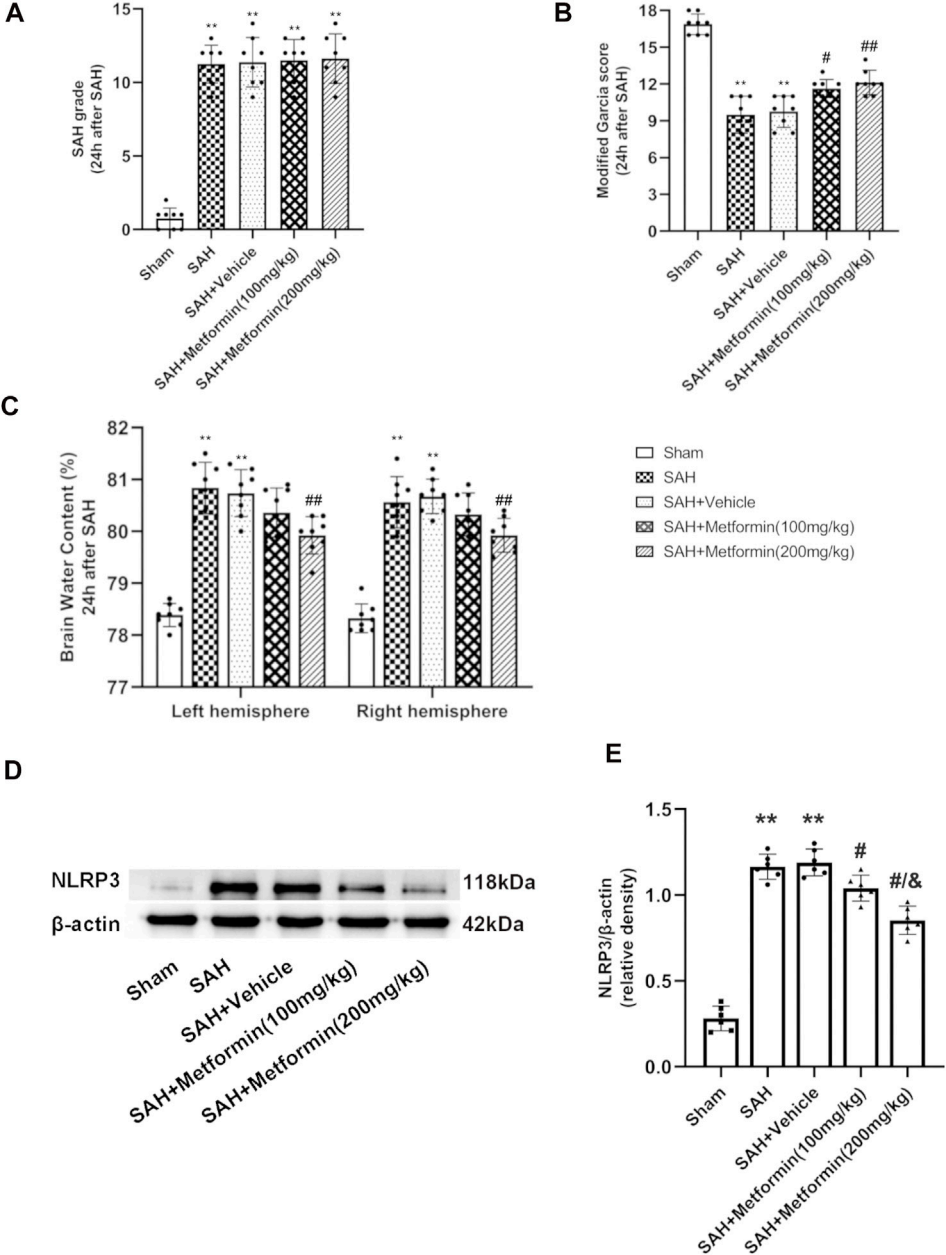
Metformin attenuates short-term neurological deficit and brain edema at 24 h after SAH. **(A)** The SAH grades for each group. **(B)** Modified Garcia scores for each group. **(C)** Quantification of brain water content 24 h after SAH. **(D)** Representative Western blot images. **(E)** Quantitative analyses of NLRP3. N = 6 for each group. Bars represent mean ± SD. **p < 0.01 vs. sham group. ^#^ *p* < 0.05, ^##^ *p* < 0.01 vs. SAH + Vehicle (normal saline) group. ^&^ *p* < 0.05 vs. SAH + Metformin (100 mg/kg).

Besides the sham group, no significant differences were detected in the SAH grade scores among the SAH groups (Figure 4A). The mice in the SAH group and SAH + vehicle group showed severe neurological impairment and higher brain water contents than the mice in the sham group at 24 h after SAH. The administration of both 100 mg/kg and 200 mg/kg metformin improved the neurological performance relative to that in the SAH + vehicle group. The modified Garcia test indicated that the SAH-affected mice that received metformin showed a smaller neurological deficit than the mice in the untreated SAH group, but no significant differences were observed in mice treated with different doses of metformin (Figure 4B). The brain water content results indicated that 200 mg/kg metformin significantly mitigated brain edema relative to that in the SAH + vehicle group, whereas 100 mg/kg metformin did not (Figure 4C). The western blotting results showed that metformin treatment significantly downregulated the SAH-enhanced level of NLRP3 protein 24 h after SAH, and that 200 mg/kg metformin was superior to 100 mg/kg metformin in this regard (Figures 4D,E). No significant differences in blood glucose were observed between the mice treated with various doses of metformin and the SAH groups (Supplementary Figure S3). Therefore, based on all these results, 200 mg/kg metformin was selected for the subsequent experiments.

### Metformin Alleviated Cell Apoptosis and Ameliorated Neuronal Injury at 24 h After Subarachnoid Hemorrhage

**FIGURE 5:**
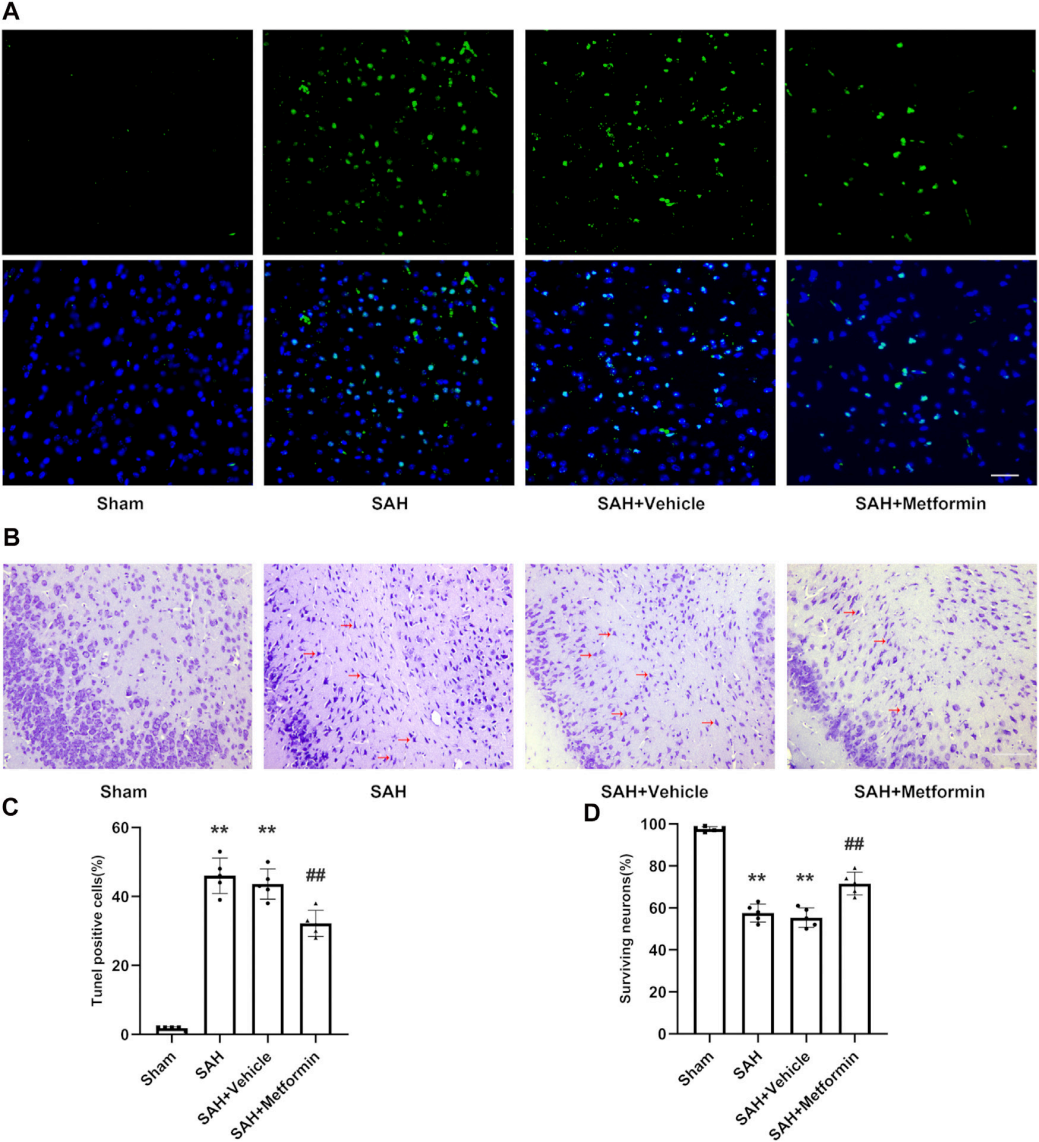
Metformin inhibits cell apoptosis and promotes neuron survival in the cortex after SAH. Abbreviation: Met = metformin. **(A)** Typical photomicrographs of TUNEL staining (Magnification = ×400, scale bar = 50 um, *n* = 5 per group). **(C)** Quantitative analysis of TUNEL staining. **(B)** Representative photomicrographs of Nissl staining. Compared to normal neurons, impaired neurons show shrunken cell body and staining darker nuclei. The representative form of the impaired neurons was marked with a red arrow (Magnification = ×200, scale bar = 100 um, *n* = 5 per group). **(D)** The quantitative analysis of Nissl staining. Data are shown as mean ± SD. **p < 0.01 vs. sham group. ^##^ *p* < 0.01 vs. SAH + Vehicle (normal saline) group.

According to the TUNEL staining results, the sham group showed negligible TUNEL-positive cells. By contrast, the SAH group and SAH + vehicle group showed markedly increased TUNEL-positive cells. Metformin treatment reduced the proportion of apoptotic cells after SAH (Figures 5A,C). Nissl staining was used to further assess the effects of metformin on neuronal injury after SAH. The sham group showed negligible neuronal death. The proportion of surviving neurons was significantly reduced in the SAH and SAH + vehicle groups. However, metformin treatment markedly reduced the neuronal death caused by SAH (Figures 5B,D).

### Metformin Administration Repressed the Activation of the NLRP3 Inflammasome and the Expression of the Relevant Inflammatory Cytokines at 24 h After Subarachnoid Hemorrhage

**FIGURE 6:**
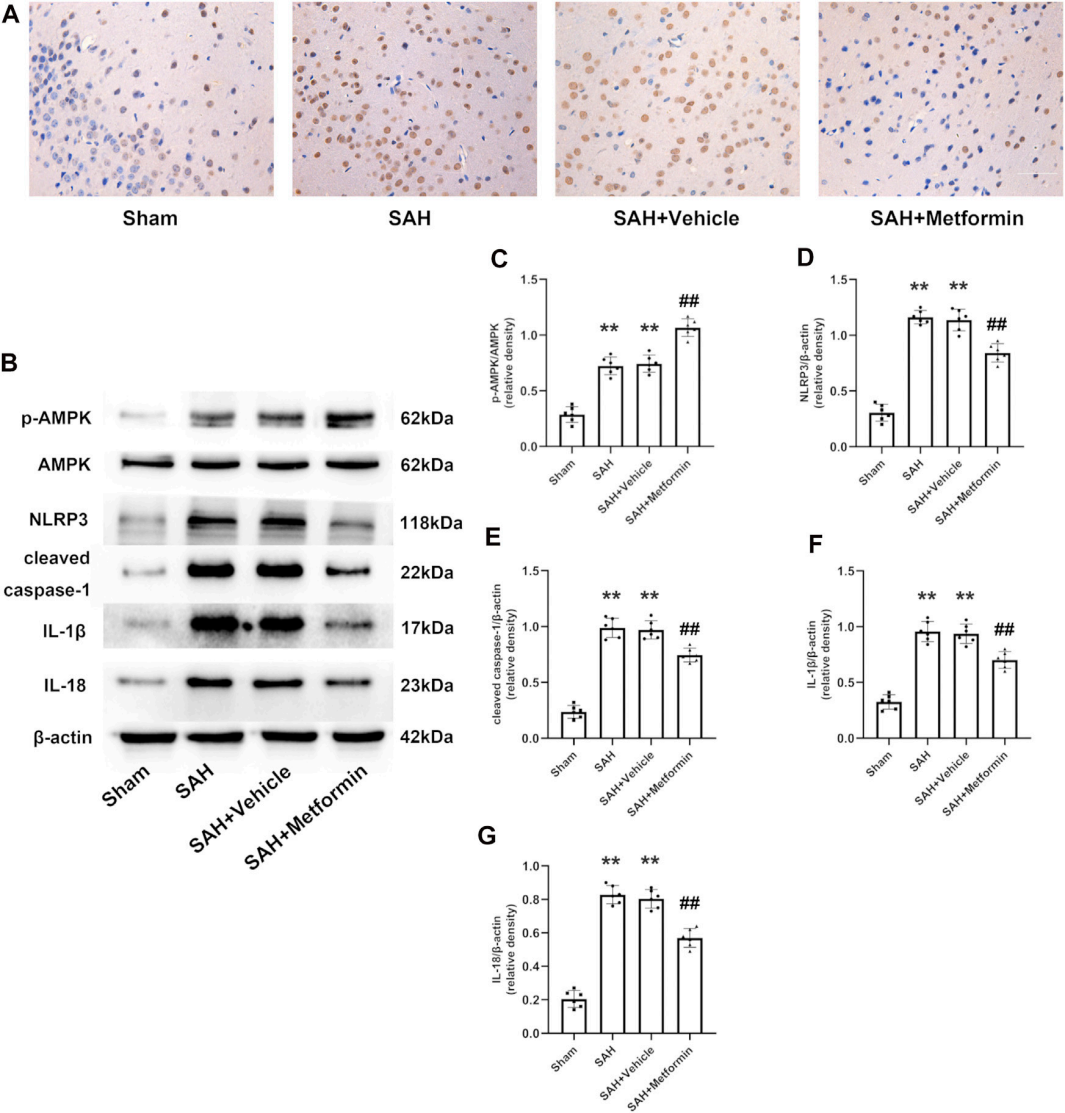
Metformin suppresses NLRP3-associated inflammatory response. **(A)** Representative images of immunohistochemical staining (Magnification = ×400, scale bar = 50 um, *n* = 5 per group). **(B)** Representative western blot images. **(C–G)** Quantitative analysis of western blot. *N* = 6 for each group. Data are shown as mean ± SD. **p < 0.01 vs. sham group. ^##^ *p* < 0.01 vs. SAH + Vehicle (normal saline) group.

Immunohistochemical staining revealed that the expression of NLRP3 was elevated at 24 h after SAH, but this increase was inhibited by metformin (Figure 6A). The western blotting results showed that the expressions of p-AMPK, NLRP3, cleaved caspase 1, cleaved IL-1*β*, and cleaved IL-18 were clearly increased in the SAH and SAH + vehicle groups relative to that in the sham group. Metformin treatment further enhanced the level of p-AMPK, but reduced the levels of NLRP3, cleaved caspase 1, cleaved IL-1*β*, and cleaved IL-18 relative to those in the SAH mice not treated with metformin (Figures 6B–G).

### Inhibition of Adenosine-Monophosphate-Activated Protein Kinase Weakened the Protective Effects and Anti-inflammatory Effects of Metformin at 24 h After Subarachnoid Hemorrhage

**FIGURE 7:**
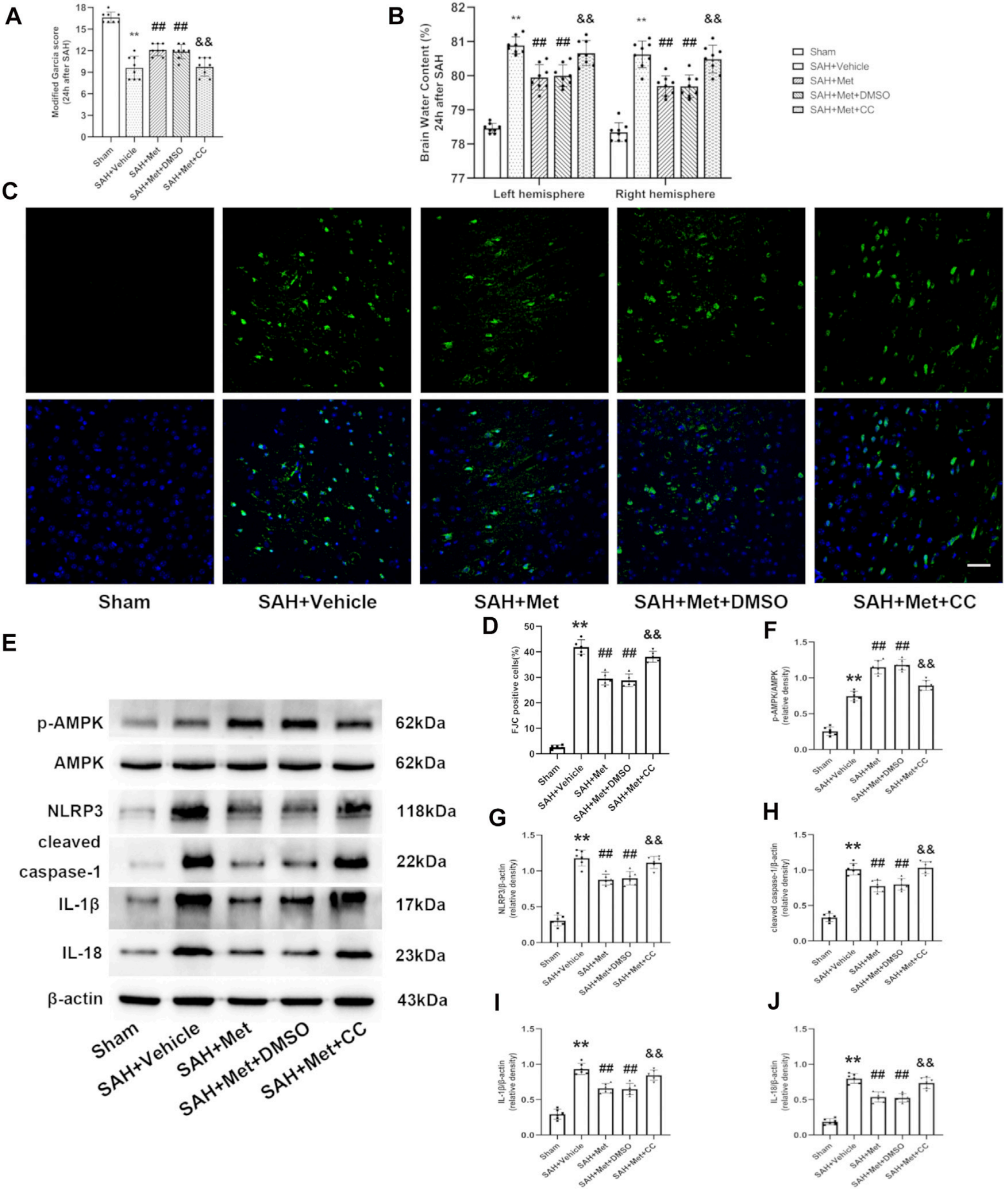
Inhibition of AMPK abolishes the protective effects of metformin after SAH. Abbreviation: Met, metformin, CC, compound C. **(A)** Modified Garcia scores for each group. **(B)**The quantification of brain water content 24 h after SAH. *N* = 8 for each group. **(C)** Representative images of FJC staining (Magnification = ×400, scale bar = 50 um, *n* = 5 per group). **(D)** Quantitative analysis of FJC. **(E)** Representative western blot images. **(F–J)** Quantitative analysis of western blot. N = 6 for each group. Data are shown as mean ± SD. **p < 0.01 vs. sham group. ^##^ *p* < 0.01 vs. SAH + Vehicle (normal saline) group. ^&&^ *p* < 0.01 vs. SAH + Metformin + DMSO group.

To determine whether AMPK is required for the protection against EBI offered by metformin after SAH, compound C was administered by intracerebroventricular injection. The metformin-associated alleviation of neurobehavioral deficits and the reduction in the brain water content were abolished by compound C (Figures 7A,B). FJC staining was used to further clarify the role of AMPK in metformin-ameliorated neuronal death after SAH. The inhibition of AMPK by compound C reversed the protective role of metformin against neuronal injury compared with that in the SAH + metformin group (Figures 7C,D). The western blotting results also indicated that the effects of metformin in increasing AMPK activation and reducing the expression of NLRP3, cleaved caspase 1, cleaved IL-1*β*, and cleaved IL-18 were suppressed by compound C (Figures 7E–J).

### The Inhibition of Adenosine-Monophosphate-Activated Protein Kinase Blocked the Inhibitory Effect of Metformin on Microglial Activation at 24 h After Subarachnoid Hemorrhage

**FIGURE 8:**
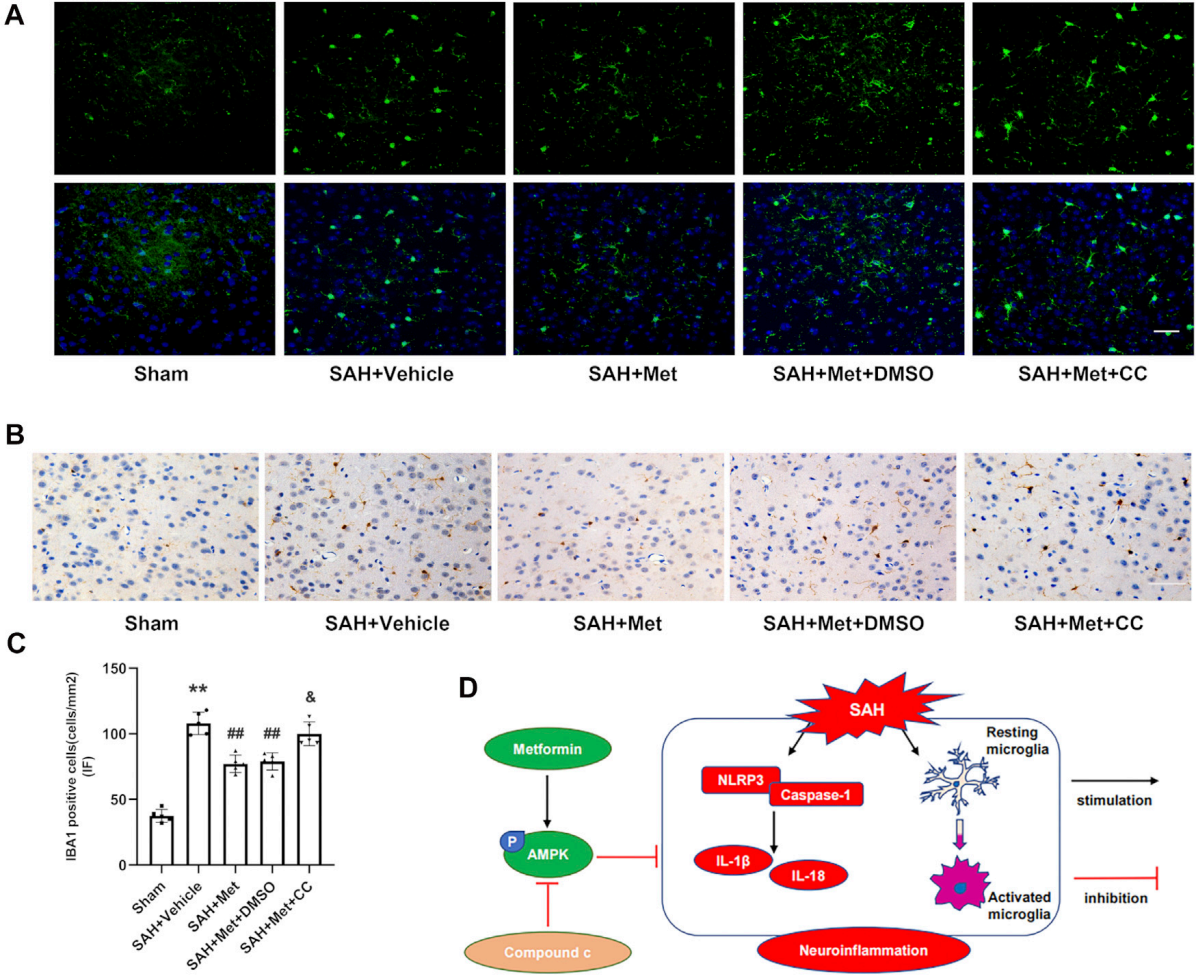
Suppression of AMPK reverses the inhibitory effects of metformin on microglial activation after SAH. **(A,C)** Representative images of immunofluorescence staining and quantification of IBA1 activation. **(B)** Representative images of immunohistochemical staining of IBA1 activation (Magnification = ×400, scale bar = 50 um, *n* = 5 per group). Data are shown as mean ± SD. **p < 0.01 vs. sham group. ^##^ *p* < 0.01 vs. SAH + Vehicle (normal saline) group. ^&^ *p* < 0.05 vs. SAH + Metformin + DMSO group. **(D)** Graphical abstract of how metformin regulates SAH-induced neuroinflammation *via* AMPK-dependent pathway.

Immunofluorescent staining showed that microglia were markedly increased in number and activated (presenting as larger cell bodies with shorter processes) in the SAH + groups compared with those in the sham group, and that the administration of metformin significantly reduced the number of IBA1-positive cells and mitigated the overactivation of microglia. However, compound C blocked these effects of metformin on the regulation of microglia, which was confirmed with immunohistochemical staining (Figures 8A–C).

## Discussion

In this study, we have demonstrated the beneficial effects of metformin on EBI and neuroinflammation after SAH. The important findings of our experiments are as follows. 1) The levels of p-AMPK and NLRP3 increased markedly and peaked at 24 h after SAH. Both proteins colocalized with neurons and microglia, but no fluorescent signal was detected in astrocytes. 2) The administration of metformin significantly alleviated the neurological deficit and reduced brain edema after SAH. 3) The use of metformin after SAH enhanced AMPK activation and suppressed the expression of NLRP3-relevant inflammatory mediators, thereby mitigating cell apoptosis and neuronal injury. 4) The inhibition of AMPK activation with compound C reversed the favorable effects of metformin on EBI and the inflammatory response. 5) Metformin treatment clearly suppressed microglial activation after SAH, but compound C abolished this effect. Taken together, our findings demonstrate that metformin exerts protective effects against SAH-induced EBI partly by upregulating AMPK activation, thus downregulating the NLRP3 inflammasome-associated inflammatory reaction and inhibits microglial overactivation (Figure 8D).

An increasing number of studies support the notion that the inflammatory response is one of the key causes of EBI and is associated with severe complications after SAH (Fujii et al., 2013; Lucke-Wold et al., 2016; Wang et al., 2021). Recent studies have shown that the inhibition of NLRP3 activation significantly ameliorates SAH-induced brain injury and delays cerebral vasospasm, indicating that NLRP3-inflammasome-associated inflammatory factors play a key role in EBI after SAH (Li et al., 2017; Dodd et al., 2021; Zhang et al., 2021). Consistent with a previous study (Zhang et al., 2017; Hu et al., 2021), our data show that the expression of NLRP3 is elevated and peaks at 24 h after SAH, and that NLRP3 is expressed in microglia.

Metformin has recently been reported to exert its protective effects through an anti-inflammatory mechanism by inhibiting the NLRP3 inflammasome in multiple diseases (Bullón et al., 2016; Yang F. et al., 2019). In the present study, we found that the administration of metformin after SAH ameliorated neurobehavioral deficits, brain edema, cell apoptosis, and neuronal injury, and reduced the expression of NLRP3, cleaved caspase 1, IL-1*β*, and IL-18.

AMPK acts as an endocellular energy sensor. Metformin is well-recognized as an activator of AMPK (Hawley et al., 2010; Hardie et al., 2012; Jin et al., 2014). In our earlier study, we demonstrated that the activation of AMPK consistently reduces brain injury by ameliorating mitochondrial dysfunction after SAH (Fan et al., 2021). Therefore, we speculated that metformin may improve brain injury after SAH by activating AMPK. In our experiments, NLRP3 and p-AMPK both localized in neurons and microglial cells, and peaked at 24 h after SAH, consistent with the results of other studies (Xu et al., 2020; Fan et al., 2021). The similar expression trends and cellular localization of p-AMPK and NLRP3 suggested that they interact with each other. It has been reported that AMPK inhibits NLRP3 expression by activating autophagy (Yang F. et al., 2019; Youssef et al., 2021). Previous studies demonstrated that AMPK activation alleviates brain injury by suppressing the activation of the endoplasmic-reticulum-stress-associated TXNIP/NLRP3 inflammasome (Li et al., 2015; Xu et al., 2019). Our results indicated that the administration of metformin enhanced the activation of AMPK, which was blocked by compound C. The protective action of metformin treatment against neurological deficit, brain edema, neuronal injury, and NLRP3-relevant inflammatory reactions after SAH were also reversed by compound C. This is consistent with recent studies that have reported that the inhibition of AMPK activation reduces the brain injury after SAH (Xu et al., 2019; Peng et al., 2020).

Microglia, the resident immunocompetent and phagocytic cells of the central nervous system, produce inflammatory mediators and contribute to the neuroinflammation that occurs after SAH (Gris et al., 2019; Zheng et al., 2020). Therefore, the suppression of microglial activation limits the harmful inflammatory response and attenuates inflammation-induced EBI (Xie et al., 2017; Peng et al., 2019; Liu et al., 2020). Recent studies have provided evidence supporting the suppression of microglial activation by metformin through the activation of AMPK, both *in vivo* and *in vitro* (Pan et al., 2016; Inyang et al., 2019). Consistent with previous studies, our results indicate that metformin markedly reduces the number of IBA1-positive cells, whereas compound C abolishes this effect.

Although this study demonstrates the value of metformin in attenuating EBI in a mouse model of SAH, several limitations should be noted. First, we only evaluated the anti-inflammatory characteristics of metformin, and did not examine its possible capacity to affect other regulatory mechanisms in our mouse model of SAH. Second, previous study has shown that the prolonged use of metformin after brain injury promotes angiogenesis and long-term functional recovery (Dadwal et al., 2015). Here, only the effects of a single post-SAH intraperitoneal injection of metformin were investigated, so the long-term outcomes of multiple systemic metformin treatments over different time courses should be examined in future studies.

The recent literature has hinted that metformin plays a beneficial role in improving the poor prognoses of patients with brain injury. A multicenter retrospective analysis indicated that stroke patients with diabetes who were treated with metformin showed less-severe stroke on admission and better functional outcomes at 3 months than stroke patients with diabetes who were not treated with metformin (Westphal et al., 2020). Therefore, whether metformin can improve the long-term outcomes of SAH and whether it exerts beneficial clinical effects on SAH patients must also be considered in future studies.

In conclusion, our findings support the notion that metformin exerts a neuroprotective effect by inhibiting the NLRP3-associated neuroinflammatory response and suppressing microglial activation in an AMPK-dependent manner. Therefore, metformin may provide a potential therapeutic intervention for improving EBI after SAH.

## Data Availability

The original contributions presented in the study are included in the article/Supplementary Material, further inquiries can be directed to the corresponding author.
