# Supplementary figures and images for: Metformin Inhibits NLR Family Pyrin Domain Containing 3 (NLRP)-Relevant Neuroinflammation via an Adenosine-5′-Monophosphate-Activated Protein Kinase (AMPK)-Dependent Pathway to Alleviate Early Brain Injury After Subarachnoid Hemorrhage in Mice (part 1 of 2)
Source: Front Pharmacol. 2022 Mar 17;13:796616. doi: 10.3389/fphar.2022.796616 (PMC8969021; doi:10.3389/fphar.2022.796616)

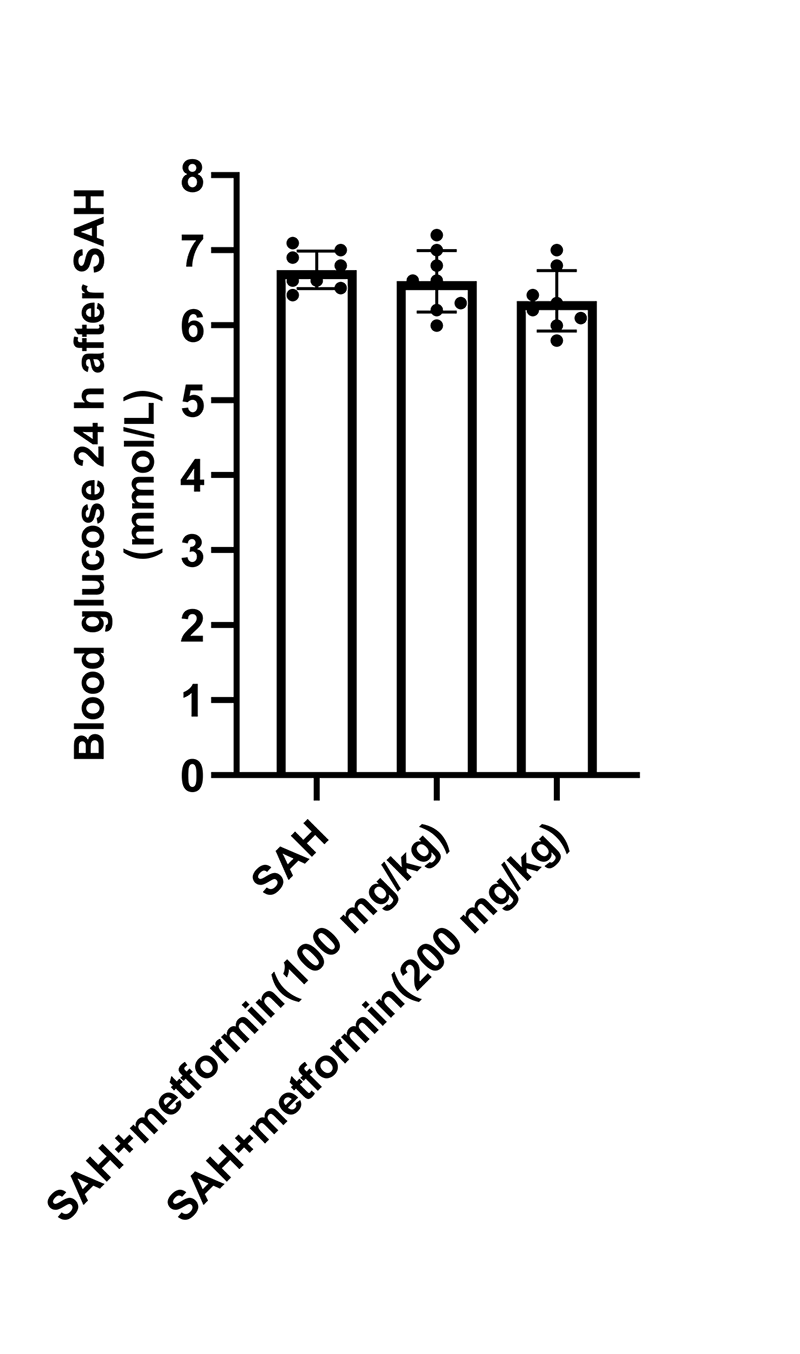

Supplement: Supplementary file 1 [file Image3.TIF]

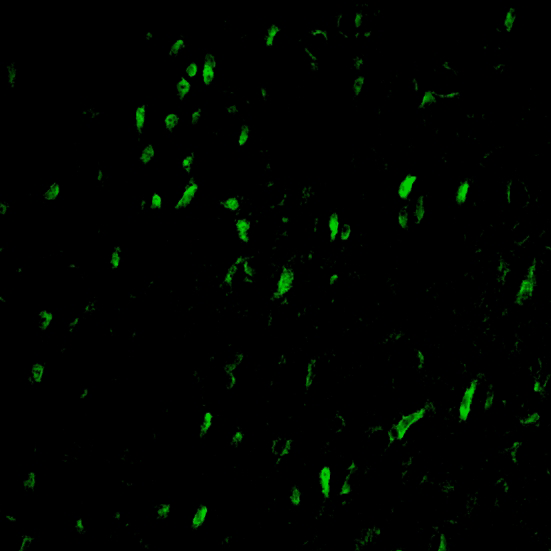

Supplement: Supplementary file 2 [file DataSheet1.ZIP › data sheet for review purpose only (1)/Original date for review purpose only (1)/FJC/Figure 7-SAH+Met+CC-FITC.tif]

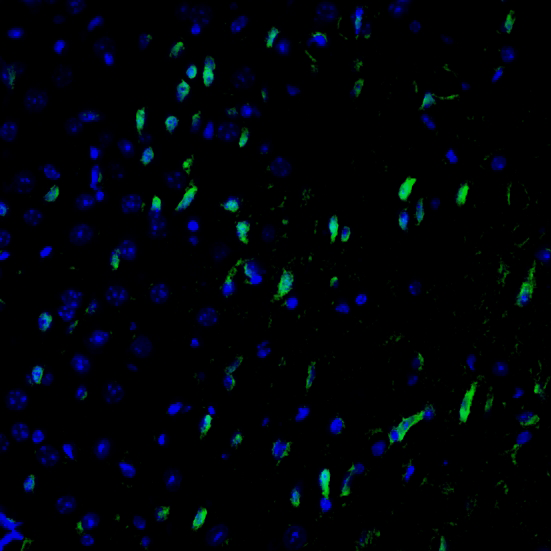

Supplement: Supplementary file 2 [file DataSheet1.ZIP › data sheet for review purpose only (1)/Original date for review purpose only (1)/FJC/Figure 7-SAH+Met+CC-merge.tif]

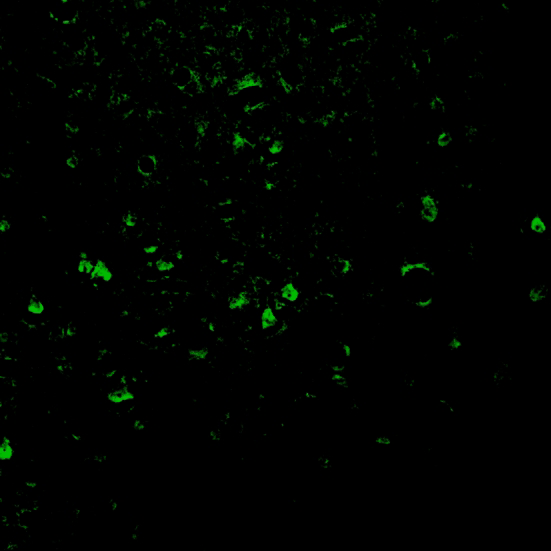

Supplement: Supplementary file 2 [file DataSheet1.ZIP › data sheet for review purpose only (1)/Original date for review purpose only (1)/FJC/Figure 7-SAH+Met+DMSO-FITC.tif]

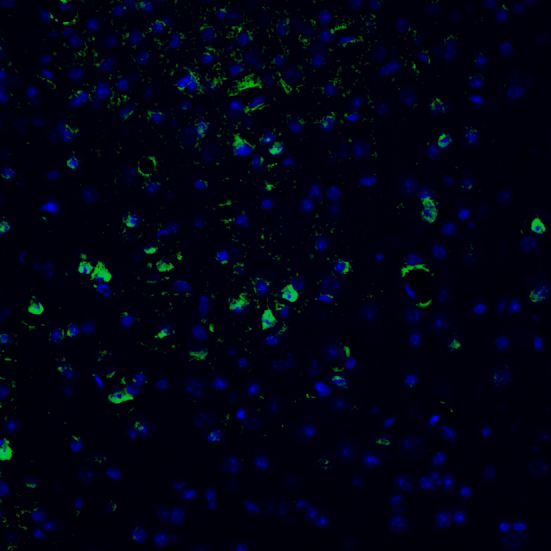

Supplement: Supplementary file 2 [file DataSheet1.ZIP › data sheet for review purpose only (1)/Original date for review purpose only (1)/FJC/Figure 7-SAH+Met+DMSO-merge.tif]

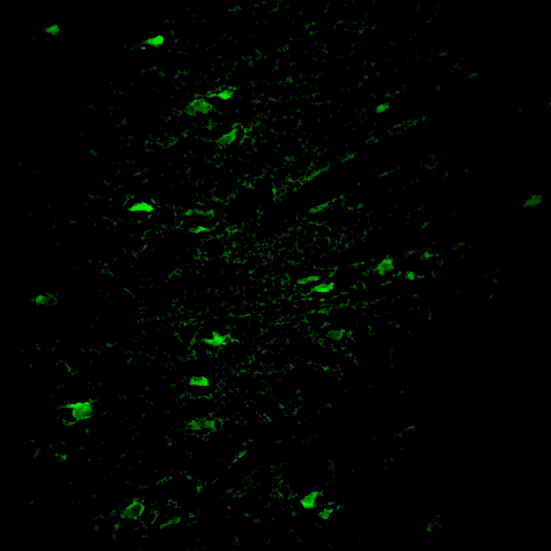

Supplement: Supplementary file 2 [file DataSheet1.ZIP › data sheet for review purpose only (1)/Original date for review purpose only (1)/FJC/Figure 7-SAH+Met-FITC.tif]

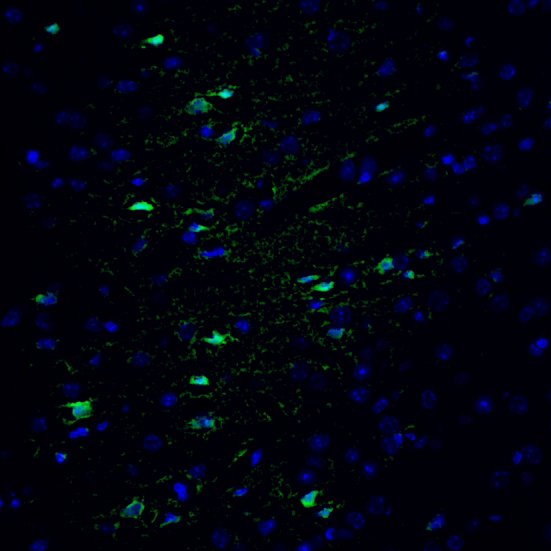

Supplement: Supplementary file 2 [file DataSheet1.ZIP › data sheet for review purpose only (1)/Original date for review purpose only (1)/FJC/Figure 7-SAH+Met-merge.tif]

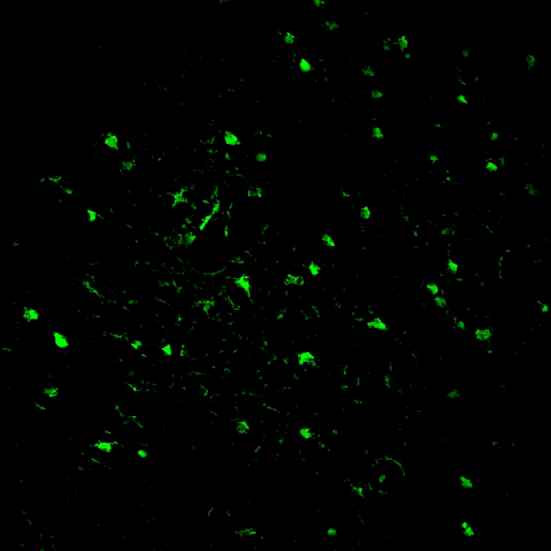

Supplement: Supplementary file 2 [file DataSheet1.ZIP › data sheet for review purpose only (1)/Original date for review purpose only (1)/FJC/Figure 7-SAH+Vehicle-FITC.tif]

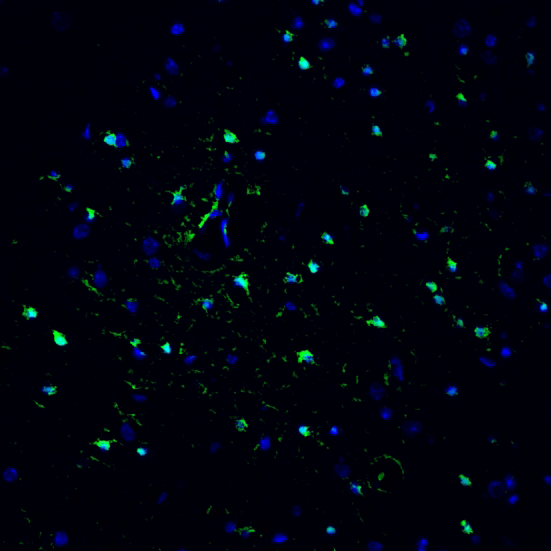

Supplement: Supplementary file 2 [file DataSheet1.ZIP › data sheet for review purpose only (1)/Original date for review purpose only (1)/FJC/Figure 7-SAH+Vehicle-merge.tif]

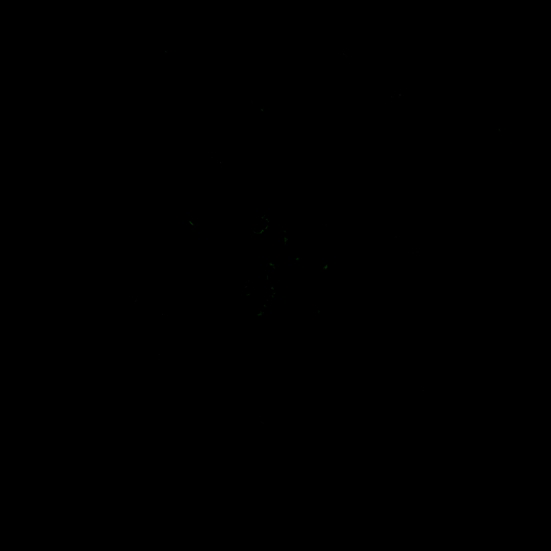

Supplement: Supplementary file 2 [file DataSheet1.ZIP › data sheet for review purpose only (1)/Original date for review purpose only (1)/FJC/Figure 7-Sham-FITC.tif]

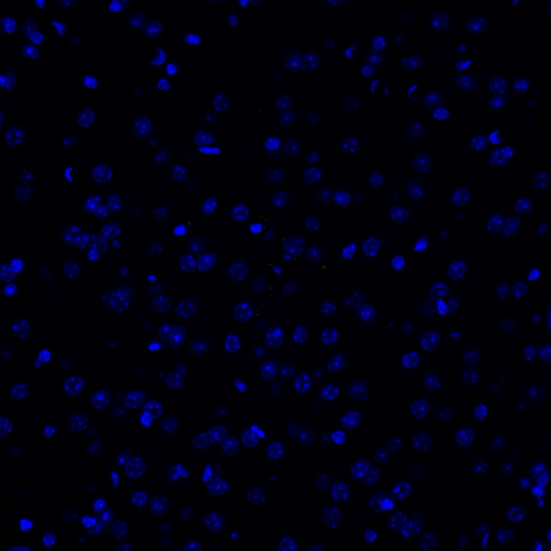

Supplement: Supplementary file 2 [file DataSheet1.ZIP › data sheet for review purpose only (1)/Original date for review purpose only (1)/FJC/Figure 7-Sham-merge.tif]

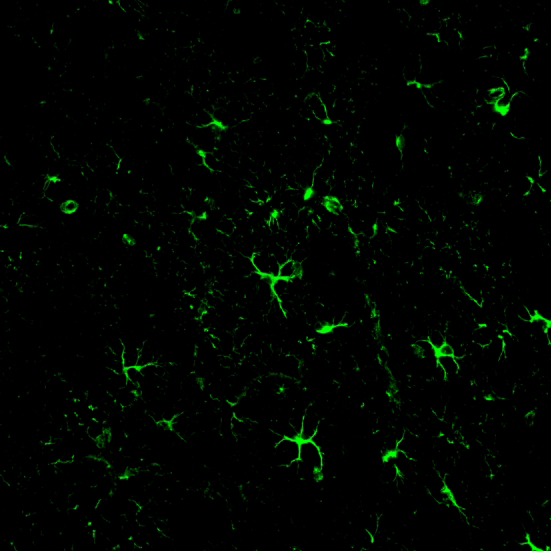

Supplement: Supplementary file 2 [file DataSheet1.ZIP › data sheet for review purpose only (1)/Original date for review purpose only (1)/IF/Figure 3/NLRP3 and GFAP/NLRP3 and GFAP-GFAP.tif]

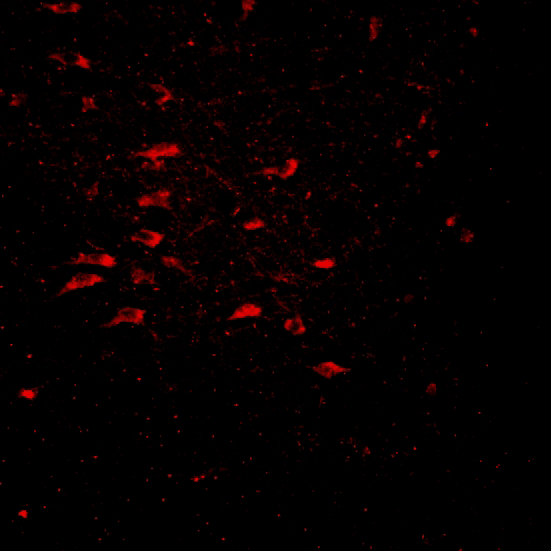

Supplement: Supplementary file 2 [file DataSheet1.ZIP › data sheet for review purpose only (1)/Original date for review purpose only (1)/IF/Figure 3/NLRP3 and GFAP/NLRP3 and GFAP-NLRP3.tif]

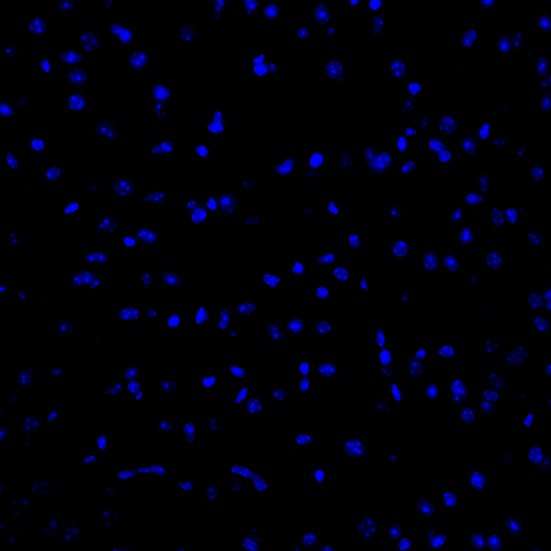

Supplement: Supplementary file 2 [file DataSheet1.ZIP › data sheet for review purpose only (1)/Original date for review purpose only (1)/IF/Figure 3/NLRP3 and GFAP/NLRP3 and GFAP-dapi.tif]

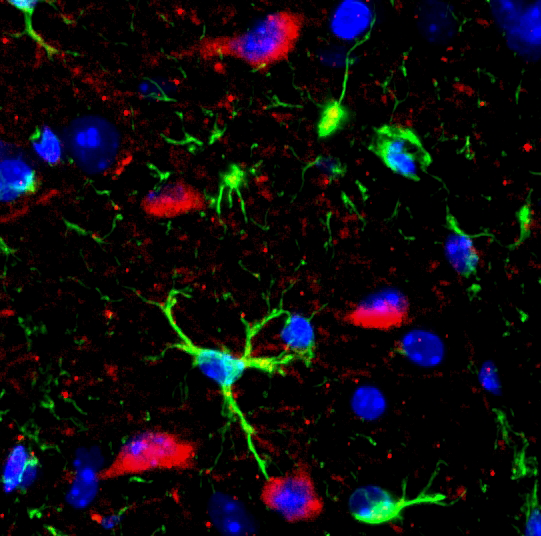

Supplement: Supplementary file 2 [file DataSheet1.ZIP › data sheet for review purpose only (1)/Original date for review purpose only (1)/IF/Figure 3/NLRP3 and GFAP/NLRP3 and GFAP-enlarge.tif]

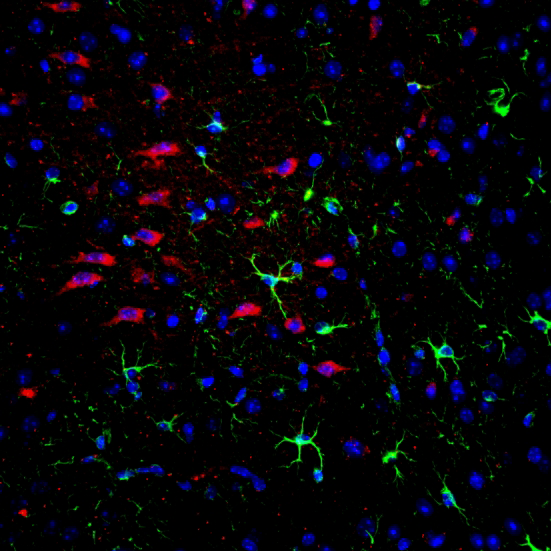

Supplement: Supplementary file 2 [file DataSheet1.ZIP › data sheet for review purpose only (1)/Original date for review purpose only (1)/IF/Figure 3/NLRP3 and GFAP/NLRP3 and GFAP-merge.tif]

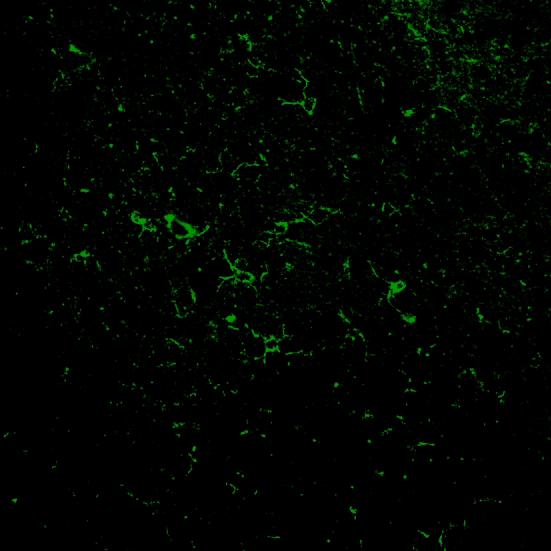

Supplement: Supplementary file 2 [file DataSheet1.ZIP › data sheet for review purpose only (1)/Original date for review purpose only (1)/IF/Figure 3/NLRP3 and IBA1/NLRP3 and IBA-IBA1.tif]

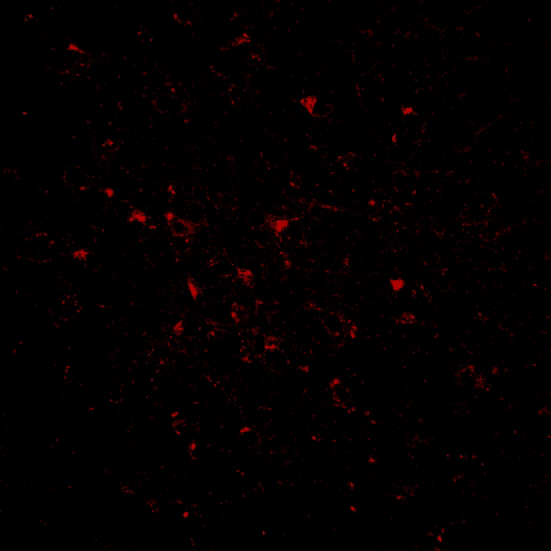

Supplement: Supplementary file 2 [file DataSheet1.ZIP › data sheet for review purpose only (1)/Original date for review purpose only (1)/IF/Figure 3/NLRP3 and IBA1/NLRP3 and IBA-NLRP3.tif]

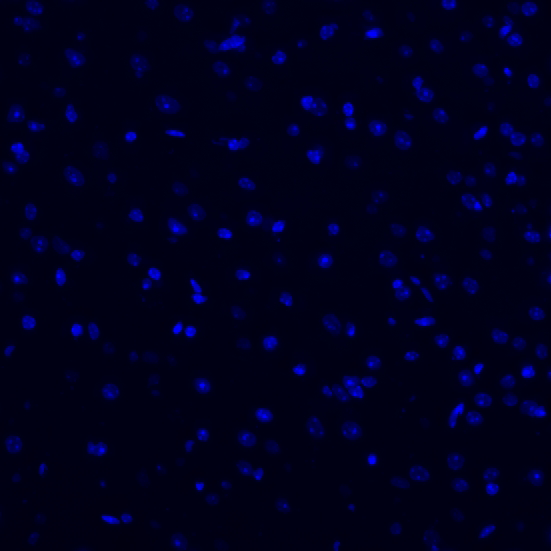

Supplement: Supplementary file 2 [file DataSheet1.ZIP › data sheet for review purpose only (1)/Original date for review purpose only (1)/IF/Figure 3/NLRP3 and IBA1/NLRP3 and IBA-dapi.tif]

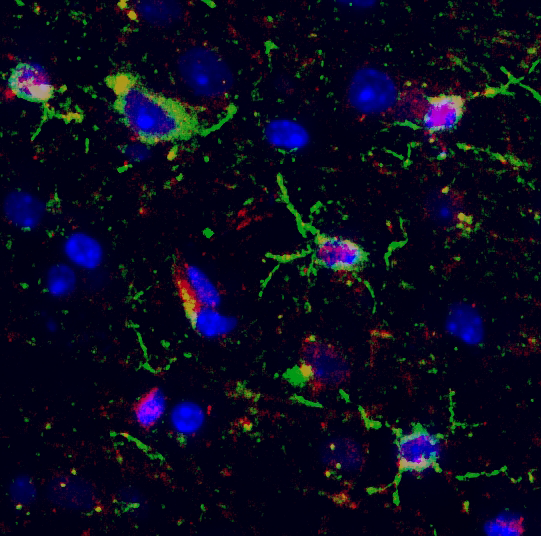

Supplement: Supplementary file 2 [file DataSheet1.ZIP › data sheet for review purpose only (1)/Original date for review purpose only (1)/IF/Figure 3/NLRP3 and IBA1/NLRP3 and IBA-enlarge.tif]

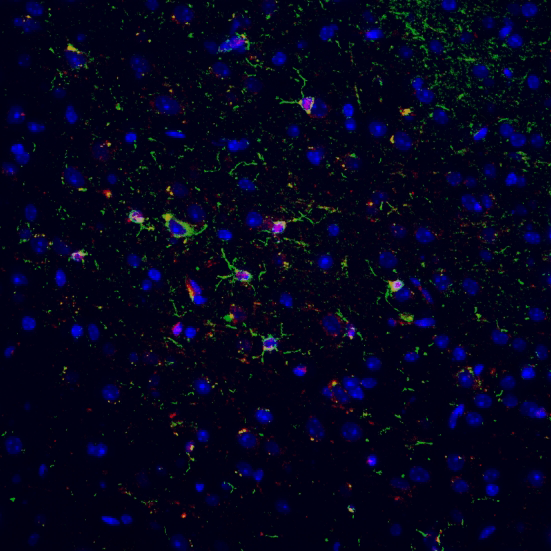

Supplement: Supplementary file 2 [file DataSheet1.ZIP › data sheet for review purpose only (1)/Original date for review purpose only (1)/IF/Figure 3/NLRP3 and IBA1/NLRP3 and IBA-merge.tif]

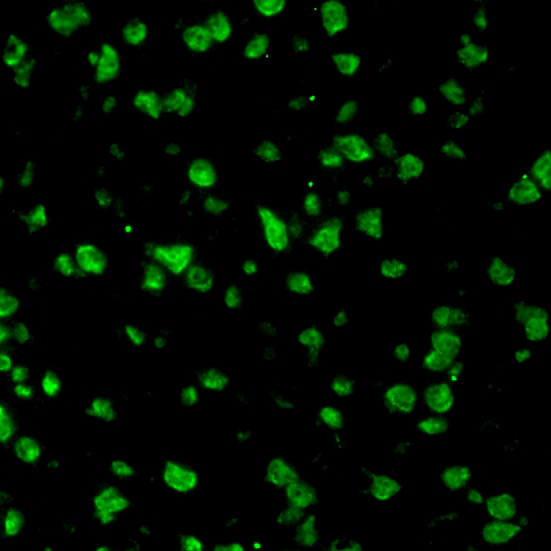

Supplement: Supplementary file 2 [file DataSheet1.ZIP › data sheet for review purpose only (1)/Original date for review purpose only (1)/IF/Figure 3/NLRP3 and NEUN/NLRP3 and NEUN-NEUN.tif]

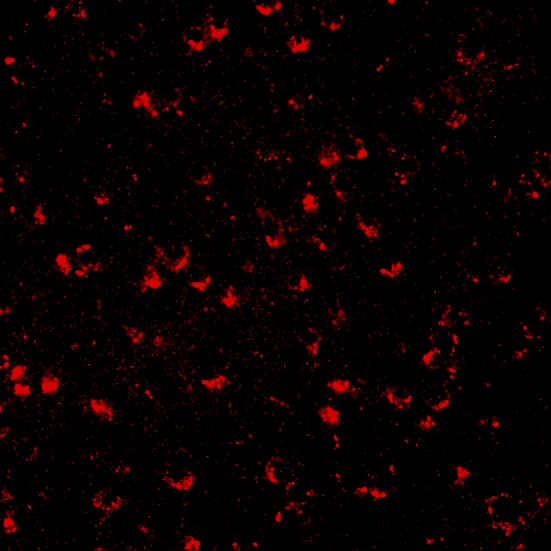

Supplement: Supplementary file 2 [file DataSheet1.ZIP › data sheet for review purpose only (1)/Original date for review purpose only (1)/IF/Figure 3/NLRP3 and NEUN/NLRP3 and NEUN-NLRP3.tif]

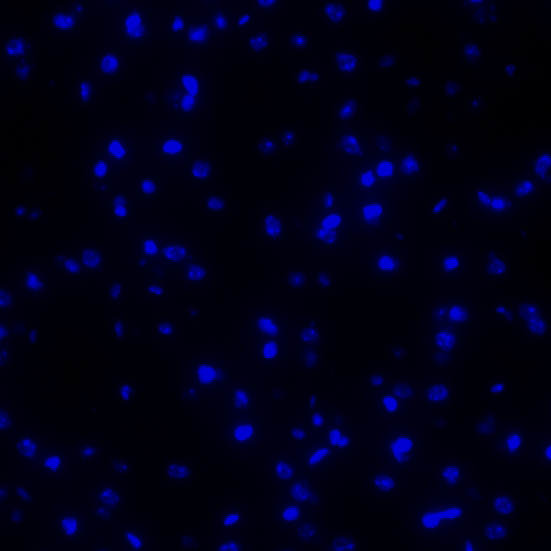

Supplement: Supplementary file 2 [file DataSheet1.ZIP › data sheet for review purpose only (1)/Original date for review purpose only (1)/IF/Figure 3/NLRP3 and NEUN/NLRP3 and NEUN-dapi.tif]

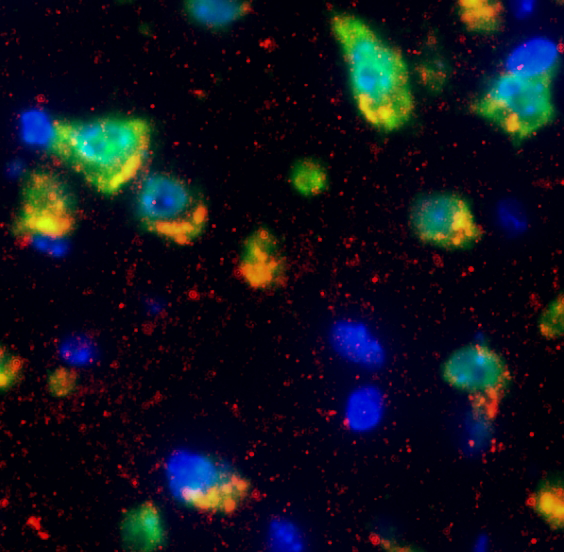

Supplement: Supplementary file 2 [file DataSheet1.ZIP › data sheet for review purpose only (1)/Original date for review purpose only (1)/IF/Figure 3/NLRP3 and NEUN/NLRP3 and NEUN-enlarge.tif]

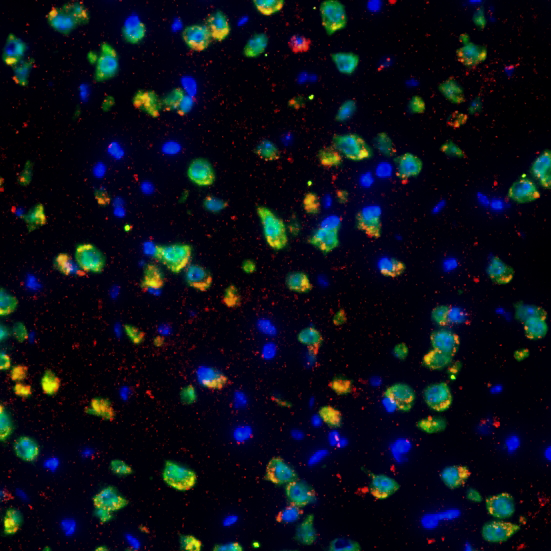

Supplement: Supplementary file 2 [file DataSheet1.ZIP › data sheet for review purpose only (1)/Original date for review purpose only (1)/IF/Figure 3/NLRP3 and NEUN/NLRP3 and NEUN-merge.tif]

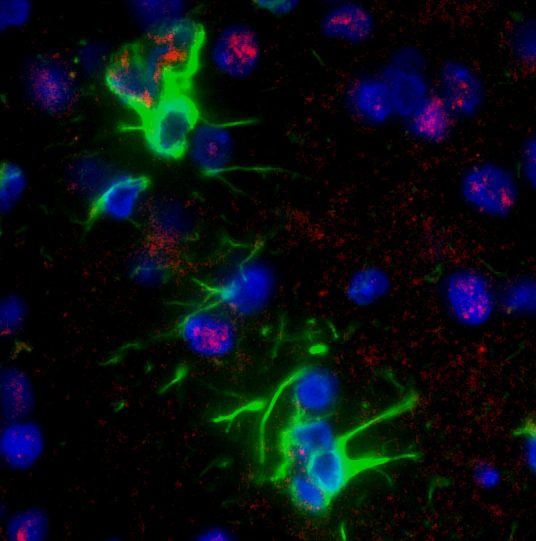

Supplement: Supplementary file 2 [file DataSheet1.ZIP › data sheet for review purpose only (1)/Original date for review purpose only (1)/IF/Figure 3/pAMPK and GFAP/PAMPK and GFAP-enlarge.tif]

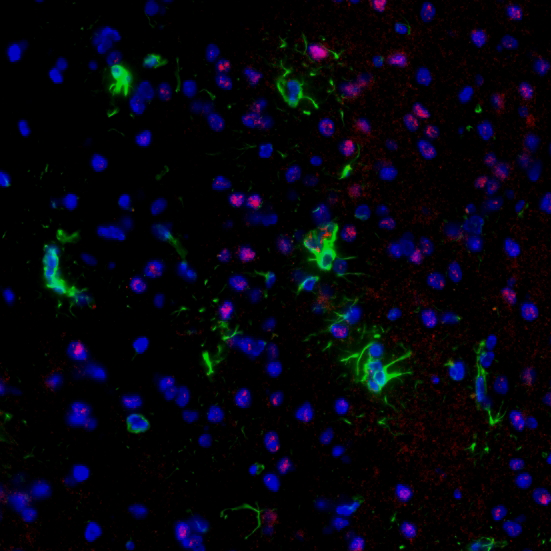

Supplement: Supplementary file 2 [file DataSheet1.ZIP › data sheet for review purpose only (1)/Original date for review purpose only (1)/IF/Figure 3/pAMPK and GFAP/PAMPK and GFAP-merge.tif]

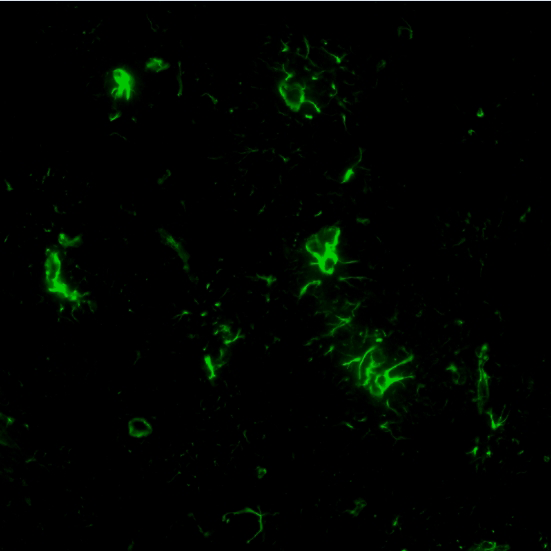

Supplement: Supplementary file 2 [file DataSheet1.ZIP › data sheet for review purpose only (1)/Original date for review purpose only (1)/IF/Figure 3/pAMPK and GFAP/pAMPK and GFAP-GFAP.tif]

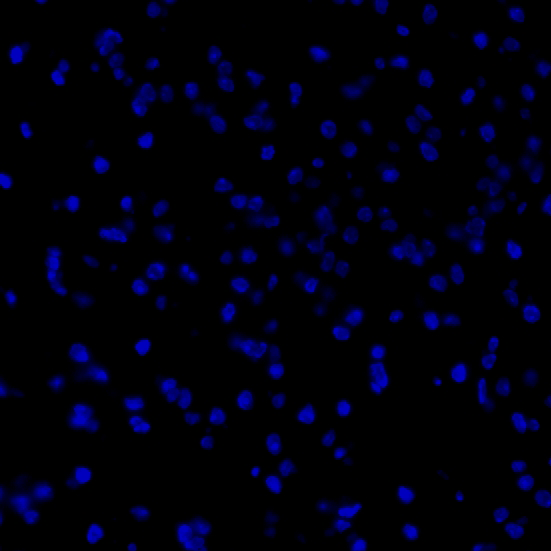

Supplement: Supplementary file 2 [file DataSheet1.ZIP › data sheet for review purpose only (1)/Original date for review purpose only (1)/IF/Figure 3/pAMPK and GFAP/pAMPK and GFAP-dapi.tif]

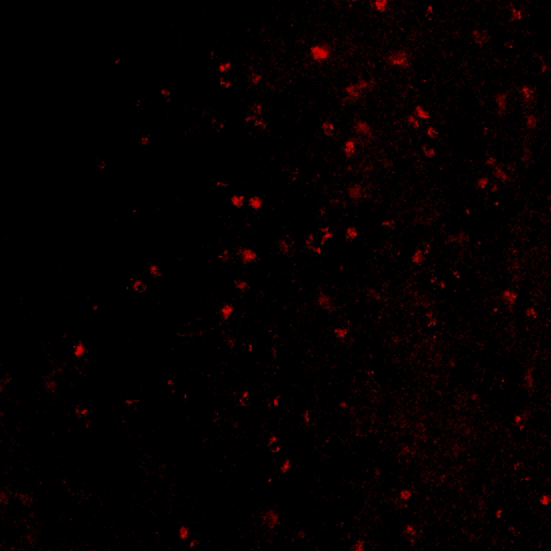

Supplement: Supplementary file 2 [file DataSheet1.ZIP › data sheet for review purpose only (1)/Original date for review purpose only (1)/IF/Figure 3/pAMPK and GFAP/pAMPK and GFAP-pAMPK.tif]

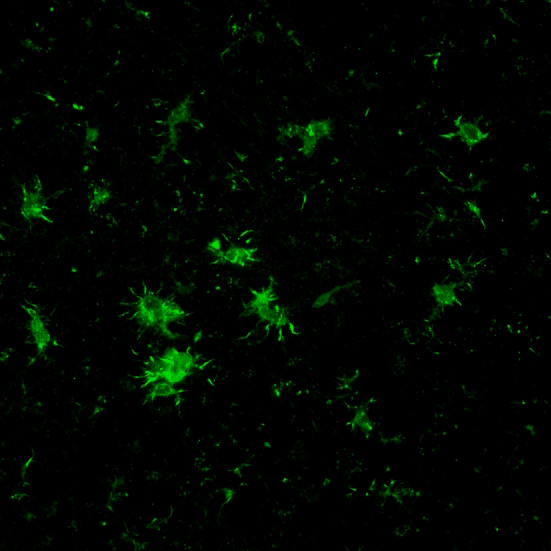

Supplement: Supplementary file 2 [file DataSheet1.ZIP › data sheet for review purpose only (1)/Original date for review purpose only (1)/IF/Figure 3/pAMPK and IBA1/pAMPK and IBA1-IBA1.tif]

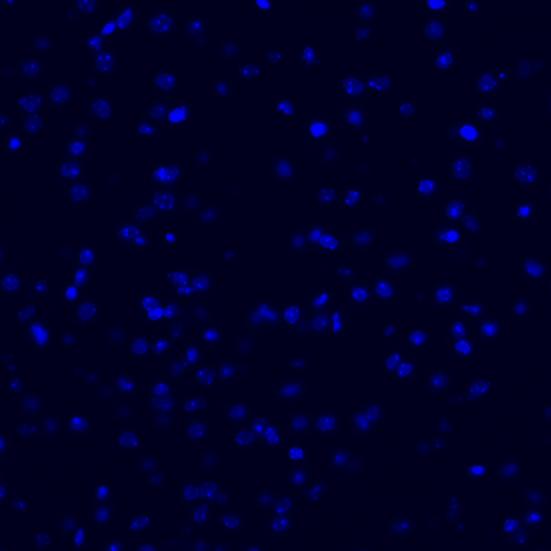

Supplement: Supplementary file 2 [file DataSheet1.ZIP › data sheet for review purpose only (1)/Original date for review purpose only (1)/IF/Figure 3/pAMPK and IBA1/pAMPK and IBA1-dapi.tif]

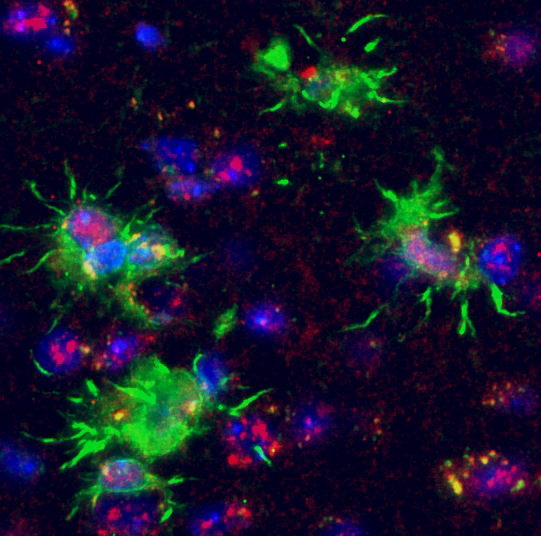

Supplement: Supplementary file 2 [file DataSheet1.ZIP › data sheet for review purpose only (1)/Original date for review purpose only (1)/IF/Figure 3/pAMPK and IBA1/pAMPK and IBA1-enlarge.tif]

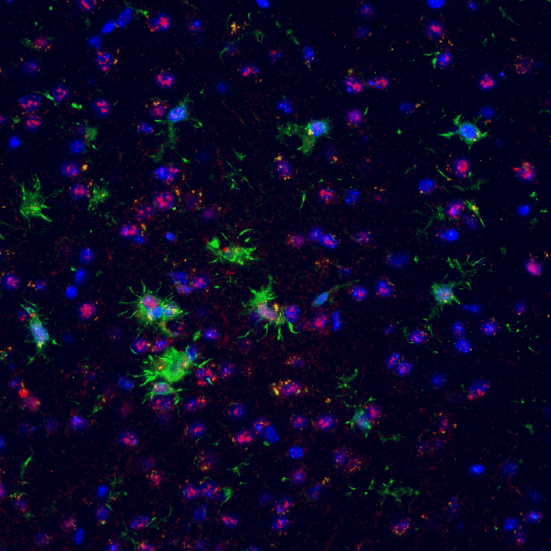

Supplement: Supplementary file 2 [file DataSheet1.ZIP › data sheet for review purpose only (1)/Original date for review purpose only (1)/IF/Figure 3/pAMPK and IBA1/pAMPK and IBA1-merge.tif]

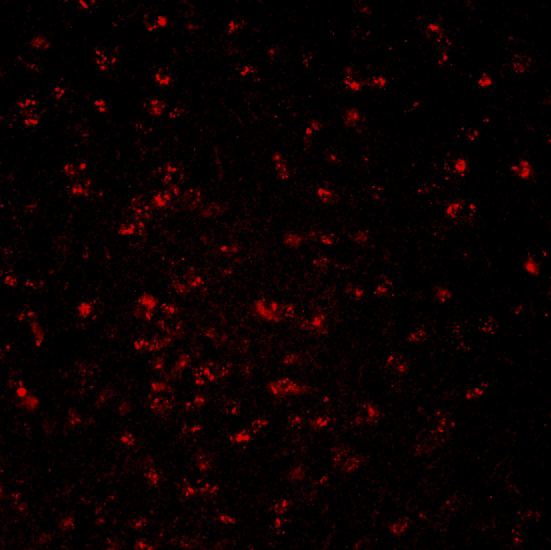

Supplement: Supplementary file 2 [file DataSheet1.ZIP › data sheet for review purpose only (1)/Original date for review purpose only (1)/IF/Figure 3/pAMPK and IBA1/pAMPK and IBA1-pAMPK.tif]

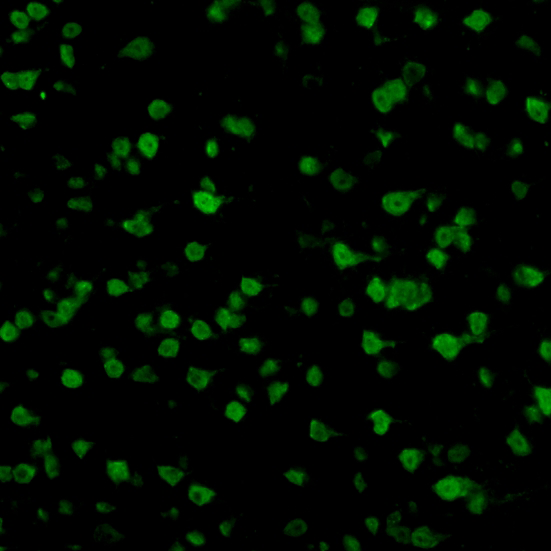

Supplement: Supplementary file 2 [file DataSheet1.ZIP › data sheet for review purpose only (1)/Original date for review purpose only (1)/IF/Figure 3/pAMPK and NEUN/pAMPK and NEUN-NEUN.tif]

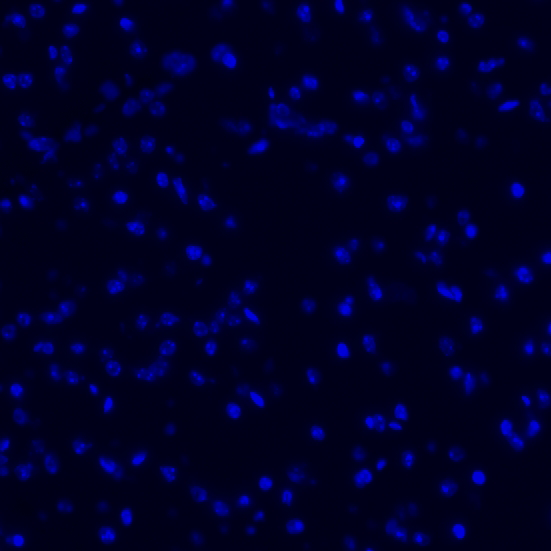

Supplement: Supplementary file 2 [file DataSheet1.ZIP › data sheet for review purpose only (1)/Original date for review purpose only (1)/IF/Figure 3/pAMPK and NEUN/pAMPK and NEUN-dapi.tif]

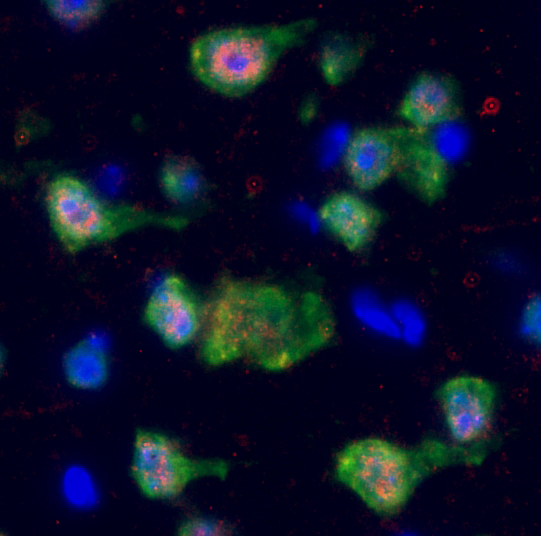

Supplement: Supplementary file 2 [file DataSheet1.ZIP › data sheet for review purpose only (1)/Original date for review purpose only (1)/IF/Figure 3/pAMPK and NEUN/pAMPK and NEUN-pAMPK-enlarge.tif]

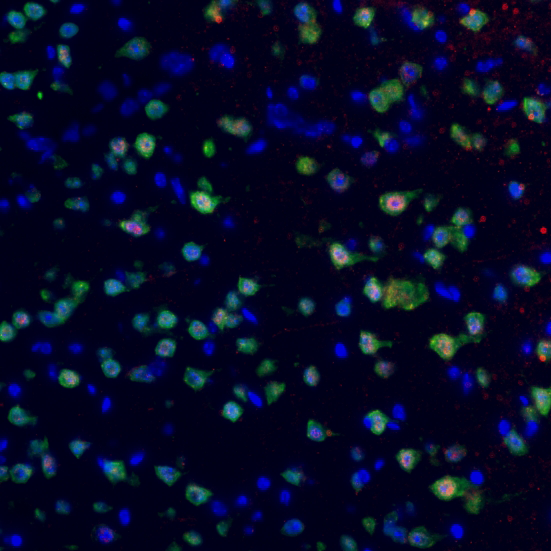

Supplement: Supplementary file 2 [file DataSheet1.ZIP › data sheet for review purpose only (1)/Original date for review purpose only (1)/IF/Figure 3/pAMPK and NEUN/pAMPK and NEUN-pAMPK-merge.tif]

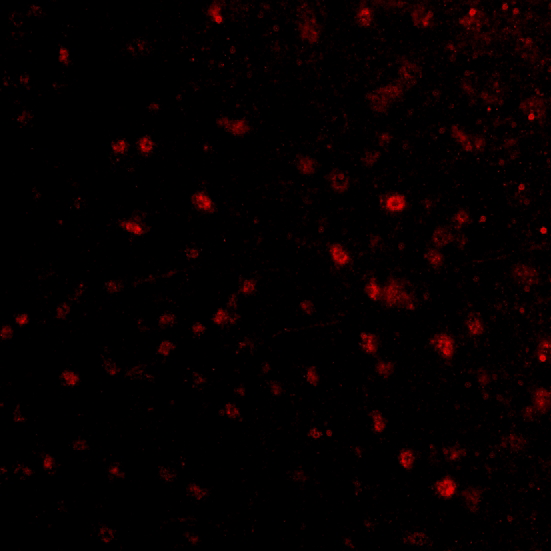

Supplement: Supplementary file 2 [file DataSheet1.ZIP › data sheet for review purpose only (1)/Original date for review purpose only (1)/IF/Figure 3/pAMPK and NEUN/pAMPK and NEUN-pAMPK.tif]

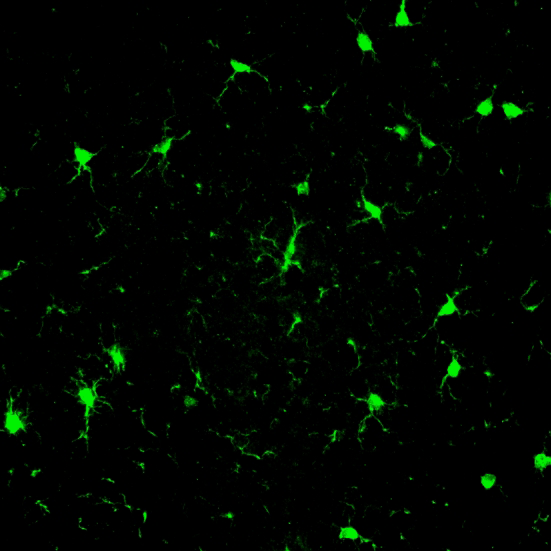

Supplement: Supplementary file 2 [file DataSheet1.ZIP › data sheet for review purpose only (1)/Original date for review purpose only (1)/IF/Figure 8/Figure 8 SAH+MET+CC-FITC.tif]

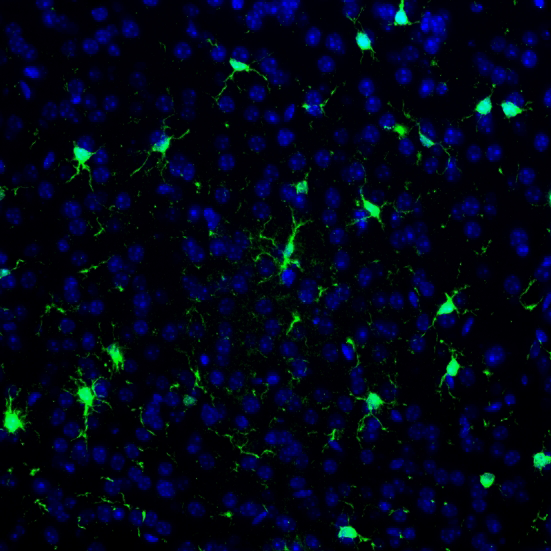

Supplement: Supplementary file 2 [file DataSheet1.ZIP › data sheet for review purpose only (1)/Original date for review purpose only (1)/IF/Figure 8/Figure 8 SAH+Met+CC-merge.tif]

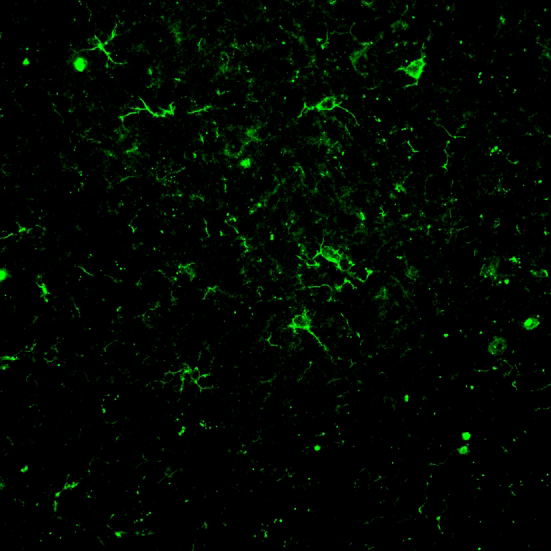

Supplement: Supplementary file 2 [file DataSheet1.ZIP › data sheet for review purpose only (1)/Original date for review purpose only (1)/IF/Figure 8/Figure 8 SAH+Met+DMSO-FITC.tif]

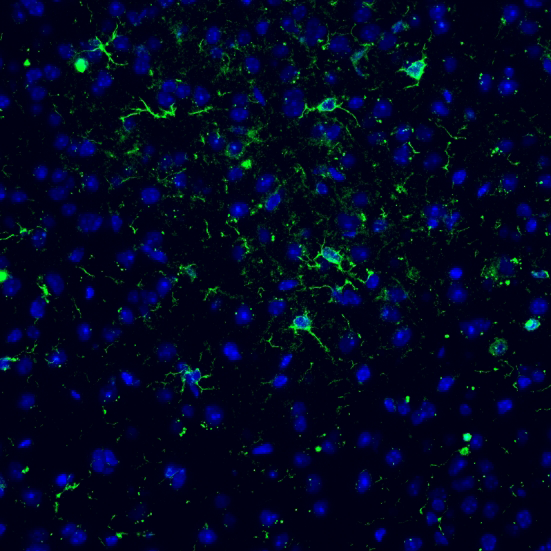

Supplement: Supplementary file 2 [file DataSheet1.ZIP › data sheet for review purpose only (1)/Original date for review purpose only (1)/IF/Figure 8/Figure 8 SAH+Met+DMSO-merge.tif]

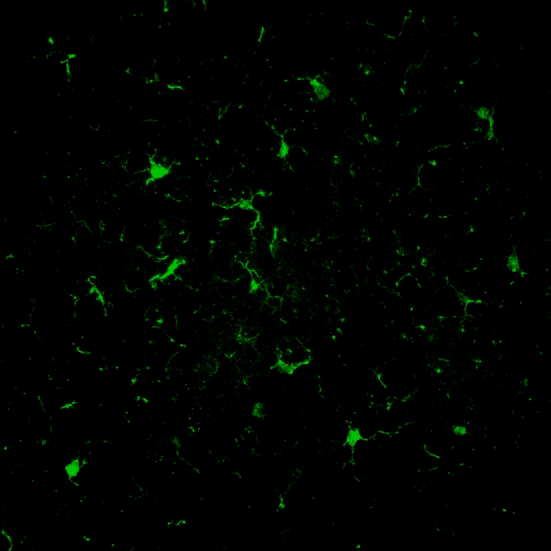

Supplement: Supplementary file 2 [file DataSheet1.ZIP › data sheet for review purpose only (1)/Original date for review purpose only (1)/IF/Figure 8/Figure 8 SAH+Met-FITC.tif]

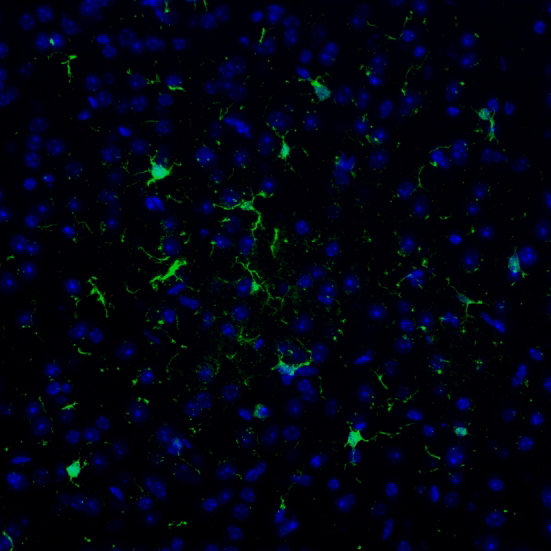

Supplement: Supplementary file 2 [file DataSheet1.ZIP › data sheet for review purpose only (1)/Original date for review purpose only (1)/IF/Figure 8/Figure 8 SAH+Met-merge.tif]

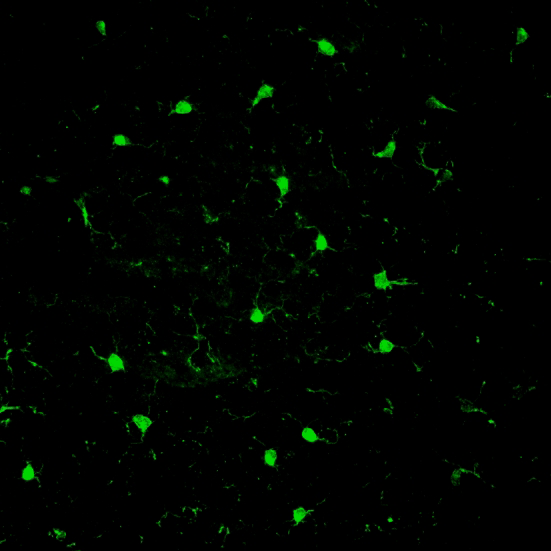

Supplement: Supplementary file 2 [file DataSheet1.ZIP › data sheet for review purpose only (1)/Original date for review purpose only (1)/IF/Figure 8/Figure 8 SAH+Vehicle-FITC.tif]

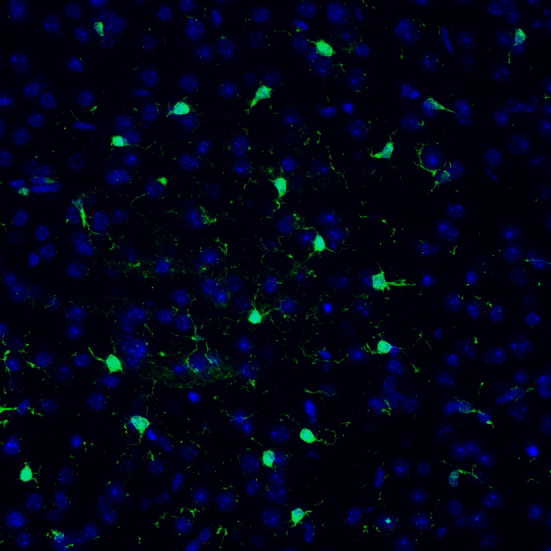

Supplement: Supplementary file 2 [file DataSheet1.ZIP › data sheet for review purpose only (1)/Original date for review purpose only (1)/IF/Figure 8/Figure 8 SAH+Vehicle-merge.tif]

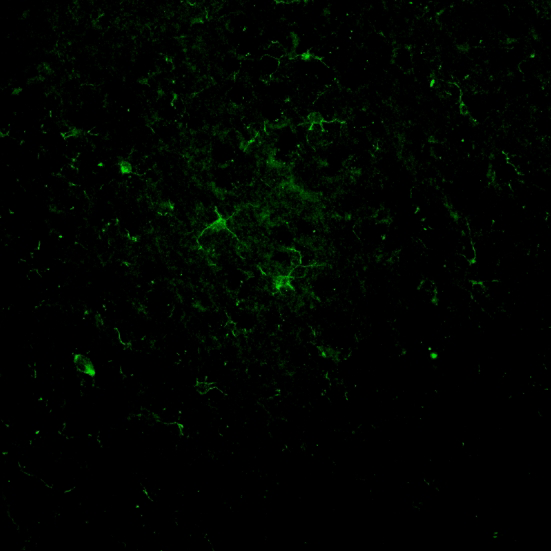

Supplement: Supplementary file 2 [file DataSheet1.ZIP › data sheet for review purpose only (1)/Original date for review purpose only (1)/IF/Figure 8/Figure 8 Sham-FITC.tif]

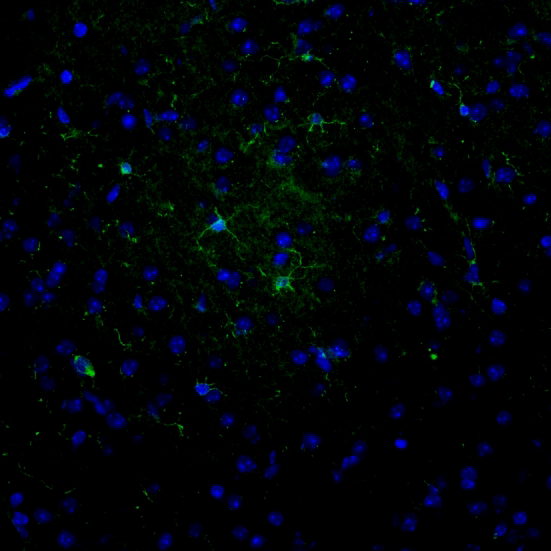

Supplement: Supplementary file 2 [file DataSheet1.ZIP › data sheet for review purpose only (1)/Original date for review purpose only (1)/IF/Figure 8/Figure 8 Sham-merge.tif]

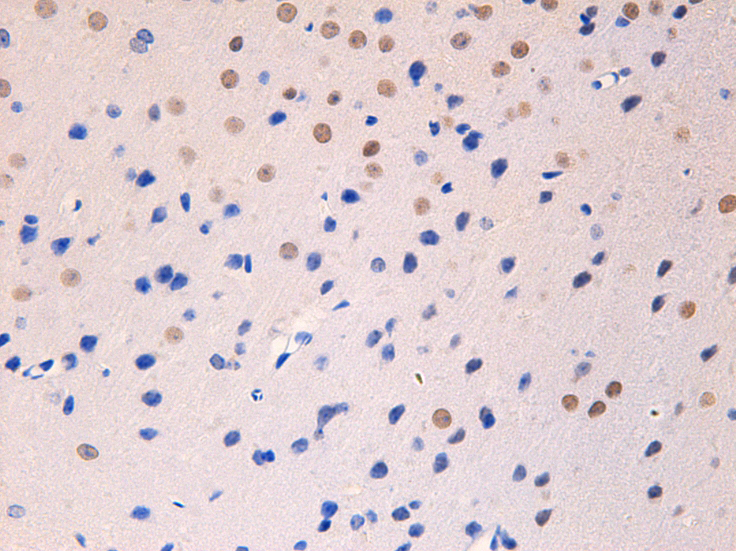

Supplement: Supplementary file 2 [file DataSheet1.ZIP › data sheet for review purpose only (1)/Original date for review purpose only (1)/IHC/Figure 6-NLRP3/NLRP3-SAH+Metformin.tif]

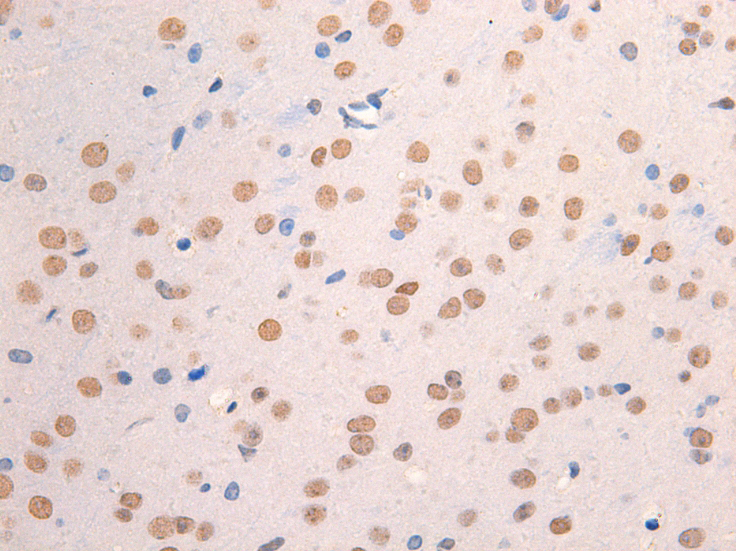

Supplement: Supplementary file 2 [file DataSheet1.ZIP › data sheet for review purpose only (1)/Original date for review purpose only (1)/IHC/Figure 6-NLRP3/NLRP3-SAH+Vehicle.tif]

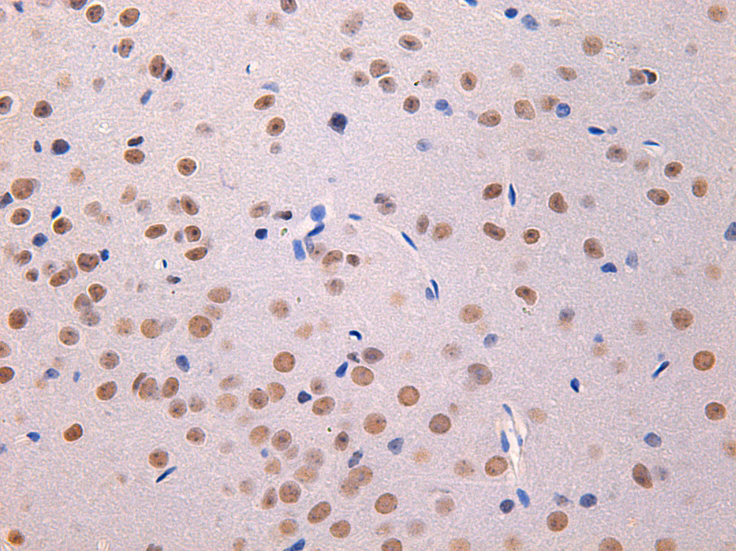

Supplement: Supplementary file 2 [file DataSheet1.ZIP › data sheet for review purpose only (1)/Original date for review purpose only (1)/IHC/Figure 6-NLRP3/NLRP3-SAH.tif]

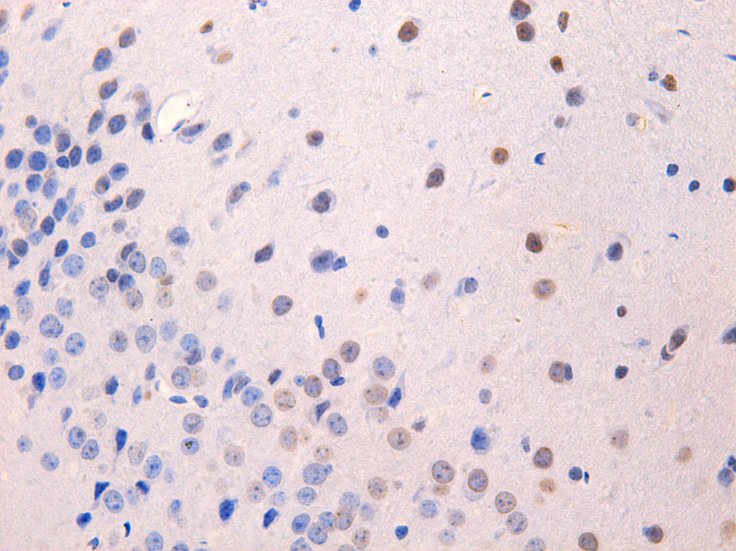

Supplement: Supplementary file 2 [file DataSheet1.ZIP › data sheet for review purpose only (1)/Original date for review purpose only (1)/IHC/Figure 6-NLRP3/NLRP3-Sham.tif]

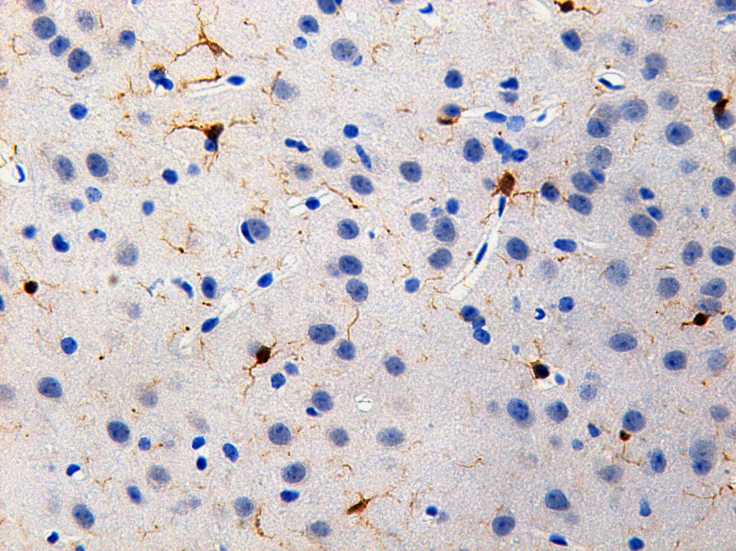

Supplement: Supplementary file 2 [file DataSheet1.ZIP › data sheet for review purpose only (1)/Original date for review purpose only (1)/IHC/Figure 8-IBA1/IBA1-SAH+Met+CC.tif]

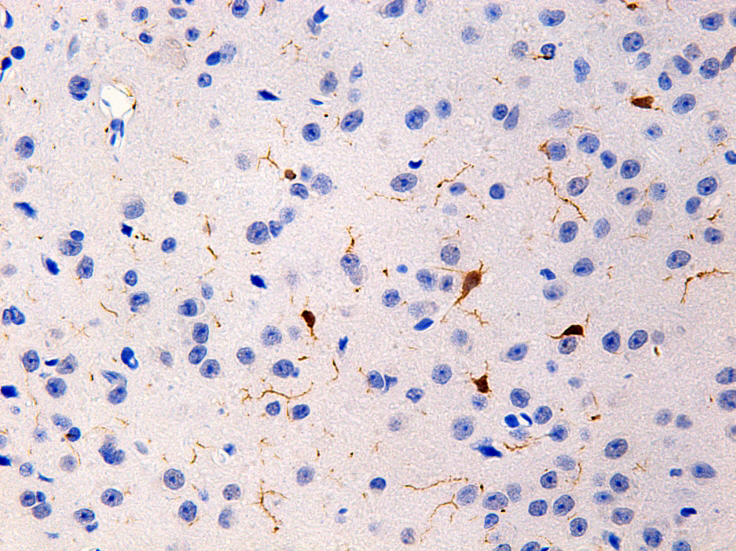

Supplement: Supplementary file 2 [file DataSheet1.ZIP › data sheet for review purpose only (1)/Original date for review purpose only (1)/IHC/Figure 8-IBA1/IBA1-SAH+Met+DMSO.tif]

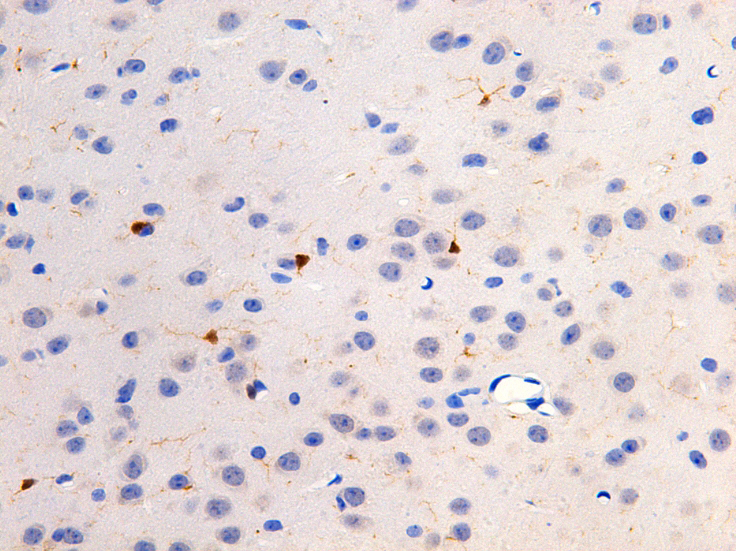

Supplement: Supplementary file 2 [file DataSheet1.ZIP › data sheet for review purpose only (1)/Original date for review purpose only (1)/IHC/Figure 8-IBA1/IBA1-SAH+Met.tif]

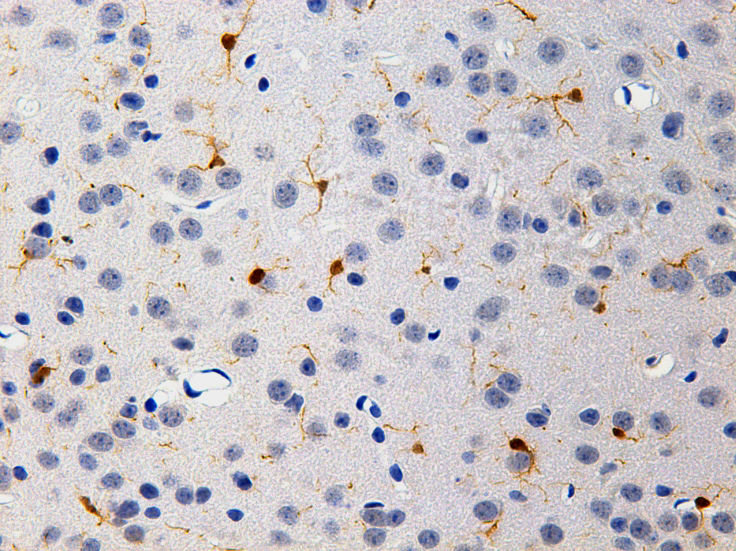

Supplement: Supplementary file 2 [file DataSheet1.ZIP › data sheet for review purpose only (1)/Original date for review purpose only (1)/IHC/Figure 8-IBA1/IBA1-SAH+Vehicle.tif]

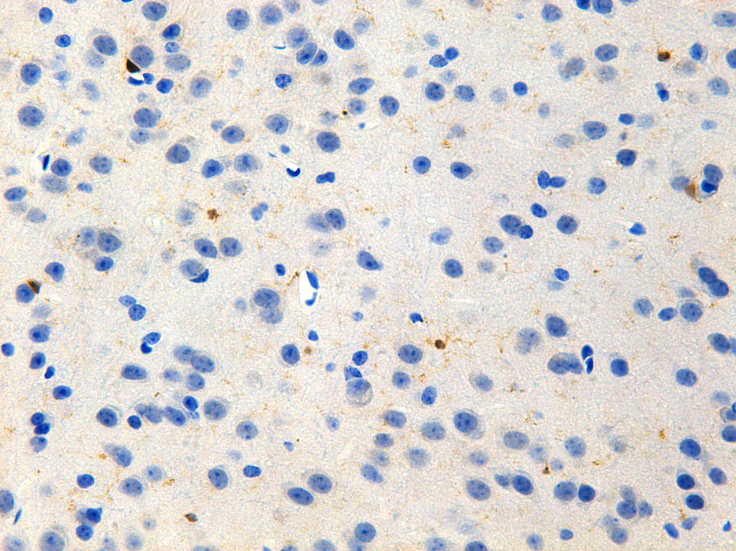

Supplement: Supplementary file 2 [file DataSheet1.ZIP › data sheet for review purpose only (1)/Original date for review purpose only (1)/IHC/Figure 8-IBA1/IBA1-Sham.tif]

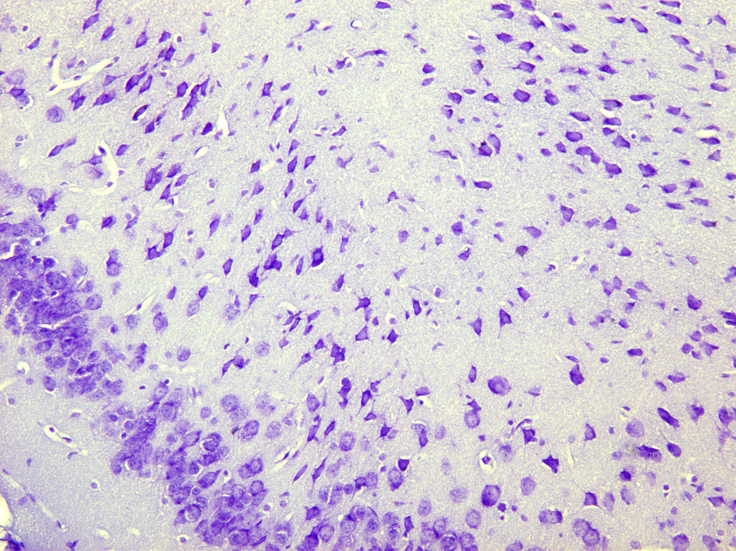

Supplement: Supplementary file 2 [file DataSheet1.ZIP › data sheet for review purpose only (1)/Original date for review purpose only (1)/Nissl/Figure 5-SAH+Metformin.tif]

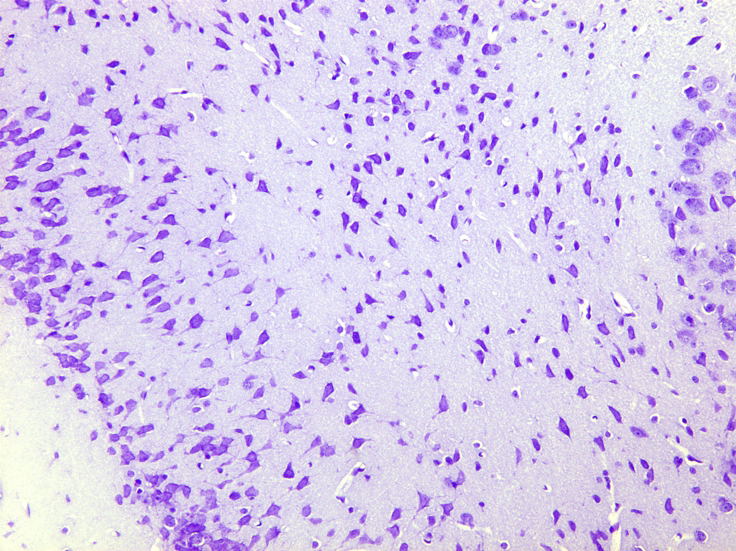

Supplement: Supplementary file 2 [file DataSheet1.ZIP › data sheet for review purpose only (1)/Original date for review purpose only (1)/Nissl/Figure 5-SAH+Vehicle.tif]

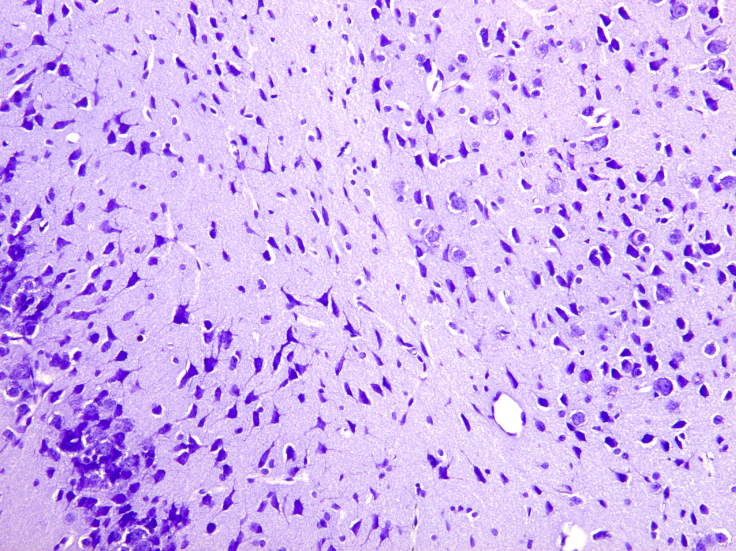

Supplement: Supplementary file 2 [file DataSheet1.ZIP › data sheet for review purpose only (1)/Original date for review purpose only (1)/Nissl/Figure 5-SAH.tif]

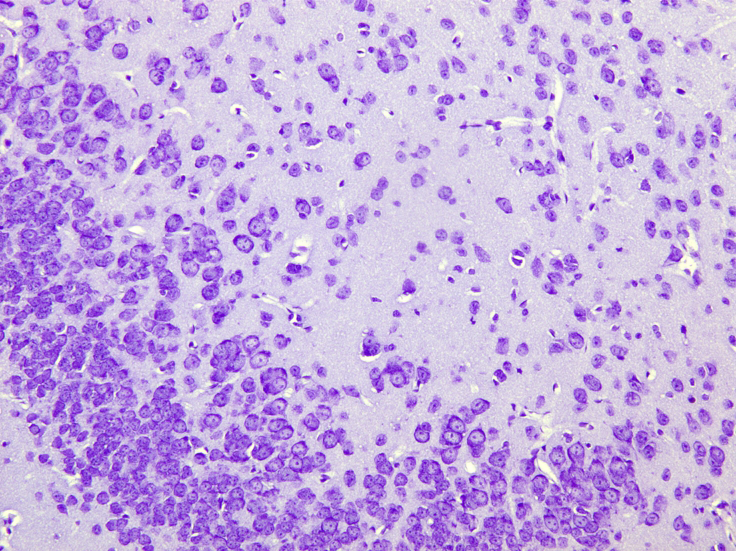

Supplement: Supplementary file 2 [file DataSheet1.ZIP › data sheet for review purpose only (1)/Original date for review purpose only (1)/Nissl/Figure 5-Sham.tif]

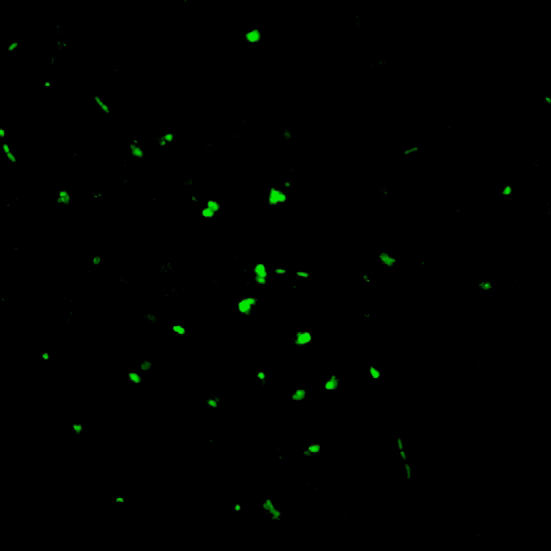

Supplement: Supplementary file 2 [file DataSheet1.ZIP › data sheet for review purpose only (1)/Original date for review purpose only (1)/TUNEL/Figure 5-SAH+Met-FITC.tif]

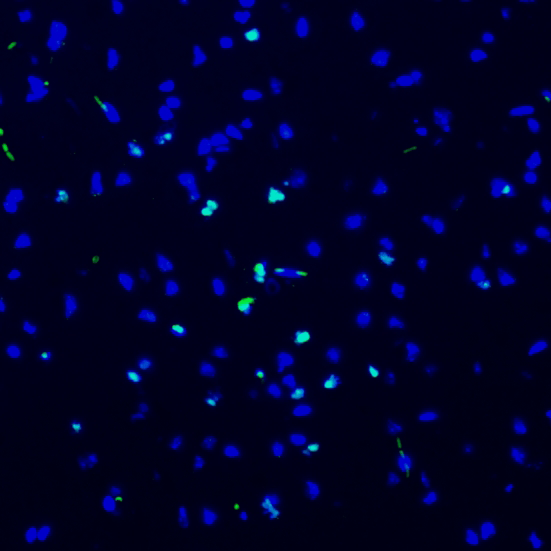

Supplement: Supplementary file 2 [file DataSheet1.ZIP › data sheet for review purpose only (1)/Original date for review purpose only (1)/TUNEL/Figure 5-SAH+Met-merge.tif]

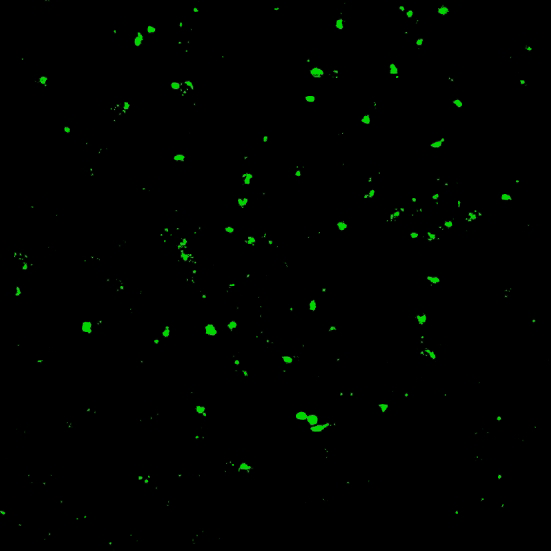

Supplement: Supplementary file 2 [file DataSheet1.ZIP › data sheet for review purpose only (1)/Original date for review purpose only (1)/TUNEL/Figure 5-SAH+Vehicle-FITC.tif]

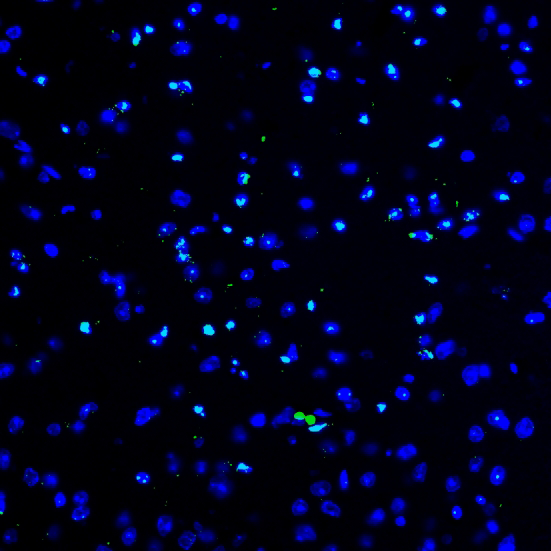

Supplement: Supplementary file 2 [file DataSheet1.ZIP › data sheet for review purpose only (1)/Original date for review purpose only (1)/TUNEL/Figure 5-SAH+Vehicle-merge.tif]

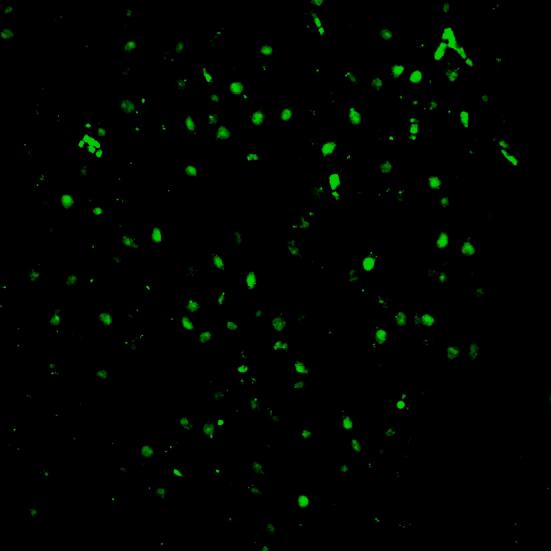

Supplement: Supplementary file 2 [file DataSheet1.ZIP › data sheet for review purpose only (1)/Original date for review purpose only (1)/TUNEL/Figure 5-SAH-FITC.tif]

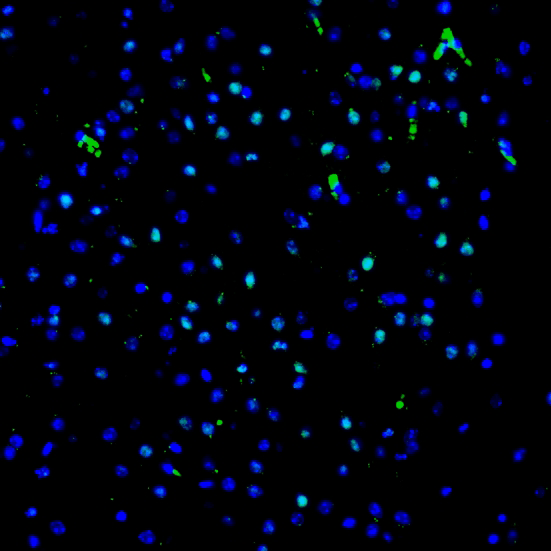

Supplement: Supplementary file 2 [file DataSheet1.ZIP › data sheet for review purpose only (1)/Original date for review purpose only (1)/TUNEL/Figure 5-SAH-merge.tif]

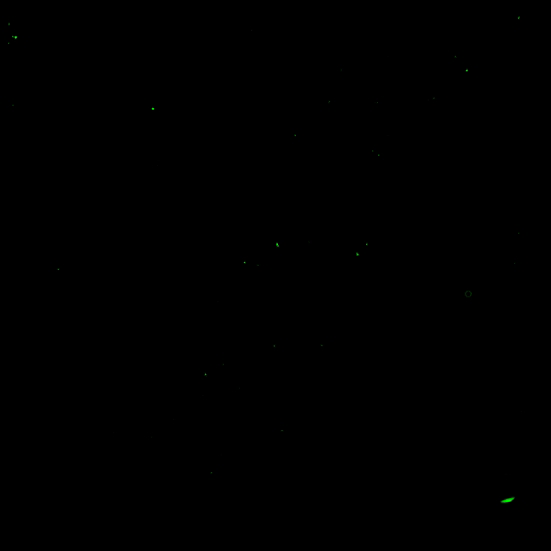

Supplement: Supplementary file 2 [file DataSheet1.ZIP › data sheet for review purpose only (1)/Original date for review purpose only (1)/TUNEL/Figure 5-Sham-FITC.tif]

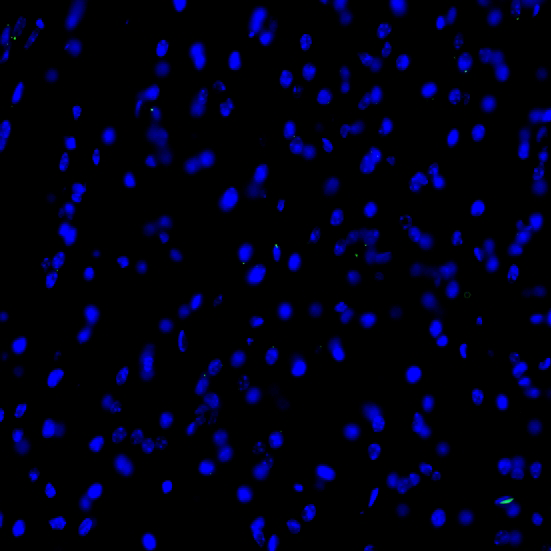

Supplement: Supplementary file 2 [file DataSheet1.ZIP › data sheet for review purpose only (1)/Original date for review purpose only (1)/TUNEL/Figure 5-Sham-merge.tif]

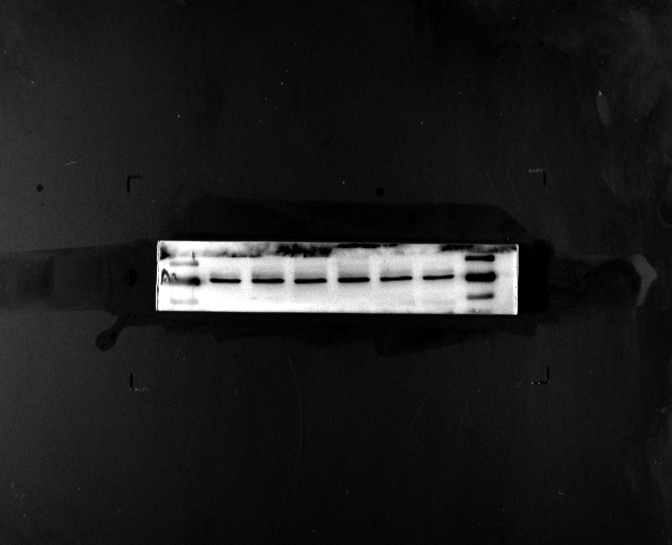

Supplement: Supplementary file 2 [file DataSheet1.ZIP › data sheet for review purpose only (1)/Original date for review purpose only (1)/western blot/Figure 2/Figure 2-AMPK-backgroud.tif]

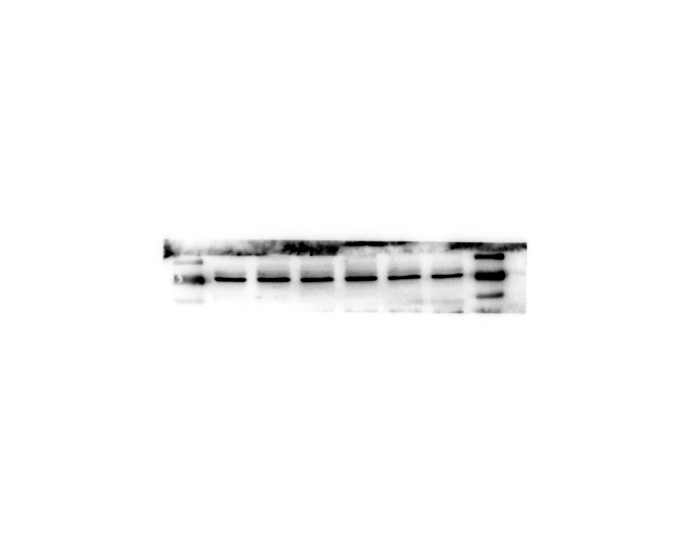

Supplement: Supplementary file 2 [file DataSheet1.ZIP › data sheet for review purpose only (1)/Original date for review purpose only (1)/western blot/Figure 2/Figure 2-AMPK.tif]

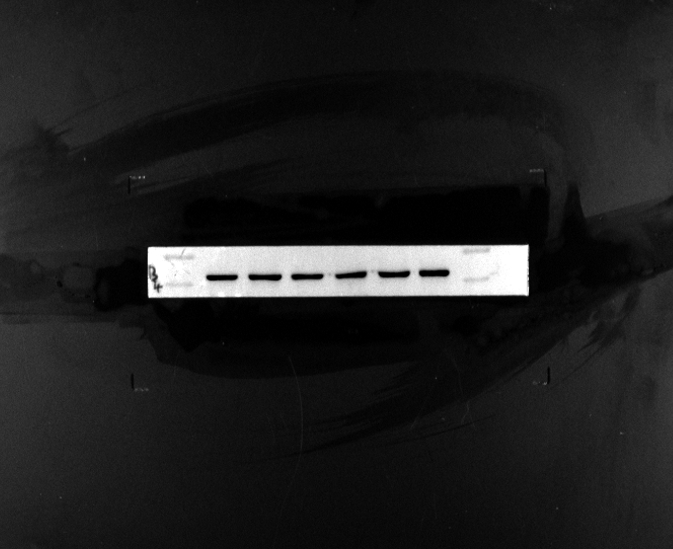

Supplement: Supplementary file 2 [file DataSheet1.ZIP › data sheet for review purpose only (1)/Original date for review purpose only (1)/western blot/Figure 2/Figure 2-B-actin-backgroud.tif]

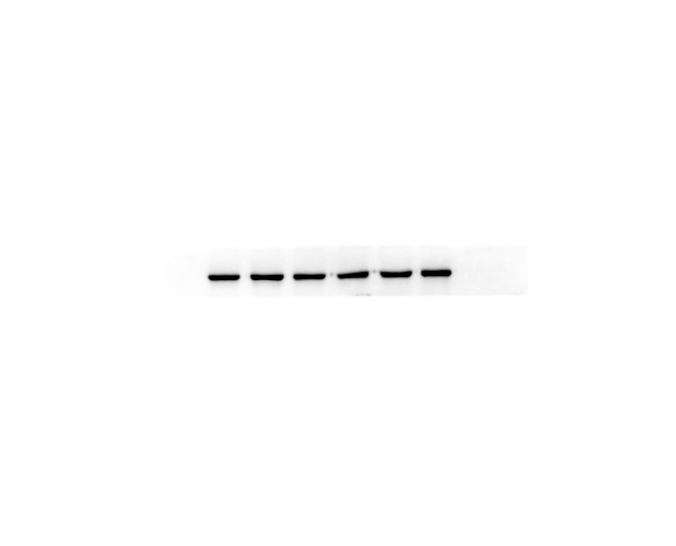

Supplement: Supplementary file 2 [file DataSheet1.ZIP › data sheet for review purpose only (1)/Original date for review purpose only (1)/western blot/Figure 2/Figure 2-B-actin.tif]

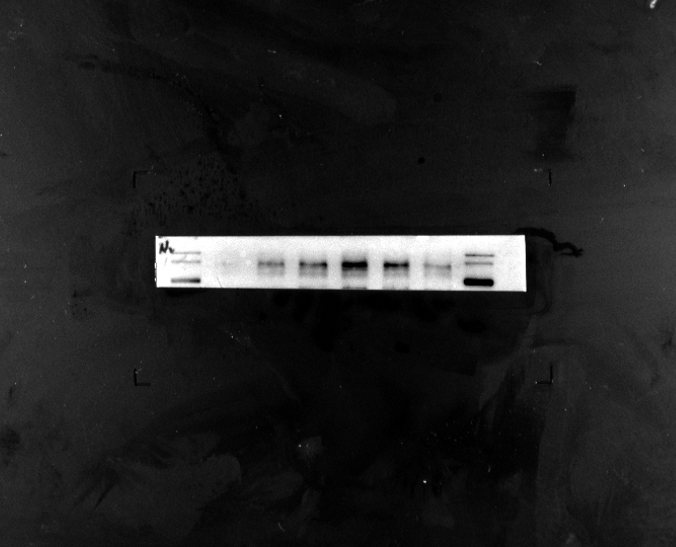

Supplement: Supplementary file 2 [file DataSheet1.ZIP › data sheet for review purpose only (1)/Original date for review purpose only (1)/western blot/Figure 2/Figure 2-NLRP3-backgroud.tif]

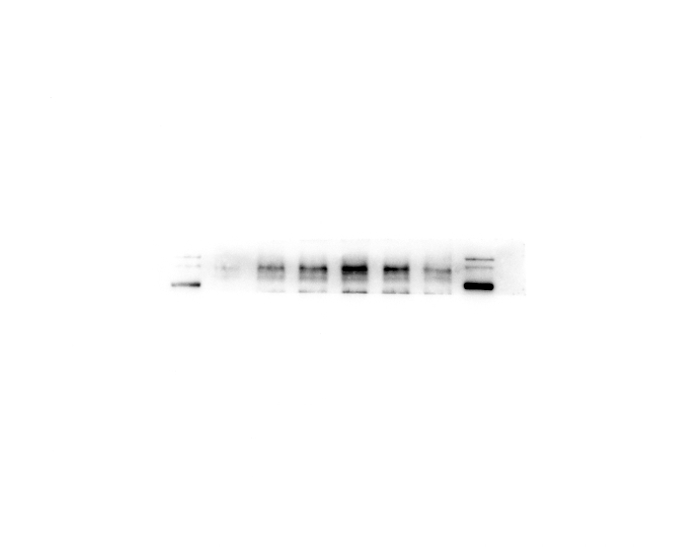

Supplement: Supplementary file 2 [file DataSheet1.ZIP › data sheet for review purpose only (1)/Original date for review purpose only (1)/western blot/Figure 2/Figure 2-NLRP3.tif]

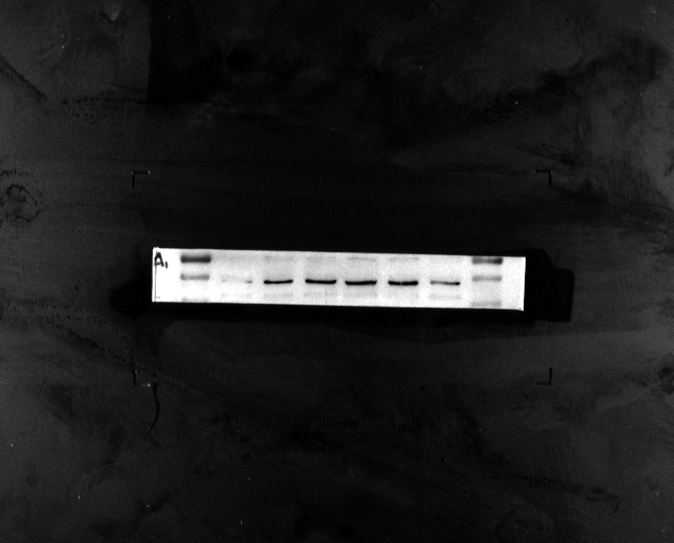

Supplement: Supplementary file 2 [file DataSheet1.ZIP › data sheet for review purpose only (1)/Original date for review purpose only (1)/western blot/Figure 2/Figure 2-pAMPK-backgroud.tif]

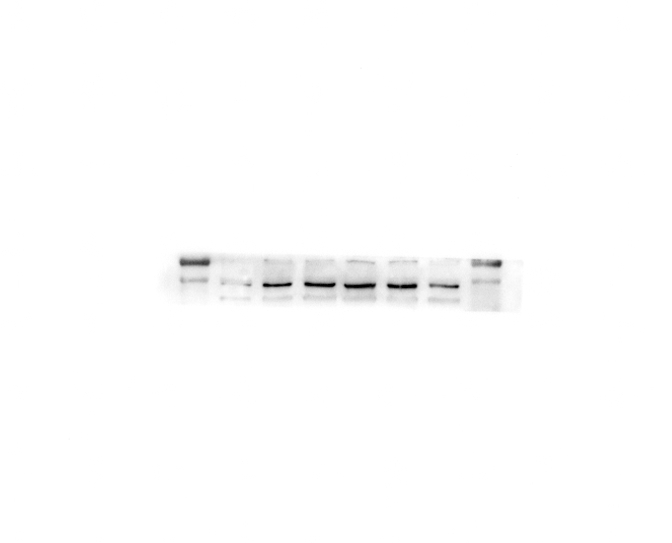

Supplement: Supplementary file 2 [file DataSheet1.ZIP › data sheet for review purpose only (1)/Original date for review purpose only (1)/western blot/Figure 2/Figure 2-pAMPK.tif]

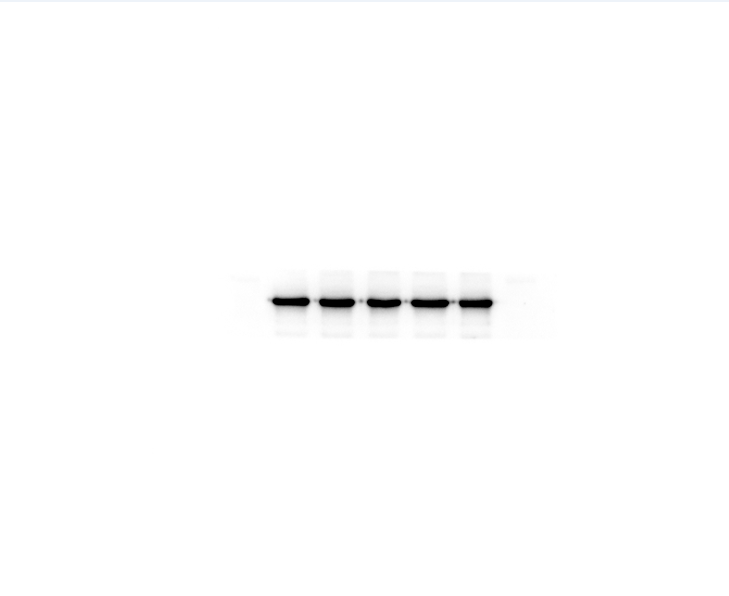

Supplement: Supplementary file 2 [file DataSheet1.ZIP › data sheet for review purpose only (1)/Original date for review purpose only (1)/western blot/Figure 4/Figure 4-B-actin.tif]

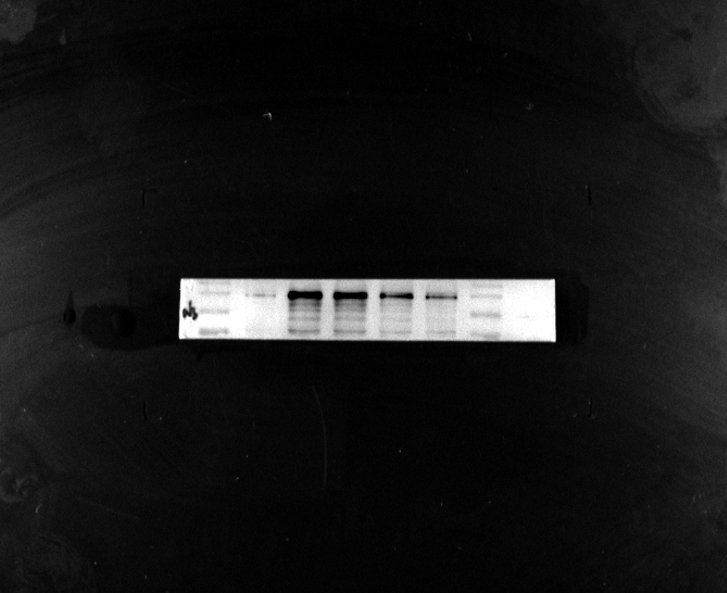

Supplement: Supplementary file 2 [file DataSheet1.ZIP › data sheet for review purpose only (1)/Original date for review purpose only (1)/western blot/Figure 4/Figure 4-NLRP3-backgroud.tif]

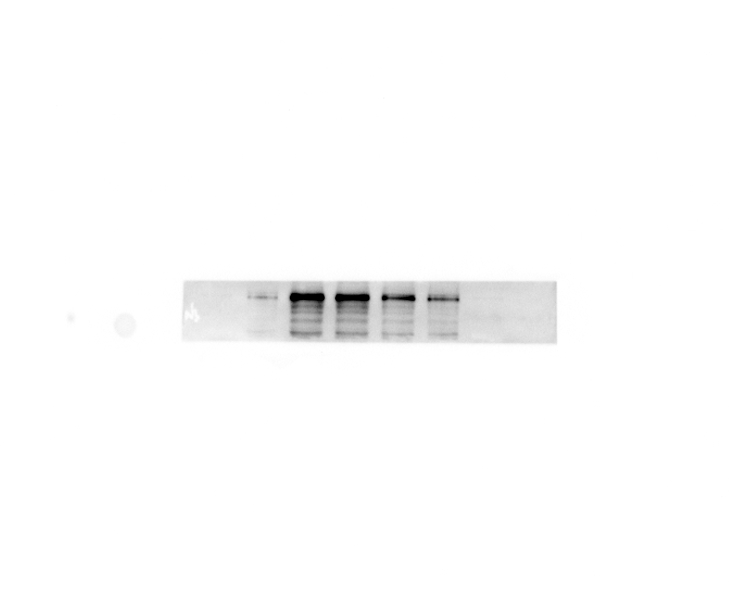

Supplement: Supplementary file 2 [file DataSheet1.ZIP › data sheet for review purpose only (1)/Original date for review purpose only (1)/western blot/Figure 4/Figure 4-NLRP3.tif]

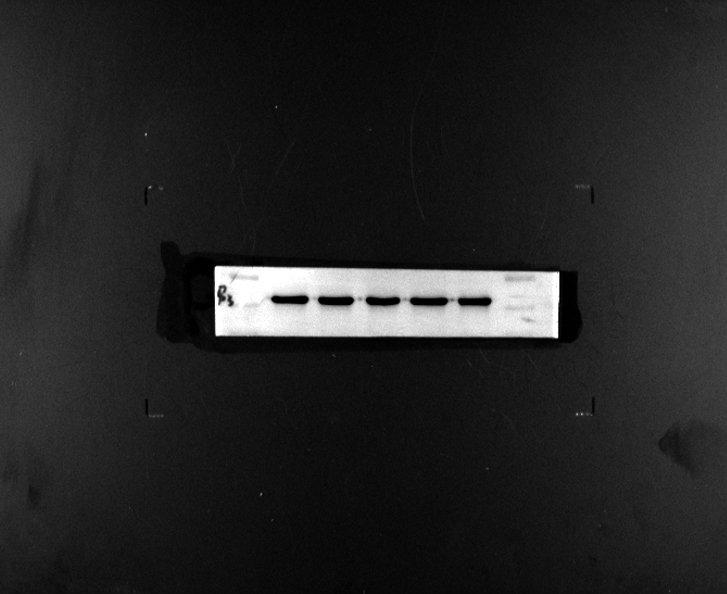

Supplement: Supplementary file 2 [file DataSheet1.ZIP › data sheet for review purpose only (1)/Original date for review purpose only (1)/western blot/Figure 4/Figure-4-B-actin-backgroud.tif]

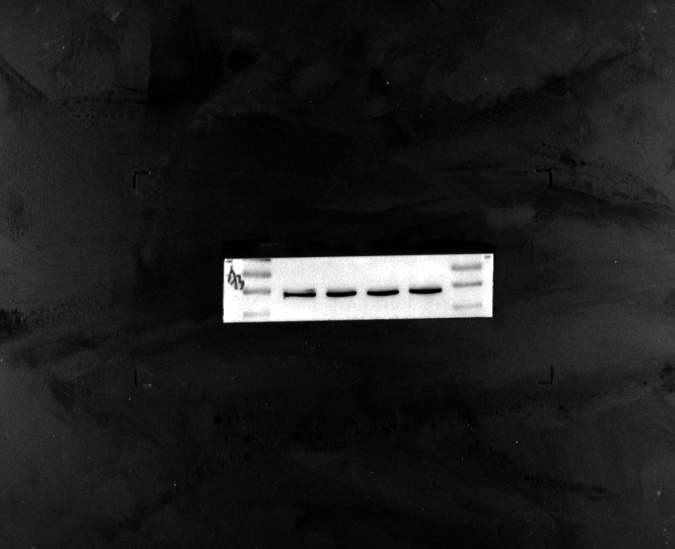

Supplement: Supplementary file 2 [file DataSheet1.ZIP › data sheet for review purpose only (1)/Original date for review purpose only (1)/western blot/Figure 6/Figure 6-AMPK-backgroud.tif]

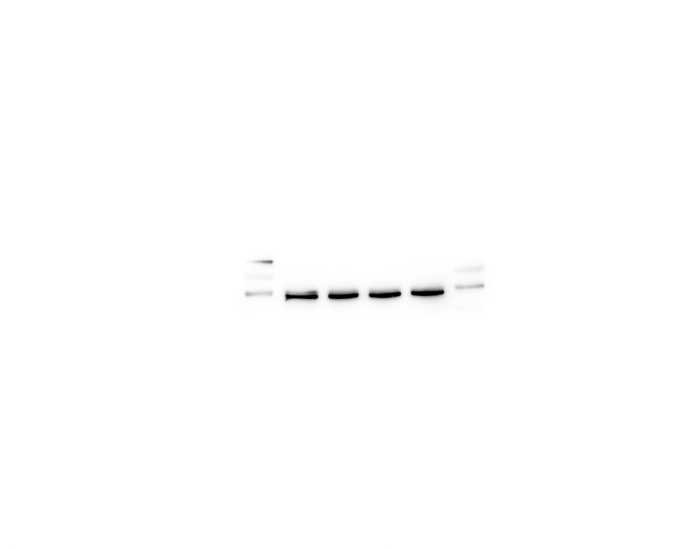

Supplement: Supplementary file 2 [file DataSheet1.ZIP › data sheet for review purpose only (1)/Original date for review purpose only (1)/western blot/Figure 6/Figure 6-AMPK.tif]

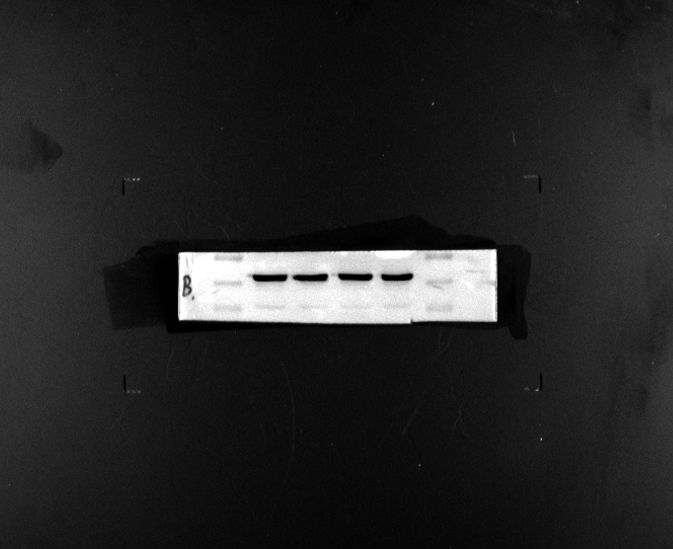

Supplement: Supplementary file 2 [file DataSheet1.ZIP › data sheet for review purpose only (1)/Original date for review purpose only (1)/western blot/Figure 6/Figure 6-B-actin-background.tif]

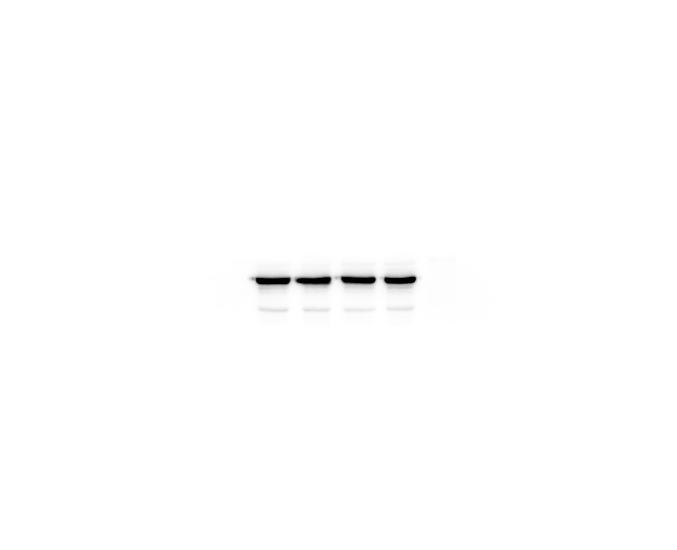

Supplement: Supplementary file 2 [file DataSheet1.ZIP › data sheet for review purpose only (1)/Original date for review purpose only (1)/western blot/Figure 6/Figure 6-B-actin.tif]

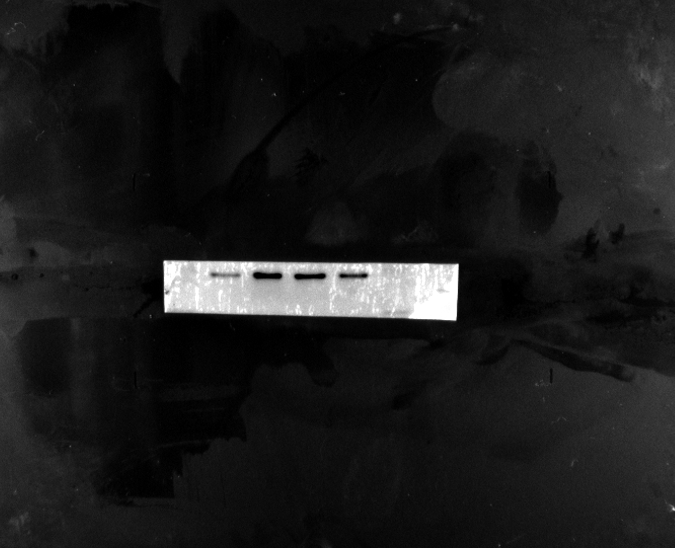

Supplement: Supplementary file 2 [file DataSheet1.ZIP › data sheet for review purpose only (1)/Original date for review purpose only (1)/western blot/Figure 6/Figure 6-IL-18-background.tif]

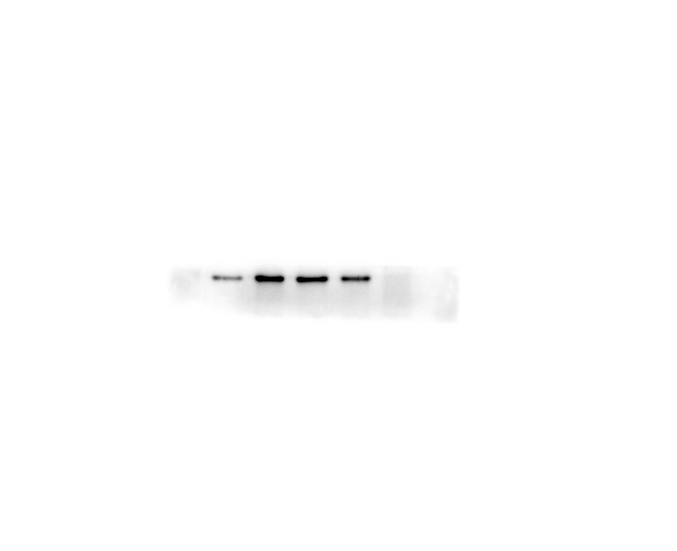

Supplement: Supplementary file 2 [file DataSheet1.ZIP › data sheet for review purpose only (1)/Original date for review purpose only (1)/western blot/Figure 6/Figure 6-IL-18.tif]

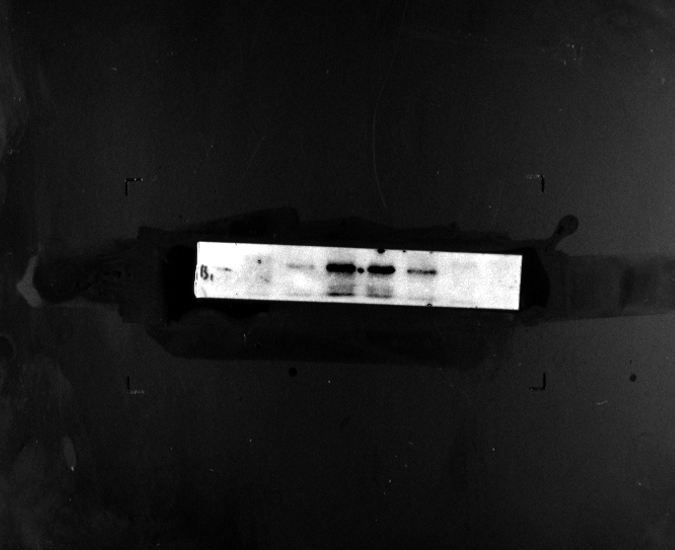

Supplement: Supplementary file 2 [file DataSheet1.ZIP › data sheet for review purpose only (1)/Original date for review purpose only (1)/western blot/Figure 6/Figure 6-IL-1B-background.tif]

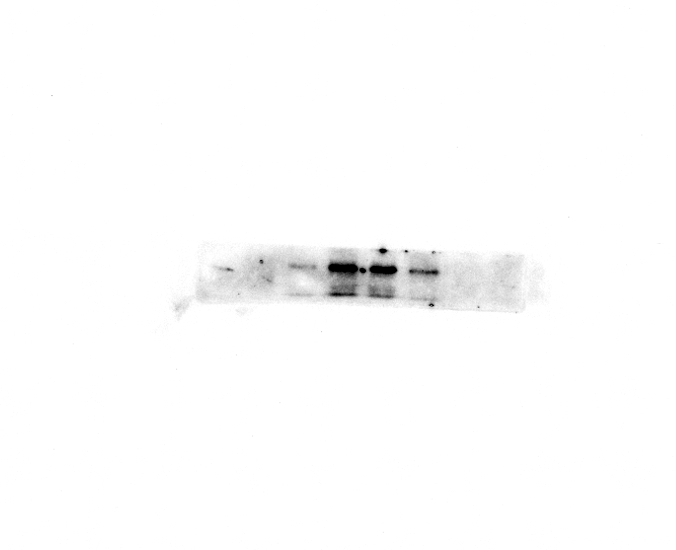

Supplement: Supplementary file 2 [file DataSheet1.ZIP › data sheet for review purpose only (1)/Original date for review purpose only (1)/western blot/Figure 6/Figure 6-IL-1B.tif]

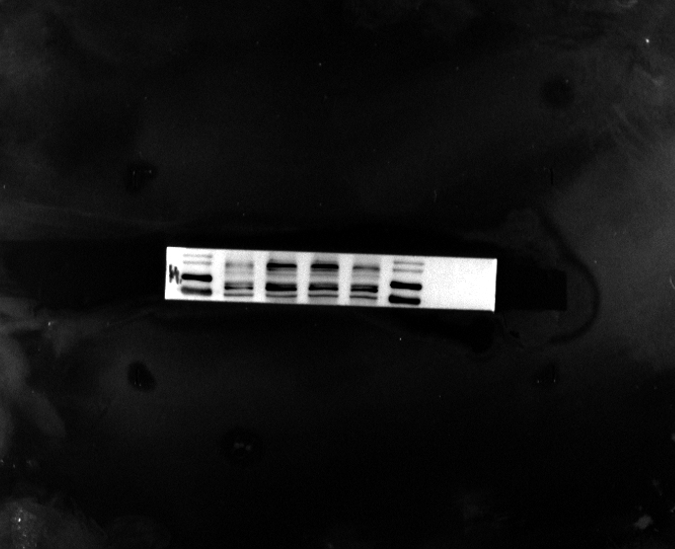

Supplement: Supplementary file 2 [file DataSheet1.ZIP › data sheet for review purpose only (1)/Original date for review purpose only (1)/western blot/Figure 6/Figure 6-NLRP3-backgroud.tif]

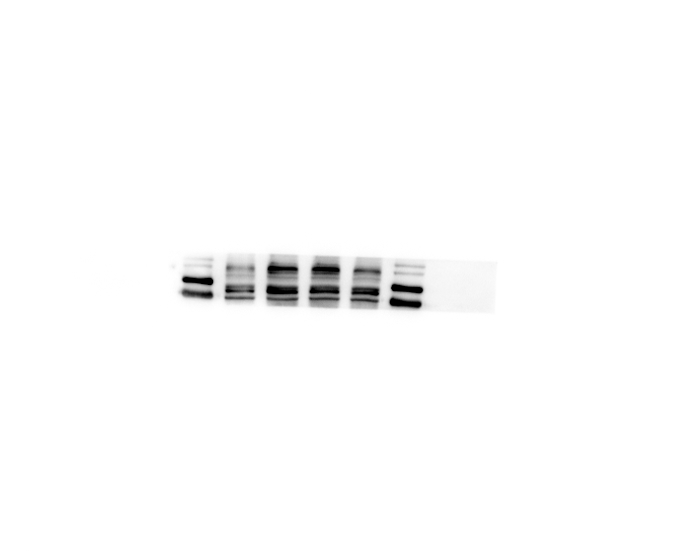

Supplement: Supplementary file 2 [file DataSheet1.ZIP › data sheet for review purpose only (1)/Original date for review purpose only (1)/western blot/Figure 6/Figure 6-NLRP3.tif]

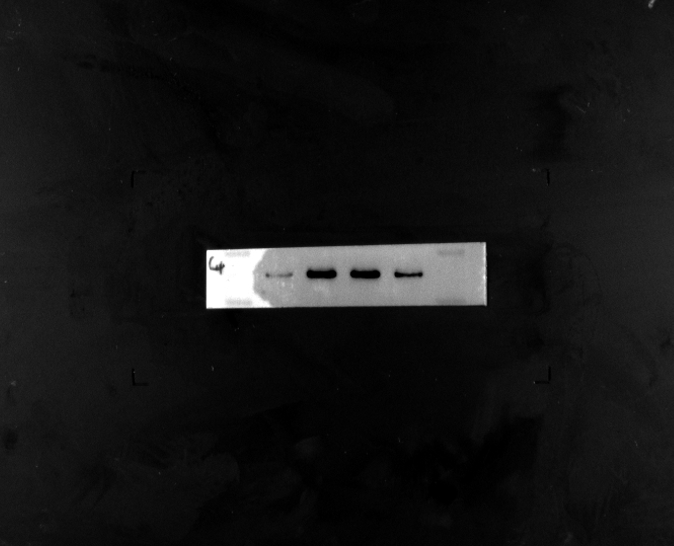

Supplement: Supplementary file 2 [file DataSheet1.ZIP › data sheet for review purpose only (1)/Original date for review purpose only (1)/western blot/Figure 6/Figure 6-cleaved caspase-1-background.tif]

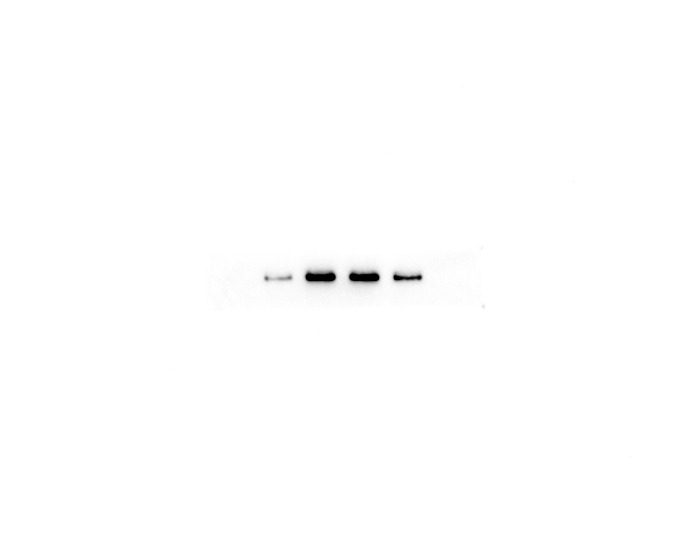

Supplement: Supplementary file 2 [file DataSheet1.ZIP › data sheet for review purpose only (1)/Original date for review purpose only (1)/western blot/Figure 6/Figure 6-cleaved caspase-1.tif]

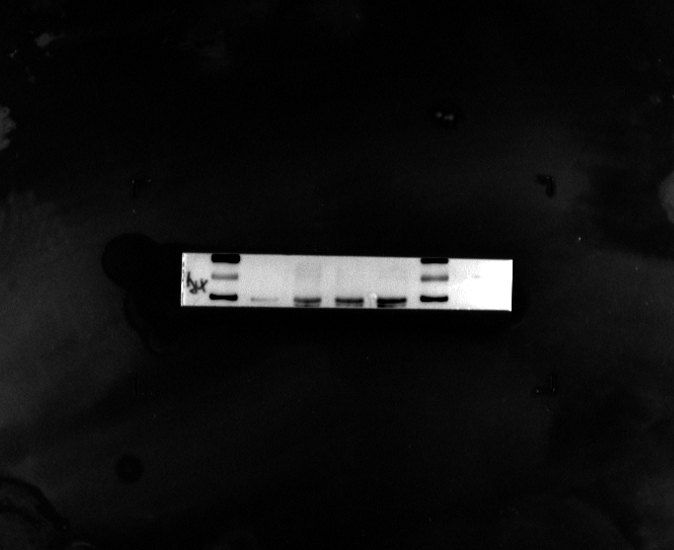

Supplement: Supplementary file 2 [file DataSheet1.ZIP › data sheet for review purpose only (1)/Original date for review purpose only (1)/western blot/Figure 6/Figure 6-pAMPK-backgroud.tif]

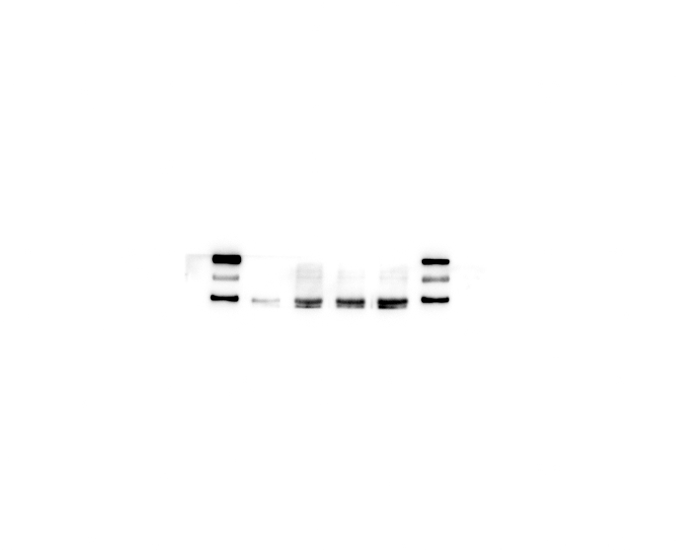

Supplement: Supplementary file 2 [file DataSheet1.ZIP › data sheet for review purpose only (1)/Original date for review purpose only (1)/western blot/Figure 6/Figure 6-pAMPK.tif]

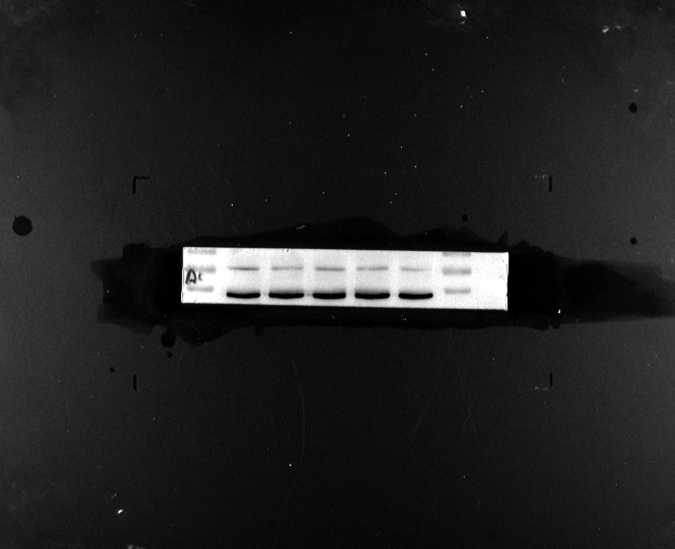

Supplement: Supplementary file 2 [file DataSheet1.ZIP › data sheet for review purpose only (1)/Original date for review purpose only (1)/western blot/Figure 7/Figure 7-AMPK-background.tif]

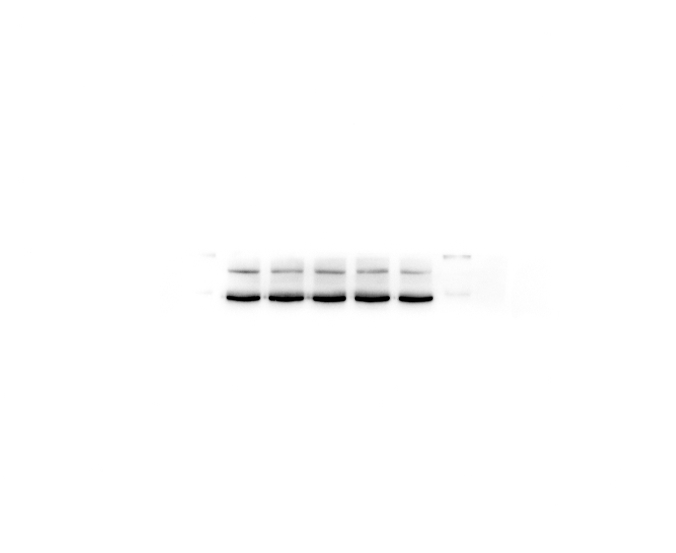

Supplement: Supplementary file 2 [file DataSheet1.ZIP › data sheet for review purpose only (1)/Original date for review purpose only (1)/western blot/Figure 7/Figure 7-AMPK.tif]
