# Supplementary figures and images for: Metformin Inhibits NLR Family Pyrin Domain Containing 3 (NLRP)-Relevant Neuroinflammation via an Adenosine-5′-Monophosphate-Activated Protein Kinase (AMPK)-Dependent Pathway to Alleviate Early Brain Injury After Subarachnoid Hemorrhage in Mice (part 2 of 2)
Source: Front Pharmacol. 2022 Mar 17;13:796616. doi: 10.3389/fphar.2022.796616 (PMC8969021; doi:10.3389/fphar.2022.796616)

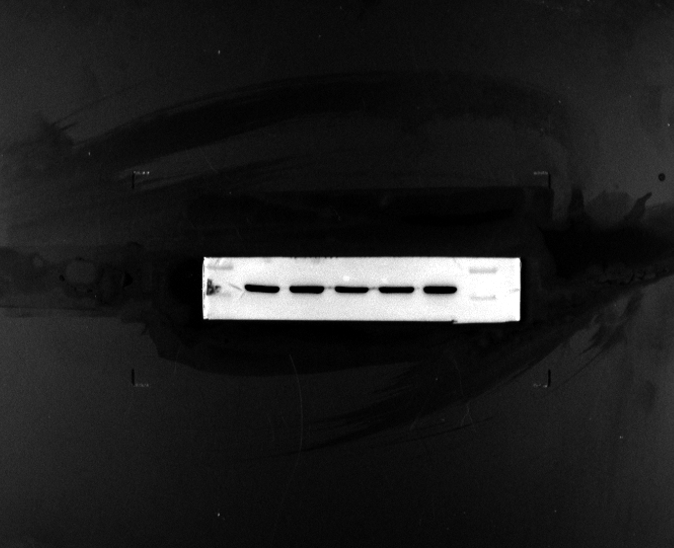

Supplement: Supplementary file 2 [file DataSheet1.ZIP › data sheet for review purpose only (1)/Original date for review purpose only (1)/western blot/Figure 7/Figure 7-B-actin-background.tif]

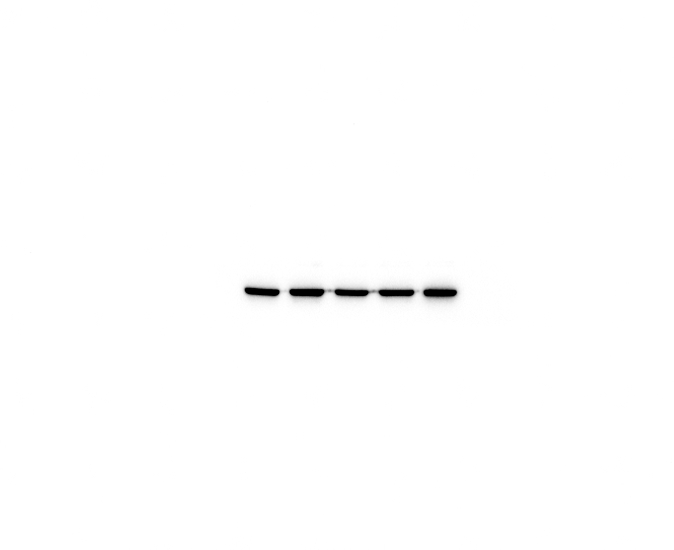

Supplement: Supplementary file 2 [file DataSheet1.ZIP › data sheet for review purpose only (1)/Original date for review purpose only (1)/western blot/Figure 7/Figure 7-B-actin.tif]

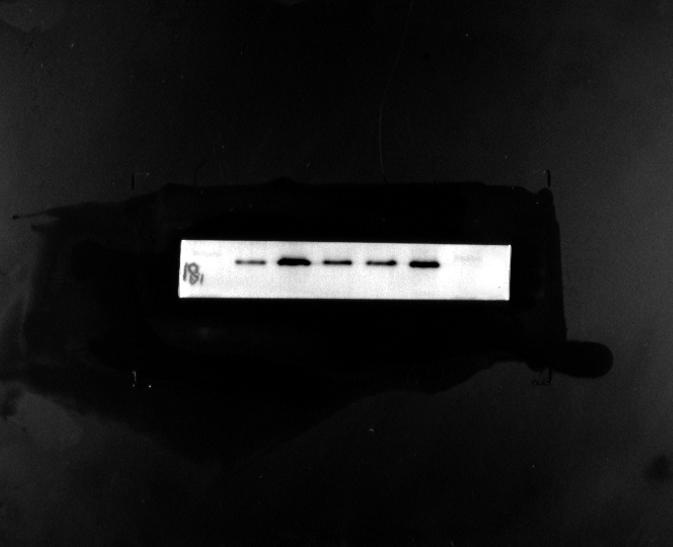

Supplement: Supplementary file 2 [file DataSheet1.ZIP › data sheet for review purpose only (1)/Original date for review purpose only (1)/western blot/Figure 7/Figure 7-IL-18-background.tif]

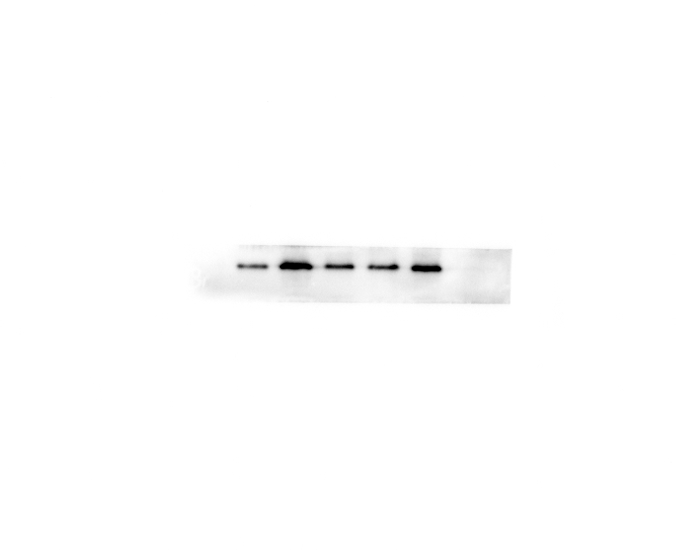

Supplement: Supplementary file 2 [file DataSheet1.ZIP › data sheet for review purpose only (1)/Original date for review purpose only (1)/western blot/Figure 7/Figure 7-IL-18.tif]

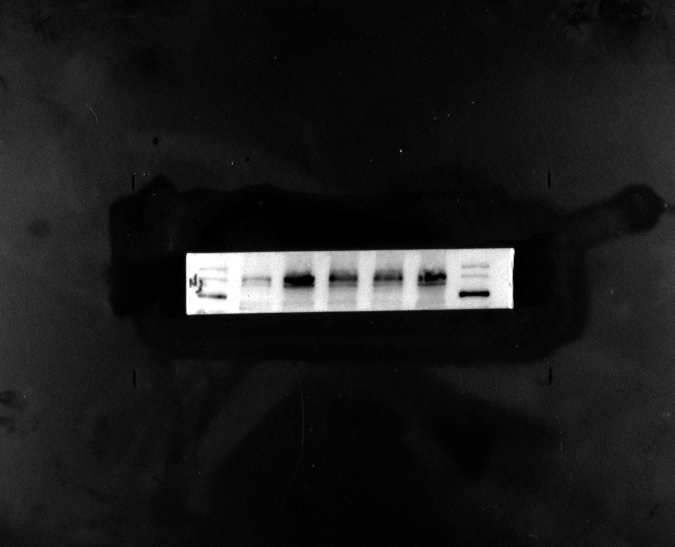

Supplement: Supplementary file 2 [file DataSheet1.ZIP › data sheet for review purpose only (1)/Original date for review purpose only (1)/western blot/Figure 7/Figure 7-NLRP3-background.tif]

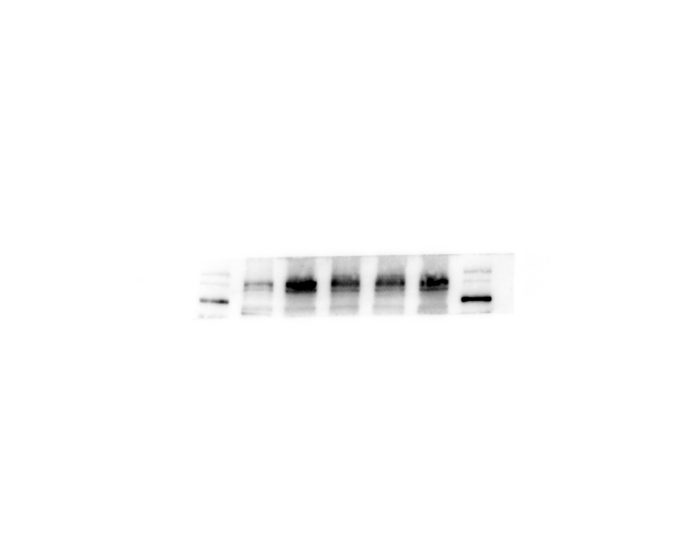

Supplement: Supplementary file 2 [file DataSheet1.ZIP › data sheet for review purpose only (1)/Original date for review purpose only (1)/western blot/Figure 7/Figure 7-NLRP3.tif]

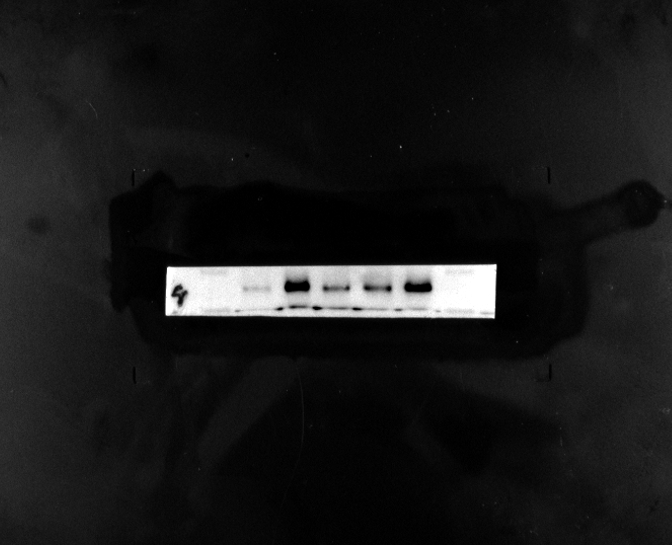

Supplement: Supplementary file 2 [file DataSheet1.ZIP › data sheet for review purpose only (1)/Original date for review purpose only (1)/western blot/Figure 7/Figure 7-cleaved caspase-1-background.tif]

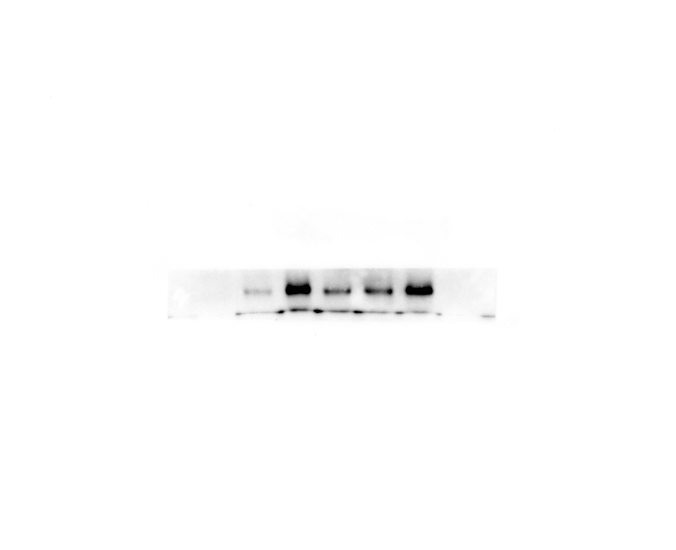

Supplement: Supplementary file 2 [file DataSheet1.ZIP › data sheet for review purpose only (1)/Original date for review purpose only (1)/western blot/Figure 7/Figure 7-cleaved caspase-1.tif]

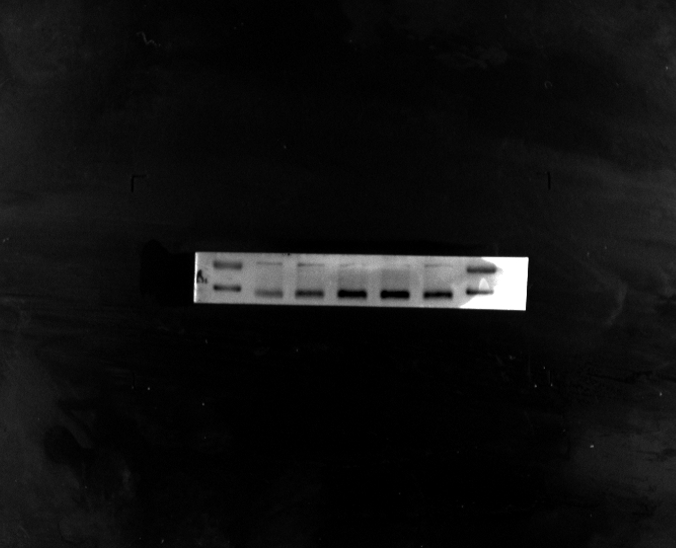

Supplement: Supplementary file 2 [file DataSheet1.ZIP › data sheet for review purpose only (1)/Original date for review purpose only (1)/western blot/Figure 7/Figure 7-pAMPK-background.tif]

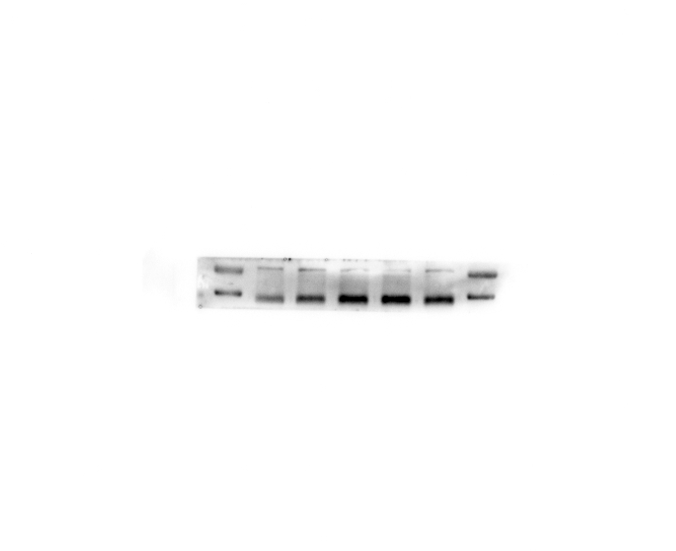

Supplement: Supplementary file 2 [file DataSheet1.ZIP › data sheet for review purpose only (1)/Original date for review purpose only (1)/western blot/Figure 7/Figure 7-pAMPK.tif]

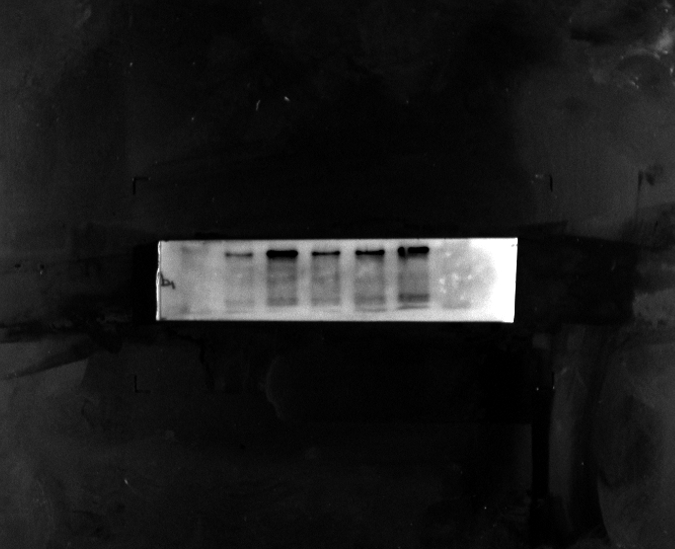

Supplement: Supplementary file 2 [file DataSheet1.ZIP › data sheet for review purpose only (1)/Original date for review purpose only (1)/western blot/Figure 7/Figure-7-IL-1B-background.tif]

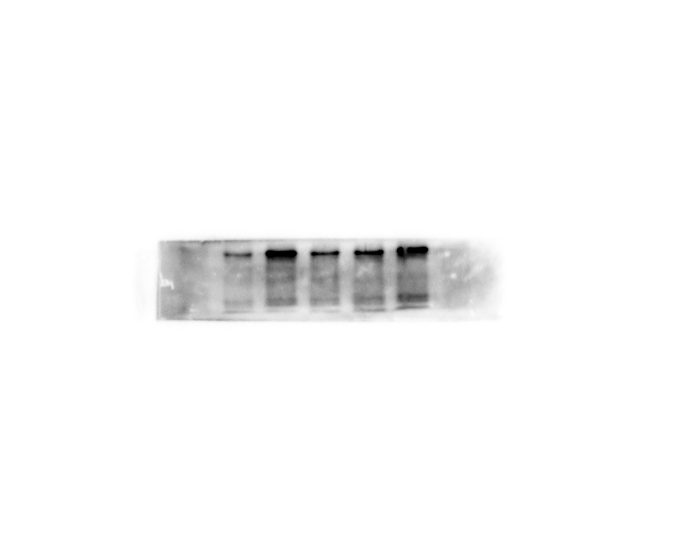

Supplement: Supplementary file 2 [file DataSheet1.ZIP › data sheet for review purpose only (1)/Original date for review purpose only (1)/western blot/Figure 7/Figure-7-IL-1B.tif]

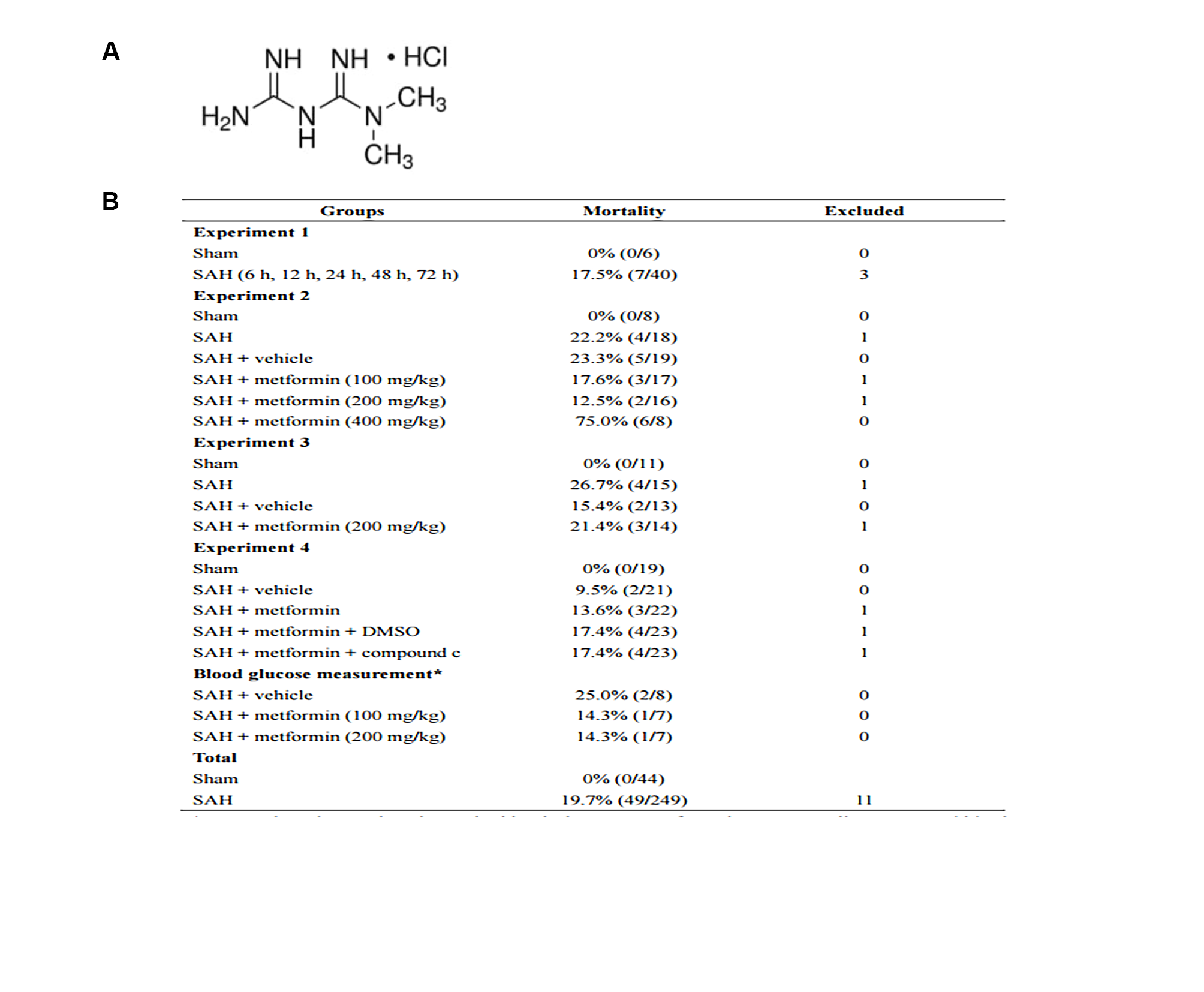

Supplement: Supplementary file 4 [file Image1.TIF]

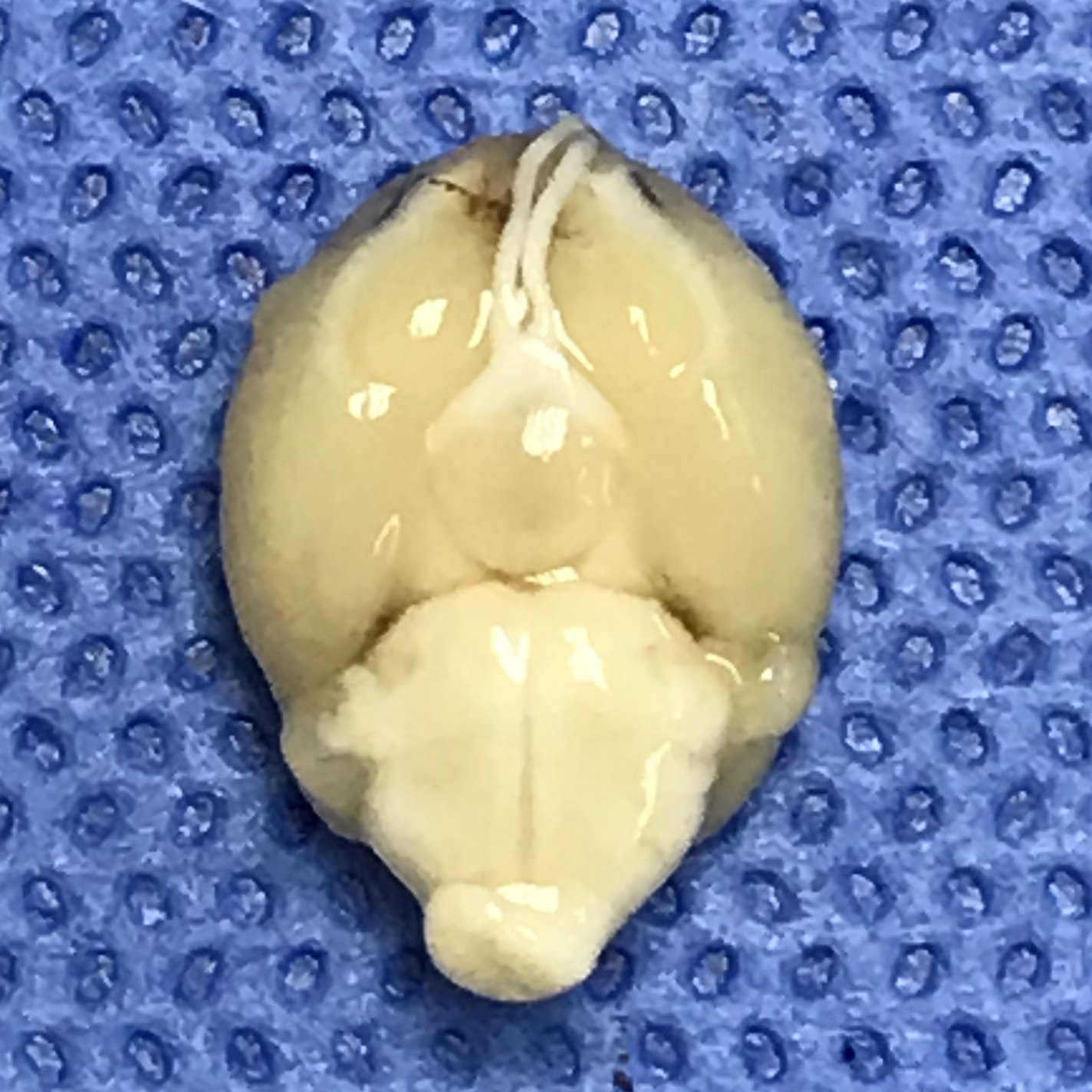

Supplement: Supplementary file 5 [file DataSheet2.ZIP › data sheet for review purpose only (2)/Original date for review purpose only (2)/Figure 2A.tif]

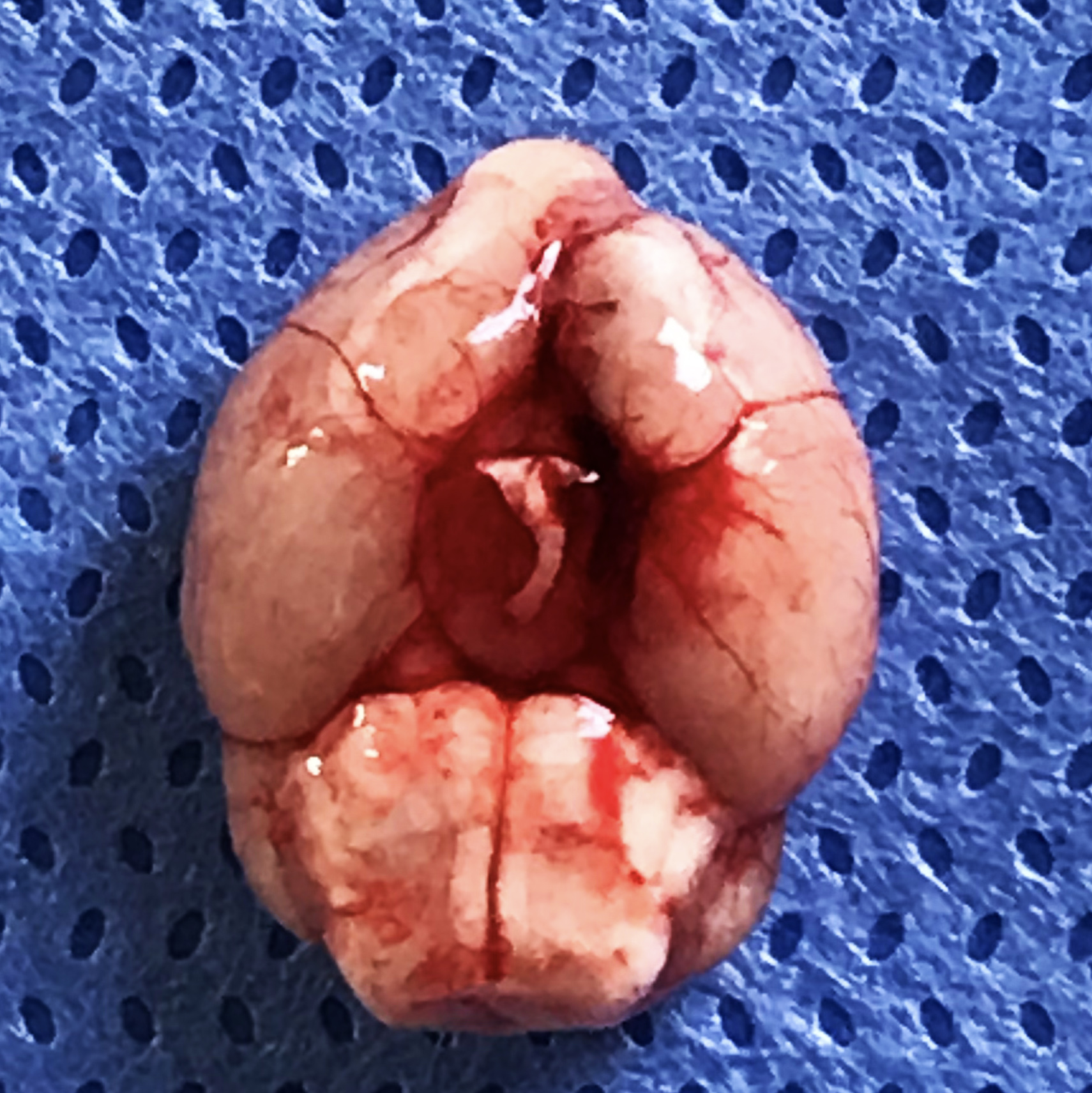

Supplement: Supplementary file 5 [file DataSheet2.ZIP › data sheet for review purpose only (2)/Original date for review purpose only (2)/Figure 2B.tif]
